# Butchering knives and hafting at the Late Middle Paleolithic open-air site of Nahal Mahanayeem Outlet (NMO), Israel

**DOI:** 10.1038/s41598-022-27321-5

**Published:** 2023-01-03

**Authors:** Juan Ignacio Martin-Viveros, Maya Oron, Andreu Ollé, M. Gema Chacón, Gonen Sharon

**Affiliations:** 1 Institut Català de Paleoecologia Humana i Evolució Social (IPHES-CERCA), Zona Educacional 4, Campus Sescelades URV (Edifici W3), 43007 Tarragona, Spain; 2 Departament d’Història i Història de l’Art, Universitat Rovira i Virgili (URV), Avinguda Catalunya 35, 43002 Tarragona, Spain; 3 Archaeological Research Department, Israel Antiquities Authority, POB 586, 91004 Jerusalem, Israel; 4 Institute of Archaeology, The Hebrew University of Jerusalem, Mt. Scopus, 91905 Jerusalem, Israel; 5 UMR7194 – HNHP (CNRS – MNHN –UPVD – Sorbonne Universités), 1 rue Renè Panhard, 75013 Paris, France; 6 Musée de l’Homme, 17 Place du Trocadéro, 75016 Paris, France; 7 Department of Galilee Studies (M.A.), Tel-Hai College, Upper Galilee, Israel

**Keywords:** Archaeology, Anthropology

## Abstract

Much of what is known about human behavior and subsistence strategies in the Levantine Middle Paleolithic comes from long sequences from caves and rock shelters. In this context, studies of stone tool function have traditionally focused on determining the use of Levallois points and triangular elements, either as projectiles or, more rarely, multipurpose knives. Little is known about such tool use and hafting in Middle Paleolithic open-air sites in the Levant through the systematic application of micro-wear analysis. Here we report the results of a low and high-power study performed on the lithic assemblage of the Late Middle Paleolithic open-air site of Nahal Mahanayeem Outlet (NMO, Israel). Most pointed items, including Levallois and non-Levallois points, were used as butchering knives, many of them while hafted; to a much lesser extent they were also used for hide, bone, and wood/plant processing activities. Blades and flakes were mostly handheld and used as butchering knives, with hide, bone, antler, and wood/plant-processing tasks being rare. Hafted artifacts include morphologies and activities for which hafting is not required, indicating that NMO inhabitants possessed varied hafting expertise. Wood/plant processing tools, some of which were hafted, attest that manufacture and maintenance tasks were planned well in advance of game procurement at the site. These results attest to early evidence of hafted butchering knives and hafted plant processing tools for a Late Middle Paleolithic open-air site in the Levant, and support previous interpretations of NMO as a short-term task-specific location focused on animal processing activities, mostly butchery.

## Introduction

The Middle Paleolithic (MP) of the Levant is a key period for understanding human evolution, dispersal, and behavior. After more than 100 years of research, most of our knowledge regarding this period is based on data retrieved from cave sites. The long and well-stratified sequences of cave sites enabled researchers to establish the chronological framework for the cultural sequence of the Levant^1^. Following ethnographic-derived models^2,3^, much emphasis was placed on assessing the effects of technological organization on lithic assemblage variability—within and between sites—to understand the techno-economic behaviors practiced by humans for their land use management strategies. Variability observed in lithic procurement, knapping strategies, toolkit composition, and reduction intensity between the different lithic assemblages was compared to models based upon ethnographic observation of modern hunter-gatherers. Two strategies of technological organization have been widely applied to explain the depth of planning required in the procurement, production, transport, maintenance, and use of stone tools to exploit a territory’s critical resources: provisioning of individuals and provisioning of places^4–6^. In the former strategy, lithic production is oriented toward manufacturing readily usable and maintainable tools or personal gear^2^ for responding to unexpected situations while moving through a territory, especially when predictability of raw material sources is low. In contrast, if the predictability of raw material availability for a given territory is high, then the provisioning of places with raw material would indicate the anticipation of specific activities in these places. Because raw material is abundant, manufacturing formal and high-maintenance tools would be inefficient due to the high technological cost in time and energy of this strategy.

While sometimes disputed, the strategies of provisioning of individuals and provisioning of places are a valuable interpretative tool for the study of site function, settlement systems, and mobility patterns of past hunter-gatherers. This is especially true when combined with robust archaeological data such as integrity of reduction sequences, refitting, faunal and vegetal resource management, and differentiated use of space. Moreover, since land use management strategies of modern hunter-gatherers were probably influenced by ecological constraints, seasonality, and socio-cultural traditions^7,8^, prehistoric hunter-gatherers are expected to have applied mixed scenarios of technological strategies according to such factors, resulting in complex archaeological realities. Accordingly, many Levantine MP cave sites were interpreted as home-base camps^9–12^, with layers usually indicating long-term^11^ but also ephemeral occupations^13,14^. Based on the diversity of the toolkit component and technology (e.g., frequency of retouched artifacts, intensity of retouch, and proportion of debitage by-products and cores), lithic assemblages from Levantine caves were considered evidence of either logistical strategies, such as provisioning of places, or foraging strategies, such as provisioning of individuals. Most cave sites and rock shelters, are usually assumed to have been long-term residential camps, with their lithic toolkits showing a marked expedient character, compatible with a variety of activities.

Such debates demonstrate the importance of use-wear studies for determining tool function. Analyses of Early^15,16^ and especially Late MP cave assemblages^17–19^ have focused on retouched and unretouched Levallois points and triangular elements. These studies sparked a use-wear analysis debate as to whether Levallois points and triangular elements were primarily used as spear points for hunting^20^ or for the production of multipurpose tools for a variety of tasks like butchery, plant processing or perforating^21–25^. Shea et al. argued that the elongated and thin Levallois points of the Early MP, such as the ones from Tabun Cave layer IX, Abu Sif layers B and C, and Ain Difla, were used as cutting implements, since they are not sufficiently strong to withstand impact damage if used as projectiles^26^. On the other hand, the short-broad Levallois points of Late MP assemblages, such as the ones from Tabun Unit I, Skhul layer B, Qafzeh layers I-XXIV, and Kebara Cave layers VIII-XIII, are better suited as spear points, providing greater resistance to impact. In contrast, recent use-wear studies have stressed the multi-purpose nature of Early MP Levallois points and other elongated pointed items^27,28^, but see^29^. Tool function data for additional tool types typical of Levantine MP assemblages, like side-scrapers, denticulates, notches, and even ordinary flakes and blades—are scarce for Levantine caves and rock shelters^28^.

Because of the scarcity of tool function data, hafted stone artifacts from Levantine MP cave sites are not commonly identified. Hafting analysis can indirectly inform about organic materials used to elaborate the handles, which are rarely preserved. Tool standardization traits related to hafting, such as shaping retouch, basal thinning, or bilateral notching, can be verified through comprehensive macro and microscopic identification of hafting traces. The elaboration of hafting for different tool functions may require different morphological adjustments on the stone artifact to fit a certain type of haft^30–38^. Stone artifacts used in activities that require a handle or shaft, such as projectile weapons or hafted stone axes, should be distinguished from those for which a handle may enhance tool efficiency but is not a necessity^30,39,40^. Direct evidence for hafting (macro and micro-wear traces) on stone artifacts used in activities for which hafting is not mandatory are rarely reported for Levantine MP cave sites^27,28^. The majority of evidence has been presented for projectile weapons—for which hafting is a necessity—and is indirect (e.g., retouch patterns, basal thinning, lateral modifications) or based on low-power magnifications^15,20^. Direct evidence, such as that reported for European MP^39^ and Middle Stone Age assemblages^32,41^ is still absent for the Levant.

Open-air sites are rare in the Levantine MP, particularly from the early MP^42^. In the Mediterranean climatic zone, the environmental setting of most Levantine MP open-air sites is reconstructed as lake-shore or floodplain^43–46^, whereas in more arid environments sites are located near springs^47–51^. Typically, Levantine MP open-air sites have short stratigraphic sequences and constrained lithic and faunal records. Hence, Levantine MP open-air sites are often interpreted as short-term, task-specific localities in which the higher frequencies of retouched items and the low frequencies of debris, cortical primary flakes, and cores enabled identification of imported finished products^52–55^, and, therefore, the provisioning of individuals^56^. A different scenario has been proposed for the late MP open-air site of Ein Qashish, where the relatively low density of lithic artifacts and the low frequency of retouched items enabled interpretation of the site as a short-term, residential occupation, where more generalized activities took place^57^. Other MP open-air sites, such as Nesher Ramla, yielded rich assemblages with high densities of lithic remains, similar to those seen in caves. At Nesher Ramla, correlation between changes in lithic artifact density with shifts in techno-typological composition fit well with a mixed scenario of task-specific and more generalized occupation^58^. Use-wear analysis results from unit III of the site demonstrated that diverse tools, such as naturally backed knives, Nahr Ibrahim tools, and elongated large tranchet blow spalls, were primarily used to process animal carcasses, namely butchery and bone-working activities. No evidence of projectile weapons was observed^59^.

Aside from Nesher Ramla, use-wear data combining low-power and high-power magnifications are only available for the Late MP open-air site of Umm el Tlel. Results of tool function analysis revealed that 56% of the Levallois points and triangular flakes were used as butchering knives, while an additional 31% of the items were used for cutting indeterminate soft material, and only 10% for cutting non-woody plants, such as reeds^21^. Although use-wear evidence of projectile weapons was not found, a Levallois point embedded in a vertebra of wild ass demonstrates the presence of projectiles at Umm el Tlel^22^. Projectiles were also identified in the small assemblage of pointed items at the Late MP open-air site of Boker Tachtit using low-power magnifications^60^. Based upon macro-scars and intentional modifications observed on the proximal and lateral edges, hafting was claimed for these pointed items. Based on indirect evidence, hafting^61^, and even identification of adhesives^62–65^, has been assessed for other Levantine MP open-air sites. The current state of research demonstrates that Levantine MP open-air site assemblages await systematic and comprehensive use-wear studies that incorporate both low-power and high-power magnifications.

The current study presents the results of low and high-power use-wear analysis carried out on the lithic assemblage of the late MP open-air site of Nahal Mahanayeem Outlet (NMO), a lake-shore, open-air site excavated on the bank of the Upper Jordan River. The site was interpreted as a short-term, task specific hunting locality^66^ based on the low density of lithic artifacts, the scarcity of tool types typical of MP assemblages (e.g., scrapers), the high frequency of retouched artifacts, and the low presence of debris and debitage. NMO layers are waterlogged, enabling exceptional preservation of organic remains, including bones, wood fragments, fruits and seeds (full details on SI). The NMO flint tool assemblage is exceptionally small in comparison to other MP Levantine sites. The typological combination of the assemblage stands out among Levantine MP assemblages comprising an exceptional number of pointed elements and elements with significant cutting edge (Fig. S1)^66^. Together, these factors make NMO an ideal site to apply low and high-power use-wear analysis with a resolution rarely achieved in other Levantine assemblages. NMO is a unique short-term open-air site for testing the Middle Paleolithic task-specific hunting hypothesis and gaining insights into tool use and hafting in Levantine MP open-air sites. Use-wear analysis carried out on the NMO lithic assemblage enabled testing the actual function of pointed elements, including Levallois and non-Levallois points and defining them as specialized or multi-purpose tools. The function of expedient items with significant cutting edge, such as naturally backed knives (NBK), denticulates and retouched and unretouched flakes, was also tested. The strategies behind the selection of elongated blanks for the production of points and tools were assessed according to their function. In addition, the role of plant processing as part of the complementary activities that accompany the processing of animal carcasses was investigated. The study results deepened our understanding of the technological aspects of tool use, such as hafting and its effect on lithic production and use processes at NMO.

## The NMO site

**Figure 1:**
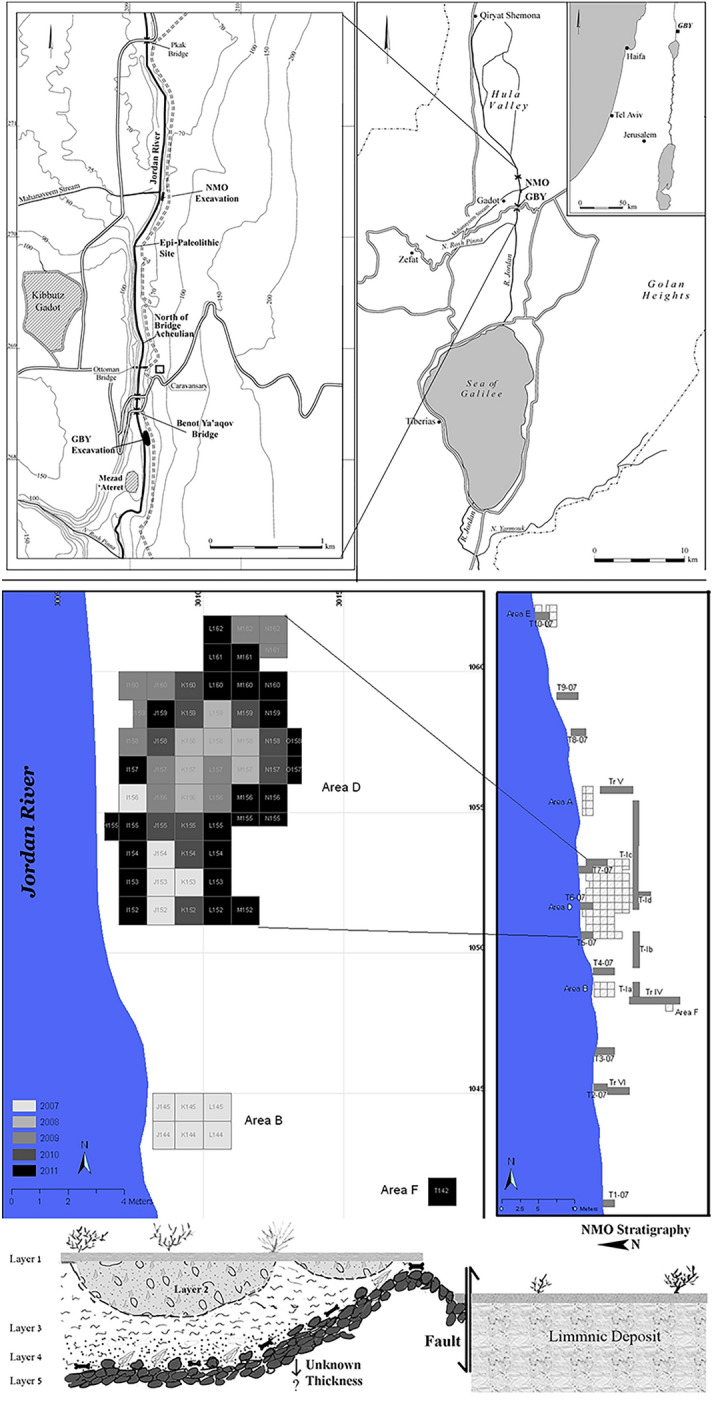
Location of the NMO site, excavation areas, excavated squares, and schematic representation of the stratigraphy.

Nahal Mahanayeem Outlet (NMO) is located on the east bank of the Upper Jordan River, at its outflow south of the Hula Valley (Fig. 1). The site was discovered in 1999 during intensive drainage works^67^ and was excavated between 2007 and 2014. The geological sequence of the Upper Jordan River in the vicinity of the site is highly complex due to massive tectonics and volcanism^68–70^, yet the stratigraphy of NMO is relatively straightforward^71^. At the base of the sequence lies a layer of basalt boulders and cobbles of fluvial origin (Layer 5). The basalt forms a small hill with the highest point at the southwest part of the excavated area (Fig. 1). The primary archaeological deposit of NMO is embedded in fine black silty mud (Layer 4), resting on top of this basalt “hill”, with the artifacts and bones found lying on top of the basalt floor as well as in the mud above it. The thickness of the artifact and bone-bearing layer reaches up to 40 cm. The faunal remains of Layer 4 are comprised of large wild cattle (*Bos primigenius*), some found in near-articulated position, as well as a variety of other species such as wild boar and gazelle, and smaller game and micro-fauna^72,73^. A series of OSL dates set Layer 4 to c. 60 ka BP^71,74^. This date places NMO within the Levant's final phase of the Middle Paleolithic^55,75^.

On top of archaeological Layer 4 is a sequence of silty mud and clays that accumulated during the Late Pleistocene and Holocene. This sequence comprises later archaeological entities such as the Upper Paleolithic assemblage excavated from Area A at the site^46^ and, higher up in the sequence, even Byzantine coins and lead fishing net weights. The primary NMO archaeological horizon—Layer 4 was sealed by the upper mud and was probably never subject to significant post-depositional movement (see section 1.1 on SI). Our analysis indicates that this event was followed by an additional, less intensive visit to the site that is less well preserved and left only sparse evidence in the form of a few stone tools and bones. This second event was recorded as Layer 4a; the primary event is named Layer 4b and is the significant layer studied here.

## Results

All items from layer 4b, debitage and retouched artifacts, were screened using a two-step sampling procedure to select the most suitable items for use-wear analysis (full details in the materials and methods section and Table 1). After this screening procedure, a total of 129 artifacts were selected for detailed analysis using low and high-power magnifications. The artifacts in the sample are divided into two morphological categories: pointed (n = 43) and cutting elements (n = 86) (Table 2). Pointed elements and cutting implements form a significant part of the NMO lithic assemblage. Pointed elements are shaped on flake and blade blanks of pointed morphology ending in a pointed tip. Cutting elements are flakes and blades typified by a long (> 5 cm) cutting-edge^66^. Following overview of the activities, worked materials, and use motions identified in the sample, functional results are provided for the artifacts included in each morphological group, subdivided into typological categories^9,54^.

**Table 1:** Main artifact categories from NMO layer 4b that were screened as part of the functional study, along with the number of artifacts selected in the final sample and the number of artifacts that showed use-wear.

Tool type	Layer 4b	Selected	With use-wear
Blade*	72	11	7
Levallois point	24	8	7
Retouched Levallois point	9	5	2
Pseudo-Levallois point	7	2	–
Non-Levallois point	14	14	2
Mousterian point	2	1	1
Point	3	3	1
Single convex side scraper	15	1	1
Double convex side scraper	2	1	–
Double straight convex side scraper	1	1	–
Double concave-convex side scraper	2	1	–
Typical end scraper	1	1	1
Atypical end scraper	9	1	–
Levallois flake	7	2	1
Atypical Levallois flake	4	2	1
Denticulate	17	4	4
Flake	850	28	17
Retouched flake	300	12	6
Naturally backed knife	69	25	18
Atypical backed knife	5	2	–
Notch	78	4	3
Totals	1491	129	72

*Blade category includes unretouched and retouched blades.

**Table 2:** Artifacts of NMO layer 4b selected for the functional study based on the two morphological groups identified in the assemblage and subdivided by typological categories, along with the number of artifacts that showed use-wear.

Tool type	Analyzed	With use-wear
**Pointed elements**		
Blade	2	2
Retouched blade	1	1
Levallois point	8	7
Retouched Levallois point	5	2
Pseudo-Levallois point	2	–
Non-Levallois point	14	2
Mousterian point	1	1
Point	3	1
Notch	3	2
Single convex side scraper	1	1
Double convex side scraper	1	–
Double straight convex side scraper	1	–
Double concave-convex side scraper	1	–
Totals	43	19
**Cutting elements**		
Blade	4	1
Levallois flake	2	1
Atypical Levallois flake	2	1
Denticulate	4	4
Retouched blade	4	3
Flake	28	17
Retouched flake	12	6
Naturally backed knife	25	18
Atypical backed knife	2	–
Notch	1	1
Typical end scraper	1	1
Atypical end scraper	1	–
Totals	86	53
Grand total	129	72

Of the 129 artifacts analyzed from Layer 4b, 85 (66%) bear an optimal state of surface preservation suitable for study. Of these, 72 (56%) were observed to present interpretable use-wear traces (Table S4). No evidence of use-wear traces was observed on 13 of the items (10%). The remaining 44 artifacts (34%) showed wear traces, but full interpretation could not be suggested due to post-depositional modifications (PDM). These alterations include localized abrasion and gloss likely created by soil friction, in addition to bright spots with chaotic striations, and large, intrusive steep scars with irregular morphologies (Fig. S2). Excavation related friction wear (e.g., metal marks) was also recorded in a few cases, as well as friction wear possibly derived from storage^34^ (Fig. S2d). Functional interpretation of archaeological artifacts was assessed according to a reliability scale based on the association of recorded wear features and their diagnostic character in comparison to the experimental baseline (see section 3.1 on SI). Overall, functional interpretation was reliable for 53 artifacts (74%). For the remaining 19 artifacts, interpretation reliability was estimated as high (n = 9; 12%), moderate (n = 6; 8%), poor (n = 2; 3%), and uncertain (n = 2; 3%).

### Artifact function: general overview

Animal processing is the most frequent activity documented on the NMO studied artifacts (Table S5). They were identified on 58 items (81%; Table 3). Additional observed activities, all at much lower frequencies, include plant and wood processing (n = 8; 11%) and indeterminate activities observed on 6 artifacts (8%). Within animal processing activities, butchery was identified on half of the used artifacts (n = 36; 50%). Activities related to hide processing are next in frequency (n = 12; 17%), mainly indicating cutting motions (n = 10) and, in one case, scraping. A single artifact bears evidence of both cutting and scraping. Evidence for scraping of bone and antler is evident on 8 of the studied artifacts (11%). Finally, possible evidence of impact-related wear was observed in two cases, representing less than 3% of the total used artifacts in the sample (See^70^ for additional evidence of NMO impact-related wear).

**Table 3:** Functional results for the pointed and cutting elements of the archaeological sample that exhibit use-wear subdivided by typological categories, considering the worked materials and hafted tools.

Tool type	Butchery	Fresh hide	Dry hide	Bone	Antler	Butchery + bone	Dry hide + fresh hide	Projectile	Wood	Plants	Indet	Totals	Hafted
**Pointed elements**													
Blade	1 (1)	1 (1)										**2**	**2**
Retouched Blade											1 (1)	**1**	**1**
Levallois point	3 (2)		1 (1)			1		1 (1)			1 (1)	**7**	**5**
Retouched Levallois point	1							1 (1)				**2**	**1**
Non-Levallois point	1 (1)									1 (1)		**2**	**2**
Mousterian point	1											**1**	
Point											1	**1**	
Notch	2 (1)											**2**	**1**
Single convex side scraper	1 (1)											**1**	**1**
**Totals**	**10 (6)**	**1 (1)**	**1 (1)**			**1**		**2 (2)**		**1 (1)**	**3 (2)**	**19**	**13**
**Cutting elements**													
Blade									1			**1**	
Typical Levallois flake									1			**1**	
Atypical Levallois flake		1										**1**	
Denticulate	1	2								1		**4**	
Retouched blade	3 (2)											**3**	**2**
Flake	10 (1)	2	1	1		1			1		1	**17**	**1**
Retouched flake	3			1					1		1 (1)	**6**	**1**
Naturally backed knife	6 (2)	1	2	4	1	1	1 (1)		1 (1)		1	**18**	**4**
Notch				1								**1**	
Typical end scraper									1 (1)			**1**	**1**
**Totals**	**23 (5)**	**6**	**3**	**7**	**1**	**2**	**1 (1)**		**6 (2)**	**1**	**3 (1)**	**53**	**9**
**Grand total**	**33 (11)**	**7 (1)**	**4 (1)**	**7**	**1**	**3**	**1 (1)**	**2 (2)**	**6 (2)**	**2 (1)**	**6 (3)**	**72**	**22**

Significant values are in bold.

The numbers in parentheses show the total number of hafted tools for each typological category and worked material.

Considering all artifacts in the sample, cutting motions (n = 47; 65%) are dominant over scraping and whittling motions (n = 15, 21%). Three artifacts have two different edges used for cutting and scraping, and only in a single artifact the same edge was used for both cutting and scraping. Additional use motions observed include rotatory movements for activities such as boring (n = 2), and in two cases, the use motion could not be determined (n = 2).

### Pointed elements

#### Levallois points

Among pointed artifacts, butchery traces were identified on Levallois points (retouched and unretouched) more frequently than any other typological category (5 out of 9 artifacts; Table 3). On two of these Levallois points, in addition to butchery traces, macro and micro-wear hafting traces were identified. The hafting wear consists of concentrations of scars on the proximal and mesial extremities of the lateral edges. On the proximal ventral surface of one of these items (NMO11 2886), a brownish-black residue smear is visible (Fig. S3). The presence of this residue smear together with hafting scarring may suggest adhesive use during the hafting process.

**Figure 2:**
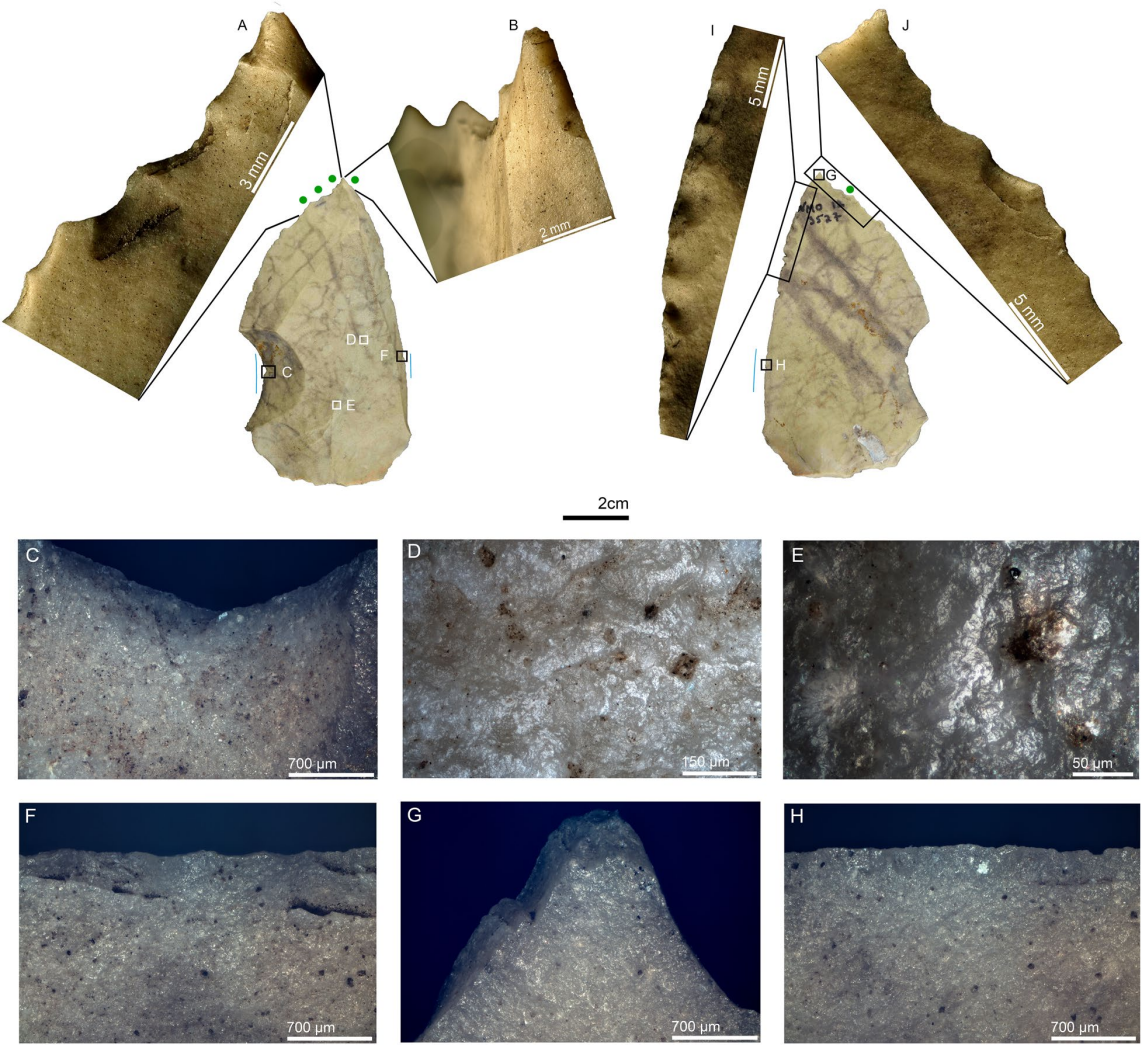
Retouched Levallois point NMO12 3527 interpreted as a projectile. (**A**) bending-initiated scars with step and feather terminations and edge crushing on the distal extremity of the dorsal left lateral edge, 35 ×; (**B**) burination that proceeds though a cascade of minor steps into a feather termination on the dorsal distal right edge, and tip crushing, 35 ×; (**C**) hafting bending-initiated scar with very shallow feather termination within the notch on the mesial dorsal left edge, 50 ×; (**D**) hafting polish on the dorsal right ridge, 200 ×; (**E**) hafting polish in a different point of the same ridge, 500 ×; (**F**) hafting scarring on the mesial dorsal right edge, 50 ×; (**G**) the burinations observed in B seen from the ventral distal right edge, and associated with tip crushing, 50 ×; (**H**) hafting scarring on the mesial ventral right edge, 50 ×; (**I**) retouch on the ventral distal right edge, 35 ×; (**J**) sliced and bending-initiated scars on the ventral distal left edge, 35 ×. *Blue solid lines indicate edge areas with hafting wear, and green dots represent edge areas with scarring. The same markers and color codes are valid for the rest of the figures.

Short Levallois points were generally identified for use as butchering knives (4 of 5 artifacts). One of these items, NMO14 1950, is a short, broad-based Levallois point with butchery and bone-scraping wear on the left and right edges, respectively (Fig. S4). Impact damage suggesting projectile use was observed on two items (NMO14 4480 and NMO12 3527). Of significance is artifact 3527, a large retouched Levallois point, which shows a combination of impact wear on the distal portion of the tool. The wear features include a burination associated with tip crushing on the dorsal distal right edge and bifacial bending-initiated scarring with step and feather terminations and oblique orientations on the dorsal left edge (Fig. 2). This association of lateral scarring with edge crushing on the dorsal left edge are considered important wear features for projectile interpretation^77,78^. The combination of wear features identified on both lateral edges, and the tip damage, supports its interpretation as a possible projectile (See^76^ for additional interpretation of the same artifact as a projectile), though butchery cannot be ruled out entirely.

Dry hide scraping wear and hafting traces were identified on elongated Levallois point NMO09 2200, consistent with its interpretation as a hafted hide-stripping knife (Fig. S5). The presence of a step-terminating bending fracture on the ventral distal tip, on the other hand, may suggest that the point had an additional function as a projectile^76^ or butchering knife, though trampling is also possible. Another Levallois point shows hafting wear on the mesial portion of both edges, though its specific function could not be determined.

#### Non-Levallois points

**Figure 3:**
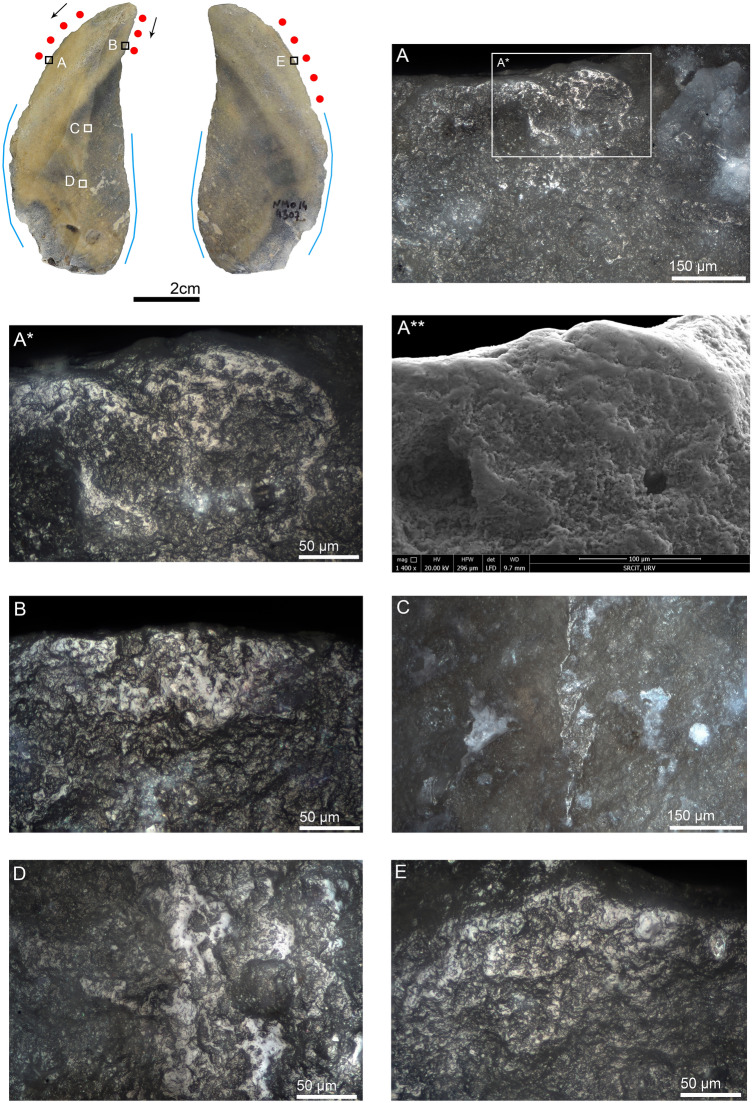
Non-Levallois point NMO14 4307 interpreted as a hafted hide-cutting knife, likely for skinning. (**A**) polish and edge rounding on the distal dorsal left edge, 200 ×; (A*) close-up of the previous image in which the rough texture and dull appearance of polish is appreciable, as well as some pits and well-developed edge rounding, 500 ×; (A**) same polish observed with SEM in which the hihgly developed polish and edge rounding are appreciable, 1400 ×; (**B**) rough-dull polish on the dorsal distal right edge, 500 ×; (**C**) hafting polish on the mesial dorsal right ridge, 200 ×; (**D**) well-developed hafting polish on the proximal dorsal right ridge, 500 ×; (**E**) rough-dull polish and edge rounding on the ventral distal left edge, 500 ×. *Edge areas with micro-wear (polish and edge rounding) are represented by red dots. Black arrows indicate the direction of work. The same markers and color codes are valid for the rest of the figures.

Seven non-Levallois points were identified as hafted butchering knives. Three were typologically classified as notches and one as a convex side scraper (Table 3). One of the notched points, NMO13 3962, shows explicit evidence of hafting-related morphological adjustments, consisting of two notches located at the same height on the mesial part of both lateral edges. Butchery traces identified on the distal portion of the same edges indicate its use as a hafted butchering knife (Fig. S6). The presence of these two intentional notches indirectly demonstrates the use of bindings to secure the tool against the handle. The best example of a hafted skinning knife at NMO comes from the non-Levallois pointed element NMO14 4307 (Fig. 3). The use-wear traces consist of a polish with a rough texture and dull appearance, along with heavy edge rounding (Fig. 3a). The well-developed hafting polish on the dorsal ridges and the well-developed use-wear traces observed on the distal extremities of the edges suggest a long use duration. Another elongated non-Levallois point was identified as a hafted plant-cutting knife (Fig. S7). The wear traces consist of smooth polish with domed-flat topography and feather-terminated scars distributed along the distal part of the edge. Hafting traces were observed on the mesial and proximal portions of the artifact, consisting of bright spots, scarring, and polish (Fig. S7b–d).

In summary, the majority of the pointed elements in the sample (n = 15, 79%) were used for processing animal carcasses, and not in boring and plant processing, which were only observed on two artifacts (11%). Twelve of the pointed items (63%) were used as butchering knives, including a hafted skinning knife. Hafting was identified on 13 artifacts (68%), most of which are associated with activities for which hafting is not requisite, such as butchery (n = 7), dry-hide scraping (n = 1), and plant processing (n = 1). Two of the remaining hafted points are projectiles, and for two items the function was unidentified.

#### Cutting elements

Alongside the pointed elements, the NMO lithic assemblage contains a large number of artifacts with a significant edge suitable for cutting. In traditional typology, such items are not grouped together but their presence at NMO is important^66^. Of the cutting elements bearing use marks (n = 53, 61%), the typological categories with the highest frequency are naturally backed knives (n = 18), unretouched flakes (n = 17), and retouched flakes (n = 6) (Table 3). Out of 18 NBKs in the sample, 16 (89%) were observed to have use-wear indicating animal processing activities similar to the pattern observed in 21 of the 23 unretouched and retouched flakes showing use-wear (91%).

#### Naturally backed knives (NBKs)

Seven NBKs exhibit use-wear indicative of their use as butchering knives (39%), including two that were hafted. On one of these NBKs (NMO14 4361), the hafted part was identified on the distal extremity, where the cortical back is located (Fig. S8), indicating that the artifact was hafted and used with a proximal–distal orientation. Hafting traces consist of trapezoidal scars with step termination on the dorsal distal left edge (Fig. S8B), as well as hafting polish and micro-scars on the mesial dorsal left edge (Fig. S8C,D). The hafting scars indicate that the artifact was subjected to a high-pressure activity. These hafting traces are associated with notch retouch located at the same height on both lateral edges (Fig. S8A). This combination of features and the presence of retouch on the abrupt cortical back demonstrates that the artifact’s morphology was modified to fit a certain type of haft, which included the use of bindings to secure the artifact to its handle. Use-wear traces on the proximal portion of both lateral edges are composed of invasive polish and scarring indicative of butchery (Fig. S8F,G). Use-wear identified on another NBK (NMO11 2936) shows that the single edge of the artifact was used for both butchery and bone-scraping (Fig. S9).

**Figure 4:**
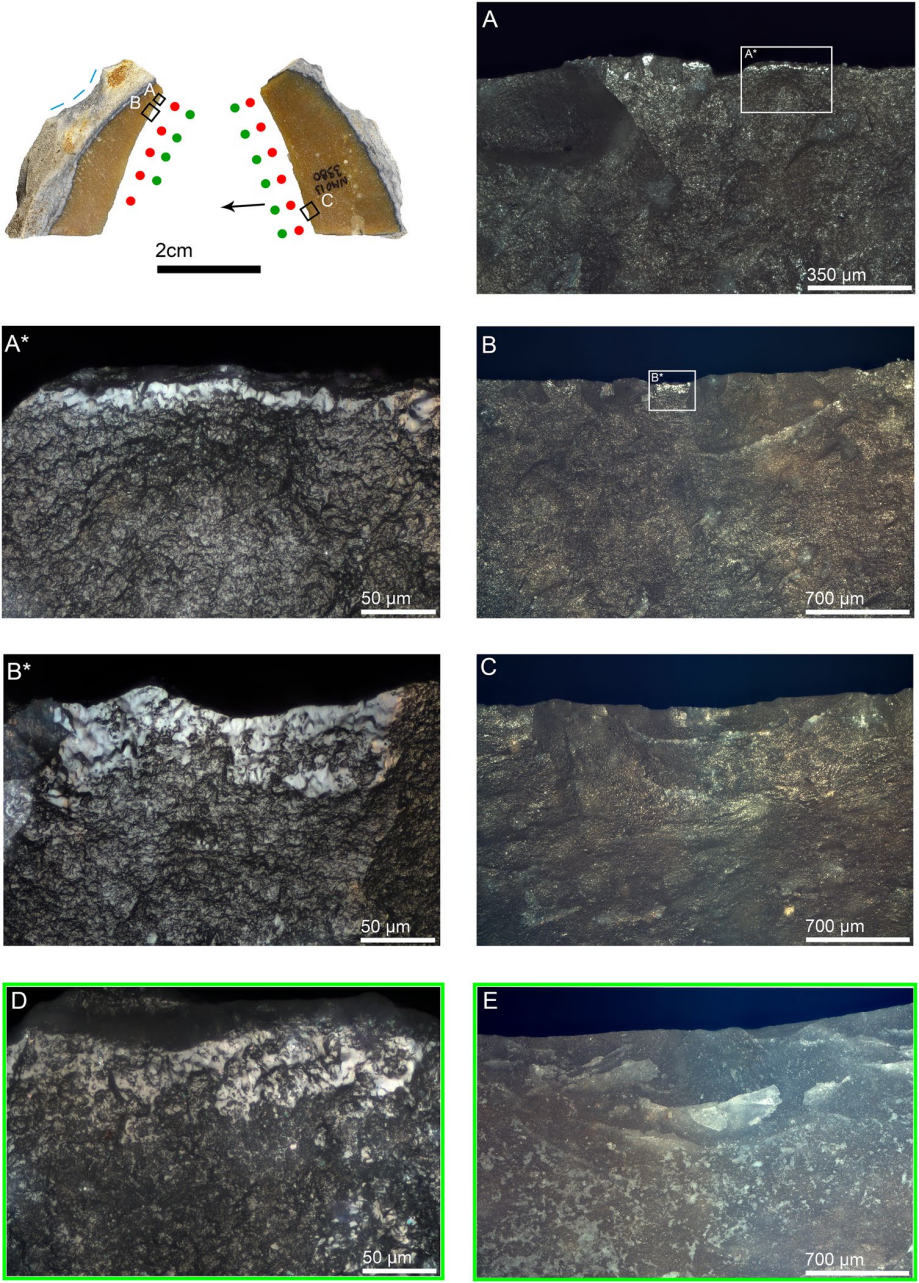
Naturally backed knife NMO13 3980 interpreted as an antler-scraping tool. (**A**) step-terminated macro and micro scars on the dorsal distal left edge with smooth polish restricted to the outer edge on elevated areas between scars, 100 ×; (A*) close-up of the previous picture in which the smooth texture and wavy topography of polish are appreciable, as well as transversal linear ripples indicating tool-use direction, 500 ×; (**B**) step-terminated trapezoidal scars associated with polish on the elevated areas between scars on the dorsal distal left edge, 50 ×; (B*) close-up of the previous picture showing well-developed polish with a smooth texture and wavy topography, 500 ×; (**C**) overlapped scalar scars showing fissured terminations on the ventral proximal right edge, 50 ×; (**D**) smooth-wavy polish on the mesial dorsal right edge of the experimental tool NMO-EXP50-A, used for scraping dry roe deer antler, 860 strokes, 10 min, 500 ×; (**E**) same experimental tool showing overlapped scalar scars with fissured terminations on the ventral distal right edge, 50 ×. The blue dashed line on the dorsal cortical back of the tool indicates retouch.

NBKs also account for the highest frequency of artifacts in the entire sample bearing evidence of bone/antler scraping (n = 5). The most explicit evidence was observed on a small NBK (NMO13 3980) retouched on the distal cortical back. Use-wear traces on the right edge indicate that the retouch was made to facilitate the grip of the artifact during use (Fig. 4). The observed use-wear consisted of smooth polish with wavy topography, located on elevated areas between scars (Fig. 4B,B*). Identical polish characteristics were seen on our experimental artifacts used for scraping dry antler (Fig. 4D).

**Figure 5:**
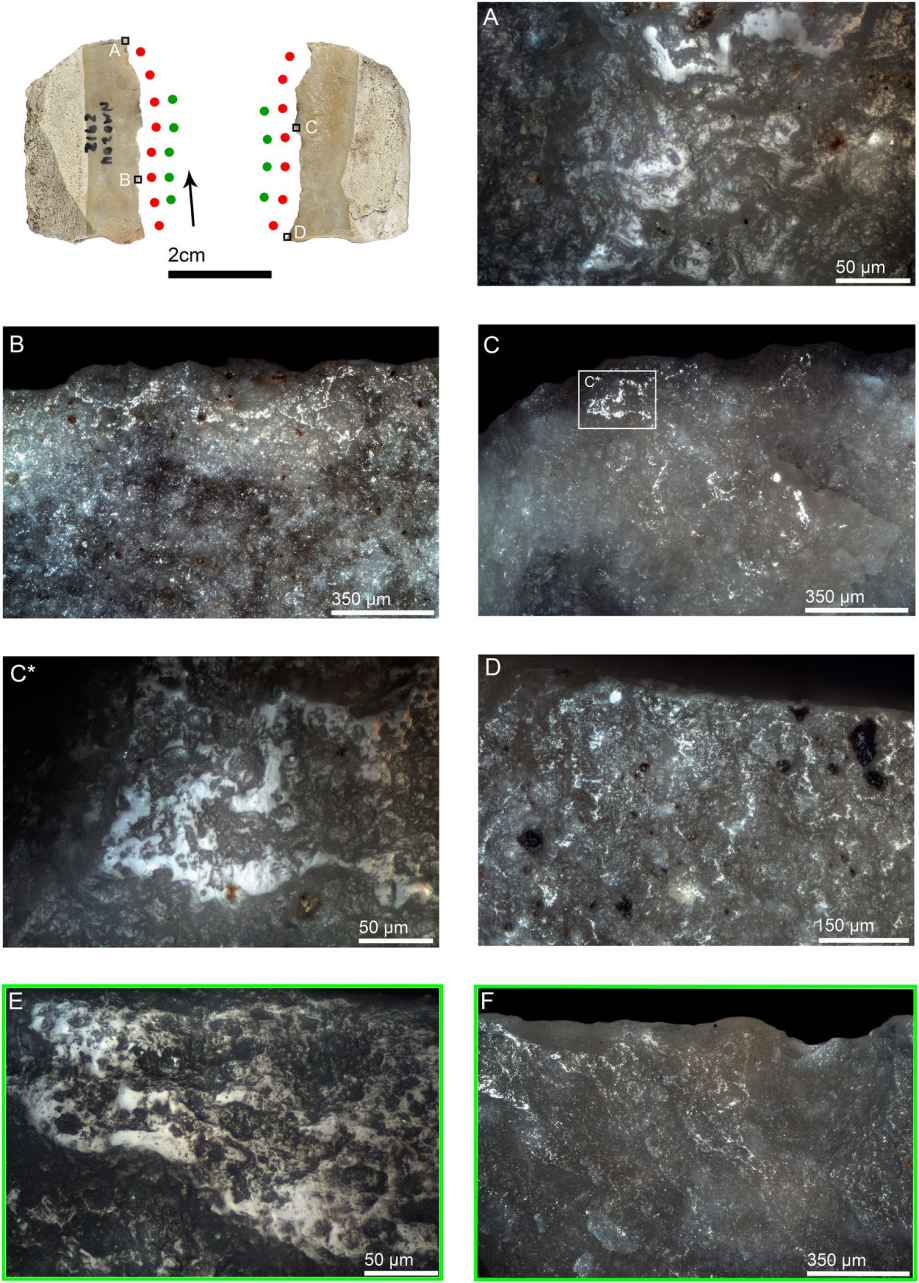
Naturally backed knife NMO11 2912 interpreted as a dry hide-cutting tool. (**A**) smooth polish associated with rough-dull polish and parallel striations on the dorsal distal right edge, 500 ×; (**B**) scalar scars with feather termination and polish on the dorsal proximal right edge, 100 ×; (**C**) scalar scars with feather termination and invasive polish on a protruding part of the ventral distal right edge, 100 ×; (C*) close-up of the previous picture showing the smooth texture and micro-wavy topography of well-developed polish, 500 ×; (**D**) invasive polish on the ventral proximal right edge, 200 ×; (**E**) well-developed smooth polish showing micro-wavy topography and pits on the ventral distal left edge of the experimental tool EXP-NMO25-P, used for cutting dry hide, 3172 strokes, 45 min, 500 ×; (**F**) scalar scars with feather termination associated with invasive polish on the ventral distal left edge of experimental tool EXP-NMO17-P, used for cutting dry hide, 2720 strokes, 40 min, 100 ×.

**Figure 6:**
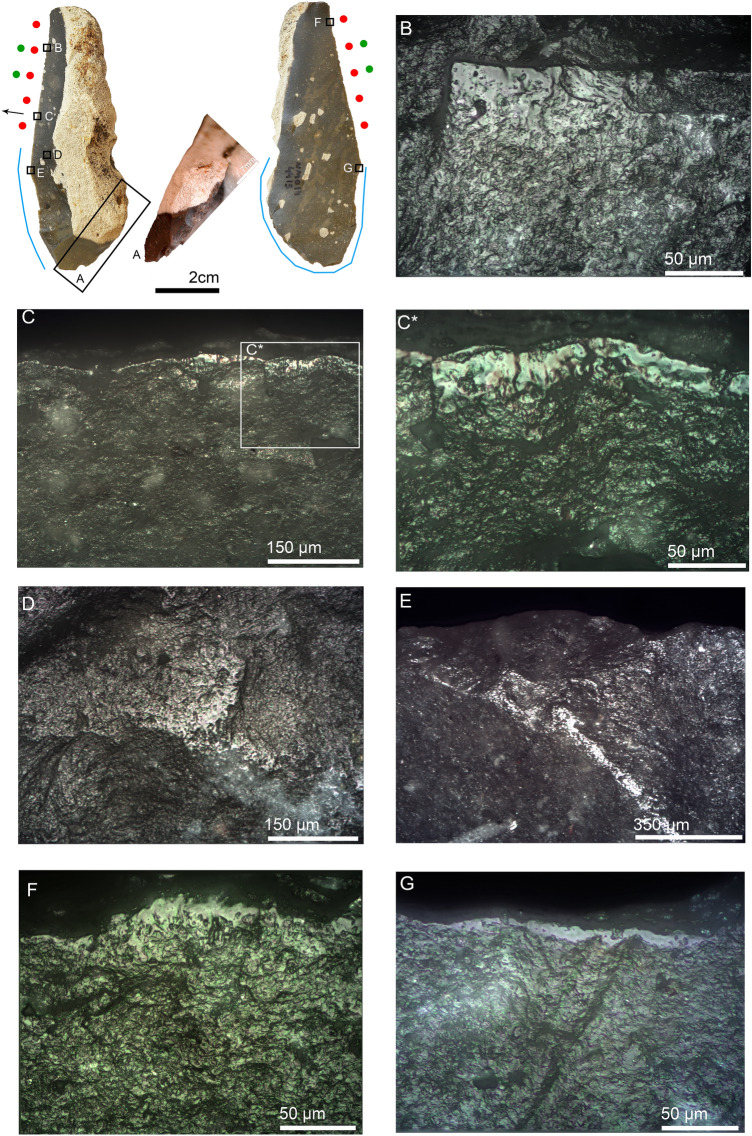
Naturally backed knife NMO14 4415 interpreted as a hafted wood-whittling knife. (**A**) abrupt retouch on the distal left edge, 10 ×; (**B**) smooth polish and transversal ripple on an elevated area between scars on the right proximal dorsal edge, 500 ×; (**C**) smooth-wavy polish and transversal ripples on the dorsal mesial right edge, 200 ×; (C*) close-up of the previous polish in which the smooth texture and wavy topography are appreciable, as well as transversal ripples indicating tool-use direction, 500 ×; (**D**) hafting polish on the mesial dorsal surface, 500 ×; (**E**) hafting scarring and hafting polish on the dorsal mesial ridge, 100 ×; (**F**) smooth polish with flat-wavy topography and transversal ripples on the ventral proximal right edge, 500 ×; (**G**) hafting bright spot on the ventral mesial right edge, 500 ×. *The tool is depicted upside-down to facilitate understanding of hafting.

Use-wear traces indicative of hide-processing were identified on four NBKs, two of which are small flakes used for cutting fresh (Fig. S10) and dry hide (Fig. 5). Particularly noteworthy is specimen NMO11 2912, which has an extremely developed polish associated with feather terminated scars (Fig. 5C,C*). A similar degree of polish development was achieved on several experimental replicas used for cutting dry hide (Fig. 5E). A single large NBK (NMO14 4415) displays clear signs of being used as a hafted wood-whittling knife (Fig. 6). Well-developed hafting polish on the mesial dorsal ridges (Fig. 6E) and hafting bright spots on the mesial ventral right edge indicate haft boundary (Fig. 6G). Abrupt retouch on the artifact clearly indicates that the original morphology was shaped to facilitate hafting (Fig. 6A), and well-developed, smooth polish on both sides of the mesial and proximal extremities of the right edge indicates wood-whittling (Figs. 6B,C,F, S11), though antler processing cannot be ruled out entirely. The well-developed hafting traces indicate a long use duration for artifact NMO14 4415.

#### Flakes, denticulates, and blades

Butchery is the most frequent activity observed on retouched (n = 3) and unretouched flakes (n = 11) (Table 3). However, hafting traces were only observed on one unretouched flake (NMO08 2052). Use-wear traces observed on unretouched flakes indicate hide-cutting (n = 3) (Fig. S12), bone-scraping (n = 1), and wood-whittling (n = 1). Two Levallois flakes were found exhibiting use-wear characteristic of hide-cutting and wood-whittling (Fig. S13) and four denticulates were observed bearing traces typical of butchery (n = 1), hide-cutting (n = 2), and plant processing (n = 1). Use-wear on denticulates is located on elevated areas of topography between retouch removals (Fig. S14). Use-wear on retouched blades indicate their use as butchering knives (n = 3), two of them while hafted. On one unretouched blade (NMO08 2122), well developed polish indicates whittling wood/plants (Fig. S15).

In sum, animal carcass processing is represented by 81% of the NMO cutting elements (n = 43), while wood and plant processing are observed on only seven artifacts (13%). Within carcass processing, butchery activity is most frequent, observed on 47% of the cutting items (n = 25), followed by hide-processing (n = 10, 19%), and bone/antler processing (n = 8, 15%).

## Discussion

The use-wear analysis of the NMO flint tools provided exceptional results. The unique nature of the assemblage comprising a relatively small number of artifacts, most of them in excellent preservation condition, allowed comprehensive study resulting in resolution rarely achieved for other Late MP assemblages. The use-wear analysis results demonstrate prominent focus (81%, n = 58) on animal tissue processing activities for all lithic typological groups (Table 3). These observations support the interpretation of NMO as a task-specific location^66^. Additional support is found in the exceptionally low percentage of artifacts displaying traces of wood/plant processing (n = 8, 11%). Butchery, rather than hunting, was the primary activity carried out on-site. This is especially clear for the pointed elements, for which use-wear traces reveal that 63% of the items (n = 12) were used as butchering knives, a higher frequency than that observed on cutting elements (n = 25, 47%). Differences also exist between pointed and cutting elements regarding wood/plant (5% vs. 13%), bone/antler (5% vs. 15%), and hide processing activities (5% vs. 19%). These percentages reveal that pointed elements at NMO were specialized tools used primarily as butchering knives and only rarely for other tasks. Cutting elements are less specialized and were used for additional tasks on different materials (e.g., bone-scraping, Fig. S16).

Similar results were obtained at the Levantine MP open-air site of Umm el Tlel, where 87% of the analyzed pointed items were used as butchering knives, and only 10% for plant processing^21^. Therefore, in contrast to what has often been proposed for Levallois points in Levantine caves and rock shelters^15,16,26,29^, our findings for NMO pointed elements—including Levallois and non-Levallois points—are in agreement with studies advocating a multi-purpose function of pointed items^27^, but with a particular focus on butchery^21^. The presence of lithic hunting weapons at NMO is rare, given the small number of projectile weapons revealed in this and other studies^76^. This is in agreement with the scarce evidence of projectiles reported for other MP open-air sites^22,59,60^.

One of the primary results of this study is the identification of both direct and indirect evidence for hafting on a substantial number of the items studied. Interestingly, pointed elements show higher frequencies of hafting traces (n = 13, 68%) than cutting elements (n = 9, 17%). This offers additional, strong support for the interpretation of NMO pointed items as specialized butchering tools. This interpretation is consistent with refitting and raw material import strategy studies^66^ indicating that Levallois and non-Levallois points were not produced in-situ but imported to NMO as part of the personal gear carried by hunter-gatherers.

NMO hafted pointed knives primarily have elongated proportions suggesting a correlation between hafting and elongation. Elongated, pointed morphology may have been a morphological requirement sought by its users for hafting. However, at NMO, this correlation between hafting and elongation is not only observed on pointed butchering knives. Variations in elongation index between all hafted and unhafted artifacts indicate that when hafting was required, elongated blanks (mostly points and blades) were favored over non-elongated items (flakes) for all activities (Fig. S17). This demonstrates that NMO tool-makers were well aware of the benefits that a handle can provide to an elongated blank, especially for butchering.

The identification of hafted butchering knives at NMO is remarkable. In the Levantine MP similar finds were only briefly noted in prior use-wear studies using low magnifications^16^. Hafted butchering knives have only been reported in a few Middle Stone Age (MSA) assemblages in Africa^40^ and MP sites in Europe^39,79^. For the latter sites, the frequency of the tasks and the need for increased pressure and longer artifacts for particular phases of the butchering process have been proposed as the primary reasons for the presence of hafted knives in addition to handheld ones^39,79^. At NMO, the majority of hafted butchering knives are pointed, whereas unhafted items bearing butchery traces are more frequent on non-pointed cutting items like flakes or NBKs. The occurrence for both hafted and unhafted butchering knives at NMO is not fully understood. One possible explanation is that hafted pointed knives provided higher precision and dexterity for specific butchering tasks, such as eviscerating and dismembering freshwater turtles and tortoises^72^. The frequency of tasks does not seem the most plausible reason behind the decision to employ hafted knives at NMO, given the limited number of hafted artifacts in comparison with unhafted ones. It is suggested that the need for increased pressure, precision, and dexterity during certain butchering processes was the driving force behind the decision to haft NMO knives.

Unhafted butchering knives, which make up the majority of non-pointed cutting elements, such as unretouched flakes, denticulates, or NBK, may have been used primarily for large game processing, for which precision and dexterity may have been less important. This demonstrates that the non-Levallois blade core reduction method at NMO was applied in order to obtain quickly cutting elements with long and sharp edges suitable for large game processing. In addition, scraping of large bones, probably of ungulates, was also performed on site, using large unretouched blanks such as NBKs (e.g., Fig. S16). The small NBK used for antler scraping (Fig. 4), may suggest that at NMO animal materials were also used for repairing or handle making.

Evidence observed of traces typical of hide and especially dry hide processing, even if limited, suggests that initial preparation of hides was performed at NMO. In low moisture conditions, the drying process of skins begins immediately after skinning, but full drying may take several days without human intervention^80^. It can be suggested that the NMO hunters dried and processed the skins of hunted animals immediately after the hunt. This practice was observed for modern hunter-gatherers such as the Tahltan, a semi-nomadic tribe of the Athapaskan Indians, where skins of hunted animals were dried and processed by women in hunting camps^8^.

Another activity identified at NMO is wood/plant processing, for which there is scant evidence, particularly among pointed items. This limited presence of vegetal processing activities is consistent with the interpretation of the site as a task-specific butchery location. Wood/plant processing at the site could have involved manufacture and maintenance tasks, such as the repairing of handles. Similar interpretations were proposed for European MP sites where limited evidence of wood processing tools attests to repairing activities in preparation for hunting events^39^. Preparation of vegetal structures (wrappers and containers) for packaging and transporting of meat from riparian plants typical of the Hula Valley^81^ may offer an additional explanation for the presence of such use-wear traces at NMO. Similar behaviors were noted in Australian aboriginal hunter-gatherers, who used several bark trees and plants leaves in the making of food wrappers and containers^82^.

Of particular significance is the identification of explicit hafting wear on flint artifacts for which neither their morphological attributes nor the activities for which they were used dictate—in our modern eyes—insertion into a handle. This applies not only to the pointed butchering knives previously described, but also to a limited number of cutting elements, such as NBKs (n = 4), retouched blades (n = 2), unretouched and retouched flakes (n = 2), and a typical end-scraper (Table 3). Particularly important is the identification of hafting traces in close association with morphological adaptations (distal thinning, notching), indicating that NMO hunter-gatherers had access to highly sophisticated hafting expertise.

This expertise is evident for the two large NBKs identified as a hafted butchering knife (Fig. S8) and a hafted wood-whittling tool (Fig. 6), respectively. In both cases, highly developed hafting traces are closely associated with morphological adaptations and well-developed use-wear traces. The characteristics and distribution of hafting wear suggest that both artifacts were inserted in male arrangements^33,83,84^ using animal hafts. Using animal resources as handles (e.g., bone, antler or horn) usually necessitates morphological adaptations to the stone tools^33,35–38^. Such morphological adaptations are not systematically present in the entire set of hafted artifacts identified in this study. This means that wooden handles or other plant resources would have been preferred as haft materials at NMO, possibly as part of male split arrangements (e.g., Fig. S7). NMO inhabitants had access to a variety of plant resources around Paleo Hula Lake^81^, thus reducing the likelihood that ecological constraints influenced their decision to use animal resources as handles. In contrast, it seems that such a decision would have been dictated by the size and morphology of NBKs, for which animal handles would have been more suitable. It is not unreasonable to think that both NBKs were designed and knapped as part of the personal toolkit of hunter-gatherers. The highly developed hafting and use-wear traces found on both artifacts suggest that these NBKs were likely used for long periods. In one of these NBKs (Fig. S8), use-wear does not match the intensity of hafting wear, which are extremely developed, suggesting that resharpening may have removed previous use-wear. The hafted NBK used for wood-whittling demonstrates that mobile tools designed and prepared for maintenance tasks were transported by hunter-gatherers during the Late MP. The small size of the other wood-whittling tools (e.g., Fig. S13) and the fact that they were likely handheld may suggest that the large hafted NBK used for wood-whittling was brought to the site as part of a personal toolkit.

Hafted NBKs in Levantine MP assemblages have not been reported previously, which demonstrates the NMO uniqueness in this regard. Evidence of hafted artifacts used in activities for which hafting is, theoretically, not required, such as butchery, wood or plant processing, has been reported in other MP assemblages. Hafted artifacts used for wood and plant processing were observed on Early MP Abu Sif points from Levantine MP caves^26^. In Europe, several types of MP scrapers, flakes, and points show similar evidence^39,79^. Accordingly, the large hafted NMO NBKs attested to in this study are, to our knowledge, one of the first examples of hafted NBKs found in MP assemblages. This suggests that, in addition to artifact function, hafting decisions for NMO stone tools may have been influenced by lithic tradition and personal choices.

Spatial plotting of the artifacts bearing use-wear traces from excavation Area D at NMO did not provide clear patterns identifying specific activity areas (Fig. S18). The pieces bearing traces of use are located in the areas with highest density of archaeological material where no accumulation pattern was discerned, neither by type of activity, nor by material or action. This absence of ability to reconstruct activity areas considered in conjunction with the specialized nature of the assemblage offers additional support to the interpretation of NMO as a short-term task-specific locality. In such a locality, no clear activity areas are expected, as no repeated intensive activity will have taken place. The mint preservation condition of flint tools and the bones at the site also suggest fast covering in which artifacts were not exposed to atmospheric conditions. In other words, the assemblage of Layer 4b documents activity in close proximity to or even in the mud forming the shore of the Paleo Hula Lake. These conditions would have prevented the creation of distinct activity areas associated with long duration activity.

## Conclusions

NMO is a unique short-term kill/butchery locality dated to the Late MP of the Levant. The small lithic assemblage, with a high percentage of pointed and cutting elements, offered an opportunity to study a task-specific assemblage, free of “background noise” typical of the long sequences of large cave sites of the Levant. Application of use-wear study to this assemblage yielded exceptional results enabling an in-depth understanding of the technology and activity patterns executed at NMO. The fact that flint sources are not available in the vicinity of NMO^66^, together with the small number of artifacts in the flint assemblage and the high frequency of retouched items, facilitated recognition of the provisioning of individuals strategy^56^. Combining the use-wear study results with an understanding of the flint economy, technological patterns, and refitting studies enabled the reconstruction of the following patterns at NMO: Well-made elongated points (Levallois and mostly non-Levallois) as well as some elongated cutting items were imported to the site as part of the hunter’s personal toolkit. The primary activity for which these items were used was butchery, for which hafting was crucial. The limited number of projectiles abandoned at the site can be explained by the functionality of NMO as a prey processing site, rather than with the absolute number of weapons in the toolkits. Projectiles may have just passed throught the site if no retooling took place for their part. Cutting elements (blades and flakes) were produced on-site using flint nodules that were transported to the site using a simple but highly efficient knapping method^66^, anticipated by a provisioning of places strategy. The use-wear analysis results demonstrate that these expedient cutting tools were used for a variety of activities, primarily large game processing, but also hide-processing, bone-scraping, and wood/plant processing. In addition, a few expedient cutting elements were hafted and brought to the site with the personal gear, primarily for use in butchering and maintenance tasks.

The results of this study, obtained through systematic low-power and high-power magnifications, place NMO as a unique case study for the understanding of the Late Levantine MP. The mint condition of flint tools enabled identification of well-developed use-wear and hafting traces. The significant number of hafted artifacts attested to in this study, particularly in the shaping of pointed items, indicates a high level of planning and anticipation of activities at NMO. NMO inhabitants possessed hafting expertise, as evidenced by handle making for stone tools used in tasks for which hafting is not requisite. It also validates the interpretative potential of hafting when systematically integrated with use-wear and technological data to better understand past human behavior and subsistence strategies during the Middle Paleolithic.

## Materials and methods

### Sampling

A two-step screening procedure was applied to the entire lithic assemblage of NMO, focusing on the in-situ assemblage of Layer 4b, to select the most suitable artifacts for use-wear analysis (Table 1). In the first step, the whole assemblage was screened using the naked eye and a hand magnifying glass. This phase aimed to assess the general state of surface preservation of the artifacts to identify potentially used areas and macro-scars visible with the naked eye. As a result of this screening, 332 artifacts were selected as having good potential for further screening, based on their state of surface preservation and the presence of potentially used edges. Selected artifacts went through a second screening procedure in which previous naked-eye observations were crosschecked against low-power observations using a Motic zoom stereo microscope. A final sample of 129 artifacts (Table 2) was selected during this second stage as bearing potential use-scars or fresh surfaces suitable for detailed low and high-power analysis. At all times, artifacts were handled using free-powdered nitrile gloves to avoid contamination of stone surfaces with hand grease or modern particles from the laboratory^85^. Stone tool surfaces were not cleaned at this stage, and residues were not the focus of this investigation due to earlier handling of the material during excavation. Nonetheless, when it was anticipated that residue remains would provide useful information for the tool’s functional interpretation, they were noted.

### Experimental reference collection

Archaeological wear interpretations rely on comparisons with the large and systematic experimental reference collection available at IPHES, including a collection begun in previous publications^86,87^. In addition, a large reference collection was specifically designed with the aim of interpreting the archaeological wear of NMO. This reference collection is made up of 81 experimental tools knapped on flint collected in the south-west ridge overlooking the Hula Valley^88^. Tools were knapped by an experienced knapper (M. Guardiola) at IPHES in an attempt to replicate the NMO artifacts. Stone tool replicas were then used in experimental activities by one of the authors (JIMV), who has more than eight years of experience in using handheld and hafted tools and who has received training from professional butchers in a cutting room. One of the co-authors (AO), who has approximately thirty years of experience using hand-held and hafted tools, provided advice and materials for the execution of some experiments. Experiments involved the processing of animal tissue, plant, and inorganic materials (Figs. S19–S21, Table S1). Additional experiments aimed at recognizing production (Table S2) and hafting wear (Table S3) were performed (Figs. S22 and S23). The hafting wear experiments (n = 5) included male split hafting arrangements, as well as male notched hafting arrangements with and without the application of bindings. Lastly, the collection includes a limited set of sequential experiments designed to monitor the use-wear development on different materials over different time intervals. This experimental activity involved a total of 106 individual experiments. Additional support for archaeological wear interpretations relied on previously published studies^33,77,89–94^.

### Analytical procedure

Wear traces on archaeological and experimental tools were recorded following a multi-analytical and multi-scalar approach^87^, which relies on the combination of optical, 3D digital, and scanning electron microscopy (Table S6). Archaeological tools were examined at the lithic laboratory of IPHES first using a 3D digital microscope Hirox KH-8700 for low-power observations (magnifications ranging from 35 × to 140 ×). Lateral lighting was used to record all the macro-scars visible on the edges to describe their initiation, termination, morphology, orientation, and distribution^95,96^. High-power observations were conducted with a metallurgical microscope Zeiss Axio Scope A1 using incident lighting (magnifications 50 ×–500 ×), which enabled the observation of polish, edge rounding, striations, and micro-scarring. Use polish, edge rounding, and striations were described according to the principles outlined in several publications^92,97,98^, and hafting traces were interpreted following guidelines stated by Rots^33,84^. Finally, when necessary, a Fei Quanta 600 scanning electron microscope was used in low-vacuum mode (35 ×–2000 ×) to supplement and assist optical identification of wear traces, primarily polish and striations.

### Cleaning protocols

Archaeological and experimental tools were cleaned following protocols published in previous studies^86,99^. For archaeological tools, the protocol consisted of a 15-min ultrasonic bath in a neutral soap solution (2% Derquim ® LM02), followed by an ultrasonic bath in water for 10 s, and a final ultrasonic bath in pure acetone for 5 min. After the conclusion of the experiments, experimental tools were submerged in 30% hydrogen peroxide for 2 to 24 h to soften any adhering organic residues. After the large masses of residues had softened sufficiently, the tools were subjected to an ultrasonic bath of hydrogen peroxide (30%) for 15 min, followed by a neutral soap solution ultrasonic bath (2% Derquim ® LM02) for 15 min, water for 10 s, and a final ultrasonic bath of pure acetone for 5 min.

## Supplementary Information

Supplementary Information.

## Data Availability

All data generated or analysed during this study are included here and in the supplementary information.
