# Supplementary material for: Butchering knives and hafting at the Late Middle Paleolithic open-air site of Nahal Mahanayeem Outlet (NMO), Israel
Source: Sci Rep. 2023 Jan 3;13:112. doi: 10.1038/s41598-022-27321-5 (PMC9810700; doi:10.1038/s41598-022-27321-5)
Supplement: Supplementary file 1 — Supplementary Information. [file 41598_2022_27321_MOESM1_ESM.pdf]

Butchering Knives and hafting at the Late Middle Paleolithic open-air site of Nahal Mahanayeem Outlet (NMO), Israel

Supplementary Information

Juan Ignacio Martin-Viveros[1,2*], **Maya Oron**[3,4], **Andreu Ollé**[1,2], **M. Gema Chacón**[1,2,5], **Gonen Sharon**[6]

[1]Institut Català de Paleoecologia Humana i Evolució Social (IPHES-CERCA), Zona educacional 4,

Campus Sescelades URV (Edifici W3), 43007 Tarragona, Spain

[2]Departament d'Història i Història de l'Art, Universitat Rovira i Virgili (URV), Avinguda Catalunya 35,

43002, Tarragona, Spain.

[3]Archaeological Research Department, POB 586, Jerusalem 91004, Israel Antiquities Authority

[4]Institute of Archaeology, The Hebrew University of Jerusalem, Mt. Scopus, 91905

[5]UMR7194 – HNHP (CNRS – MNHN –UPVD – Sorbonne Universités), 1 rue Renè Panhard, 75013 París, France, Musée de l'Homme, 17 Place du Trocadéro, 75016 París, France

[6] Department of Galilee Studies (M.A.), Tel-Hai College, Upper Galilee, Israel

*Corresponding author, email: ignacioj446.jm@gmail.com

1. THE NMO SITE

1.1 Evidence for short term occupation of the site and fast burial of the remains

The evidence for the short term and fast covering nature of the archaeological horizon Layer 4b are as follows:

a. The archaeological finds were found in fine mud. There is no evidence of layering or other processes indicating slow formation, and sedimentological observation suggests a rapid accumulation rate for this layer [1].

b. The excellent preservation of the bones and the mint condition of flint tools at the site. It is evident that none of these finds were exposed to atmospheric conditions for a long period or even a few months [1,4].

c. The preservation of botanical remains at the site provides further evidence. Preservation of botanical remains due to the waterlogged condition of the soils is well-known at the prehistoric sites on the banks of the Jordan River in this vicinity [2,3]. A large number of wood remains, as well as fruits and seeds, were recovered from the sediments of Layer 4. The actual presence of botanical remains within the layer indicates that they could not have been exposed on the surface for a long period of time.

d. Further evidence for the short duration of the occupation can be seen in the presence of a significant number of refitted artifacts scattered throughout the site. These artifacts resulted from at least 3 reduction sequences connecting the different sections of the site into a single event [4]. It is suggested that at least some of the artifacts in the archaeological layers were discarded by the site inhabitants directly into the mud or shallow water of the lake surrounding the basalt hill of Layer 5.

e. The stone tool assemblage of the site also strengthens the argument for a short occupation. The number of artifacts excavated per excavation unit at the site is exceptionally low when compared to any other MP site in the Levant. At most cave sites, the density of the finds is extremely high, but the NMO density is very low even when compared to other open-air sites in the Levant [5,6]. The low number of NMO stone tools clearly indicates a low intensity of site occupation and makes it highly unlikely that it represents an area of long-term activity.

1.2 Flint assemblage

The flint assemblage from NMO area D is small, counting 2195 items of debitage, 648 retouched items, and 8519 pieces of debris (mostly chips) from an excavated area of ~80m². Of these, 80% come from layer 4b, seen as a well-preserved archaeological horizon above the basalt pebble layer. Some very well-preserved items collected from the surface around the excavation area most likely originated from the layer and were removed by modern activity along the Jorden River, and were carefully integrated into some of the analysis.

The presence of Levallois products and some typological groups represented in the assemblage clearly relate it to known assemblages from the Middle Palaeolithic of the Levant, but it is characterized by some unique attributes that probably resulted from the short-term and task-specific nature of the occupation. The retouched item group is dominated by expedient tool types such as non-Levallois retouched items, notches, and denticulated items, followed by a significant group of knives and points. The dominance of knives and points is enforced when looking at non-retouched artifacts, where items with long cutting edges and pointed elements are dominant [4]. Some of the items, especially points and pointed items, were clearly introduced into the site as finished tools. Other tools, such as unretouched knives, flakes, and blades, were produced on-site using raw material brought into the site for expedient knapping. Technologically, the assemblage is dominated by non-Levallois reduction sequences, mostly production of flakes,

points, and blades from single platform cores, and flake production from cores on flake. Flint is not available close to the site. 49 flint artifacts from Layer 4b were refitted into 10 sequences of 2 to 20 pieces each and seem to represent three to five reduction sequences. All refitted sequences are part of non-Levallois single platform or short core on flake flaking systems.

2 RESULTS

2.1 Artifact function: general overview

Observations regarding the morphology of the used edges reveal that straight edges were preferred for both cutting (n = 33) and scraping/whittling activities (n = 12). Used edges with sinuous and convex horizontal profiles were the second most frequent morphologies in cutting (n = 24) and scraping/whittling activities (n = 4), respectively. No significant difference was found between the used edge angles in cutting (mean 40º, n = 70) and scraping/whittling activities (mean 44º, n = 21).

Two general patterns of butchery wear were observed. The first pattern consisted of feather-terminated scalar and sliced scars, often associated with invasive rough polish due to contact with soft animal tissue (Fig. S8g). The second pattern was interpreted as caused by contact with hard animal tissues (e.g. bone, cartilage.) and consisted of large scalar scars, sometimes overlapped, showing crushed initiations and fissured terminations, and sometimes associated with smooth bone polish (Fig. S9a, b). Wear patterns on hide-processing tools consisted of feather terminated scalar scars with wide initiations associated with well-developed edge rounding and well-developed invasive rough polish, showing micro-pits and a dull appearance (Fig. S10a, b, c). Sometimes smooth polish surrounded by rough-dull polish and feather scalar scars is indicative of dry-hide processing (Fig. S12c). Bone/antler wear traces consisted of step-trapezoidal and semi-circular scars with wide and cone initiations, sometimes showing fissured terminations and crushed initiations, associated with smooth polish showing transversal ripples on the very edge (Fig. S16). Wood/plant wear patterns consisted of well-developed smooth polish showing doomed, but also wavy topography and associated with step-trapezoidal and semi-circular scars with few or no signs of crushing (Fig. S15).

Hafting traces consisted mainly of bright spots (Fig. S7b) or polish located on dorsal ridges and mesial and proximal portions of the edges (Fig. S8c). Macro-wear traces consisted of lateral scarring (Fig. S7c) or groupings of scalar scars showing feather or step terminations (Fig. S8b). Finally, indirect evidence of hafting, such as proximal or lateral retouch, or bilateral notching, were also recorded in a few cases in close association with macro and micro-wear hafting traces (Fig. S8a).

3. METHODS

3.1 Analytical procedure

To assess the reliability of interpretations, all archaeological pieces bearing use-wear traces were given a reliability index for each interpretative category (use motion, activity, worked material hardness, type of worked material, and presence of hafting traces) on a scale of 0 (uncertain interpretation), 1 (poor reliability), 2 (moderate reliability), 3 (high reliability), and 4 (reliable interpretation). A score was first assigned for individual used edges, so that tools showing multiple used edges have two values, one for each used edge. The mode was considered the final reliability index in the functional interpretation for each used edge in each tool.

Reliable interpretations were considered when all types of use-wear were found associated on the tools or, at least, when a minimum of two types of use-wear were identified indicating diagnostic features, preferably in association (e.g., polish and scars). Interpretation was also reliable when well-developed use-wear polish was not associated with scars or edge rounding, but instead displayed diagnostic features of specific contact materials and the preservation of the artifact was excellent (Fig. S16). Highly and moderately reliable interpretations were assigned when all or several types of wear were identified but showed

less development or lacked a complete set of diagnostic features. Interpretations with poor reliability include those cases in which wear traces showed poor development and did not show any diagnostic features, but they were suspected of being of use. Finally, uncertain interpretations were considered when functional interpretation could not be determined due to post-depositional alteration.

3.2 Microscopic analysis

We used a Zeiss Axio Scope A1 reflected light metallurgical microscope equipped with 10x oculars and four objectives: EC epiplan 5x/ 0.13 HD; EC epiplan 10x/0.2 HD; LD epiplan 20x/0.4 HD DIC; and LD epiplan 50x/0.5 HD DIC. All these objectives provide magnifications ranging from 50x to 500x. The images were taken with a 5MP Invenio 5SII digital camera using Aco-z software. To compensate for the short depth of field of the microscope, all images were stacked using Heliconfocus software. For image acquisition, we used an FEI Quanta 600 SEM model, equipped with an INCA suite software (v 4.01) from oxford instruments and energy dispersive X-ray spectrometer (EDX-EXL II System link analytical oxford) for microanalysis. All samples were observed in low vacuum mode using the secondary electron detector (Large field detector, LFD), so that metallization of samples was not required. Finally, we used a 3D Digital microscope, Hirox KH-8700, which is equipped with a dual illumination revolver zoom lens MX-5000REZ with a high-intensity LED light source. The color temperature of the sample images obtained (5700k) is very close to that of daylight (5300k). The revolver zoom lens consists of a triple objective turret, with each objective providing a specific range of magnifications: OL-35 low-range objective (35x – 250x), OL-140 II mid-range objective (140x – 1000x), and OL-700 II high-range objective (700x – 5000x). Coaxial and ring lighting illumination systems or a mixture of both can be chosen according to the observation conditions required by the characteristics of the sample. For acquisition and live image operation, the microscope is equipped with a full HD LCD monitor and CCD camera, which can capture 24 fps at high-quality resolution (1200 x 1600 pixels). A stitching technology is integrated into the microscope, which enables making 2D and 3D images of the sample surface. Therefore, wide field of view images can be obtained through this integrated technique.

References

1. Kalbe, J., Sharon, G., Porat, N., Zhang, C. & Mischke, S. Geological setting and age of the Middle Paleolithic site of Nahal Mahanayeem Outlet (Upper Jordan Valley, Israel). *Quat. Int.* **331**, 139–148 (2014).

2. Aharonovich, S., Sharon, G. & Weinstein-Evron, M. Palynological investigations at the middle Palaeolithic site of Nahal Mahanayeem Outlet, Israel. *Quat. Int.* **331**, 149–166 (2014).

3. Melamed, Y., Kislev, M. E., Geffen, E., Lev-Yadun, S. & Goren-Inbar, N. The plant component of an Acheulian diet at Gesher Benot Ya'aqov, Israel. *Proc. Natl. Acad. Sci. U. S. A.* **113**, 14674–14679 (2016).

4. Sharon, G. & Oron, M. The lithic tool arsenal of a Mousterian hunter. *Quat. Int.* **331**, 167–185 (2014).

5. Oron, M. & Goren-Inbar, N. Mousterian intra-site spatial patterning at quneitra, golan heights. *Quat. Int.* **331**, 186–202 (2014).

6. Centi, L. & Zaidner, Y. Variations in lithic artefact density as a tool for better understanding Middle Palaeolithic human behaviour: The case of Nesher Ramla (Israel). *Quat. Int.* **624**, 4–18 (2022).

Supplementary Tables

Tool ID	Blank	Tool Type	L x W x T	UEdge	LUEdge	HEpro	SEpro	Eangle	Activity	Wmat	Swmat	WAngle	WTime	Strokes
NMO-EXP01-C	flake	U. flake	56 x 31 x 8	right	48	st	inc	43	skinning	skin, meat	fresh	70 - 90º	120	5250
NMO-EXP02-C	flake	U. flake	40 x 30 x 7	right	26	sin	st	35	defleshing	meat, fat, tendon, bone	fresh	80 - 90	27	1350
NMO-EXP03-H	flake	U. flake	42 x 26 x 4	right	33	sin	inc	45	scraping	bone	fresh	50-60	60	3839
NMO-EXP04-H	flake	U. flake	35 x 34 x 6	right	30	st	sin	49	sawing	tendon, bone	fresh	90	35	5700
NMO-EXP05-H	flake	U. flake	43 x 35 x 6	left	25	st	inc	40	sawing	bone	dry	80 - 90º	40'	5806
NMO-EXP07-P	blade	U. blade	46 x 20 x 5	left	33	cx	inc (sin)	27	cutting	dry hide	dry	60-70º	5'	320
NMO-EXP09-V	flake	U. flake	26 x 22 x 4	right	26	sin	st	27	cutting	plants	fresh	70-80	20	1500
NMO-EXP10-V	blade	Single convex side scraper	60 x 30 x 11	right	56	sin	inc	35	sawing	plants	fresh	90º	22	1980
**NMO-EXP11-V**	flake	U. flake	41 x 34 x 6	left	32	sin	inc	33	cutting	plants	fresh	70 - 80º	30'	2155
				right	40	sin	st	31	cutting	plants	fresh	70 - 80º	60’	4272
NMO-EXP12-V	flake	U. flake	46 x 29 x 13	right	46	sin	rt	55	scraping	plants	fresh	60 - 70 º	35'	2000
NMO-EXP13-V	blade	U. blade	47 x 21 x 10	right	40	st	sin	43	whittling	plants	fresh	40 - 50º	30	2679
NMO-EXP14-P	flake	U. flake	28 x 27 x 8	right	23	sin	st	25	cutting	hide	fresh	70 - 90º	45'	2115
NMO-EXP15-C	point	Non-Levallois point	42 x 23 x 4	left	37	sin	st	38	defleshing	meat, fat, tendon, bone	fresh	70 - 90º	45'	1150
**NMO-EXP16-M**	flake	U. flake	58 x 30 x 8	left	45	st	st	47	shaving	wood	fresh	90º	30'	2124
				right	36	cc	st	50	shaving	wood	fresh	90º	5’	326
NMO-EXP17-P	flake	R. flake	54 x 37 x 11	left	46	sin	st	45	cutting	hide	dry	50º - 70ª	40'	2720
NMO-EXP18-C	blade	U. blade	44 x 21 x 6	left	39	sin	st	26	cutting	meat, fat, tendon	fresh	60 - 80º	40'	2490
NMO-EXP19-M	flake	U. flake	33 x 52 x 16	right	34	st	st	43	shaving	wood	fresh	90º	30'	1908
NMO-EXP22-H	flake	U. flake	66 x 43 x 13	left	55	st	inc	40	scraping	bone	fresh	50 - 70º	40'	2352
**NMO-EXP23-M**	point	Non-Levallois point	72 x 30 x 13	left	62	st	st	55	whittling	wood	fresh	30 - 50º	20'	800
				right	47	st	st	57	scraping	wood	fresh	60 - 80º	20’	1100

**NMO-EXP24-M**	blade	U. blade	68 x 24 x 10	left	60	st	inc	41	scraping	wood	semi-fresh	50 - 70º	40'	2552
				right	53	st	inc	43	scraping	wood	semi-fresh	90º	10'	758
**NMO-EXP25-P**	flake	Denticulate	67 x 37 x 21	left	41	dent	sin	55	cutting	hide	dry	70 - 80º	45'	3172
				right	51	dent	st (sin)	70	scraping	hide	dry	50 – 70º	45'	2817
NMO-EXP26-P	blade	U. blade	58 x 21 x 4	right	49	sin	inc	50	scraping	hide	fresh	70 - 80º	45'	3312
NMO-EXP27-M	blade	U. blade	51 x 20 x 12	right	45	st	inc	45	sawing	wood	fresh	90º	30'	5000
NMO-EXP28-M	blade	U. blade	73 x 29 x 10	left	65	st	st	50	sawing	wood	fresh	90º	20'	2852
**NMO-EXP29-M**	flake	U. flake	31 x 18 x 5	left	25	st	st	32	sawing	wood	fresh	90º	5'	466
				right	24	rt	rt	48	sawing	wood	fresh	90º	10'	1140
NMO-EXP30-M	blade	U. blade	57 x 20 x 10	right	47	cx (sin)	inc (st)	42	sawing	wood	dry	90º	20 '	3200
**NMO-EXP31-M**	flake	U. flake	40 x 22 x 4	left	27	st	st	45	sawing	wood	fresh	90º	5'	752
				right	33	st	inc	57	sawing	wood	fresh	90º	10'	1624
**NMO-EXP32-M**	flake	U. flake	28 x 19 x 5	left	25	st (cx)	st (inc)	49	sawing	wood	dry	90º	10'	1480
				right	25	st	st	57	sawing	wood	dry	90º	5’	600
NMO-EXP33-M	flake	R. flake	31 x 24 x 8	left	29	cx	st	36	sawing	wood	fresh	90º	20'	2632
**NMO-EXP34-M**	flake	U. flake	35 x 20 x 5	left	27	st (cc)	inc	60	shaving	wood	fresh	90º	10'	480
				right	29	st	inc	50	shaving	wood	fresh	90º	5'	262
**NMO-EXP35-M**	flake	U. flake	27 x 18 x 4	left	25	st	st	46	shaving	wood	fresh	90º	10'	490
				right	23	st	st	43	shaving	wood	fresh	90º	5'	326
NMO-EXP36-M	blade	U. blade	61 x 29 x 8	left	47	sin	st	42	shaving	wood	dry	90º	20'	1454
**NMO-EXP37-H**	flake	Notch	34 x 20 x 5	left	33	sin	inc	34	scraping	bone	fresh	50 - 70º	5'	312
				right	25	sin	sin	56	scraping	bone	fresh	50 – 70º	10'	660
NMO-EXP39-H	blade	U. blade	47 x 16 x 4	left	42	st (sin)	inc	45	scraping	bone	fresh	50 - 70º	20'	1800
NMO-EXP40-H	flake	End-scraper	25 x 26 x 6	left	22	st	st	70	scraping	bone	soaked	50 - 70º	10'	520

**NMO-EXP41-H**	blade	Notch	44 x 22 x 7	left	34	sin	inc	43	scraping	bone	fresh	90º	10'	740
				right	26	sin	inc	44	scraping	bone	fresh	90º	5'	280
**NMO-EXP42-H**	flake	U. flake	41 x 21 x 4	left	37	st	st	37	scraping	bone	fresh	90º	10'	867
				right	36	st	st	22	scraping	bone	fresh	90º	5'	463
NMO-EXP43-H	point	Non-Levallois point	50 x 21 x 5	left	46	st (sin)	inc	54	scraping	bone	fresh	90º	30'	2200
NMO-EXP44-A	point	Non-Levallois point	33 x 15 x 4	right	32	cx	st	46	sawing	antler	dry	90º	3'	214
NMO-EXP45-H	flake	U. flake	32 x 20 x 6	left	29	cx (st)	st	54	scraping	bone	fresh	50 - 70º	20'	1250
NMO-EXP46-H	flake	U. flake	23 x 23 x 3	right	25	st	st	50	sawing	bone	fresh	90º	20'	2810
NMO-EXP47-H	blade	U. blade	36 x 15 x 3	right	35	st	st	45	sawing	bone	dry	90º	10'	1665
**NMO-EXP48-A**	point	Non-Levallois point	33 x 17 x 4	left	29	sin	st	29	sawing	antler	dry	90º	5'	464
				right	23	rt	rt	50	sawing	antler	dry	90º	10'	1300
**NMO-EXP49-A**	flake	U. flake	22 x 18 x 4	left	20	st	st	20	sawing	antler	soaked	90º	10'	1400
				right	18	rt	rt	40	sawing	antler	soaked	90º	5'	577
NMO-EXP50-A	flake	U. flake	28 x 19 x 4	right	27	st (cc)	sin	57	scraping	antler	dry	50 - 70 º	10'	860
**NMO-EXP51-A**	blade	U. blade	33 x 15 x 4	left	30	st	st	50	scraping	antler	dry	90º	5'	450
				right	32	st (sin)	st	40	scraping	antler	dry	90º	10'	711
NMO-EXP52-H	blade	Side-scraper	53 x 21 x 10	right	45	sin	inc	52	scraping	bone	fresh	50 - 70 º	10'	500
NMO-EXP53-H	point	Non-Levallois point	38 x 17 x 4	right	35	st	st	38	scraping	bone	soaked	50 - 70º	20'	840
NMO-EXP54-H	flake	Notch	39 x 29 x 10	left	28	sin	inc	40	scraping	bone	dry	50 - 70º	10'	626
**NMO-EXP55-A**	flake	End-scraper	50 x 39 x 14	left	45	st	st	58	scraping	antler	soaked	50 - 70º	10'	600
				right	30	st	st	56	scraping	antler	soaked	50 – 70º	5'	350
NMO-EXP56-A	flake	U. flake	44 x 34 x 7	right	28	st	st	43	scraping	antler	soaked	50 - 70º	20'	1524
NMO-EXP57-A	point	Side-scraper	47 x 40 x 8	dis	4	st	st	87	grooving	antler	soaked	50 - 70º	5'	100
NMO-EXP58-A	flake	U. flake	47 x 22 x 3	right	40	st	st	50	sawing	antler	soaked	90º	15'	1600

NMO-EXP59-A	blade	Notch	60 x 22 x 4	left + distal	7	st	st	55	grooving	antler	soaked	90º	10'	230
NMO-EXP60-C	flake	Side-scraper	42 x 26 x 4	right	40	st	st	36	slicing	meat, fat, tendon	fresh	50 - 70º	55'	3465
NMO-EXP61-M	blade	Naturally backed knive	94 x 47 x 15	left	41	st	st	70º	scraping	wood	fresh	70 - 90º	25'	1810
**NMO-EXP62-P**	point	Non-Levallois point	65 x 17 x 8	left	57	st	inc	54	scraping	hide	fresh	70 - 80º	20'	1000
				right	55	sin	inc	32	cutting	hide	fresh	70 – 90º	20'	1350
NMO-EXP63-P	point	Non-Levallois point	50 x 17 x 4	right	43	sin	st	40	cutting	hide	fresh	70 - 90º	5'	273
**NMO-EXP64-M**	point	Non-Levallois point	50 x 20 x 5	left	38	st	st	38	scraping	wood	fresh	60 - 80º	10'	800
				right	45	st	st	45	whittling	wood	fresh	40 - 60º	10'	250
NMO-EXP65-M	blade	Notch / Side-scraper	33 x 14 x 4	left	31	st	st	63	whittling	wood	fresh	30 - 50º	30'	1570
NMO-EXP66-M	flake	Notch / Side-scraper	35 x 19 x 4	left	11	st	st	84	scraping	wood	fresh	30 - 50º	20'	1515
NMO-EXP67-O	point	Non-Levallois point	63 x 23 x 6	left	43	st	inc	56	scraping	ocher	dry	60 - 80º	10'	1180
NMO-EXP68-HR	blade	U. blade	55 x 27 x 5	left	45	st	st	56	sawing	horn	dry	80 - 90º	20'	2570
NMO-EXP69-M	blade	Naturally backed knive	93 x 38 x 12	left	54	st	st	43	whittling	wood	fresh	40 - 60º	45'	1755

Supplementary table S1. Handheld experiments included in the reference collection. U. flake = unretouched flake; U. blade = unretouched blade; L x W x T = length x Width x Thickness; Uedge = used edge; LUedge = Length Used edge (mm); HEpro = Horizontal Edge profile (sin = sinuous, st = straight, cx = convex, cc = concave, dent = denticulate, st (cc) = straight with a tendency to concave, st (cx) = straight with a tendency to convex, st (sin) = straight with a tendency to sinuous, cc (st) = concave with a tendency to straight, cx (st) = convex with a tendency to straight, Cx (sin) = convex with a tendency to sinuous); SEpro = Sagital Edge profile (st = straight, inc = incurved, st (inc) = straight with a tendency to incurved, Inc (sin) = incurved with a tendency to sinuous, Inc (st) = incurved with a tendency to straight); Eangle = Edge angle; Wmat = worked material; SWmat = state worked material; Wangle = working angle; Wtime = working time. *Artifacts with two used edges are marked in bold.

Tool ID	Blank	Tool Type	L x W x T	Ktech	Hammer	REdge	Rtech	Hretouch
NMO-EXP70-L	flake	denticulate	50 x 38 x 12	direct free hand percusion	limestone	left / right	direct free hand percussion with the tool gripped in the left hand and resting on a piece of leather.	limestone
NMO-EXP71-Q	flake	denticulate	51 x 33 x 10	direct free hand percusion	quatzite	left / right	direct free hand percussion with the tool gripped in the left hand and resting on a piece of leather	quatzite
NMO-EXP72-AH	point	double convex side scraper	52 x 40 x 13	direct free hand percusion	Deer antler	left / right	direct free hand percussion with the tool gripped in the left hand and resting on a piece of leather	deer antler
NMO-EXP73-S	flake	end-scraper	55 x 28 x 12	direct free hand percusion	sandstone	left / distal	direct free hand percussion with the tool gripped in the left hand and resting on a piece of leather	sandstone
NMO-EXP74-S	flake	notch	34 x 16 x 8	direct free hand percusion	sandstone	right	direct percussion on anvil (sandstone)	sandstone
NMO-EXP75-S	flake	end-scraper	23 x 37 x 8	direct free hand percusion	sandstone	distal	direct percussion on anvil (limestone)	sandstone
NMO-EXP76-S	flake	double convex side-scraper	27 x 30 x 7	direct free hand percusion	sandstone	left / right	direct percussion on anvil (limestone)	sandstone
NMO-EXP77-S	flake	retouched flake	25 x 17 x 6	direct free hand percusion	sandstone	distal / proximal / right	direct percussion on anvil (limestone)	sandstone
NMO-EXP78-S	point	notch	40 x 16 x 8	direct free hand percusion	sandstone	left	direct percussion on anvil (limestone)	sandstone

NMO-EXP79-S	flake	notch	20 x 17 x 5	direct free hand percusion	sandstone	left / right	direct percussion on anvil (limestone)	sandstone
NMO-EXP80-S	blade	double side-scraper	33 x 16 x 4	direct free hand percusion	sandstone	left / right / distal	direct free hand percussion with the tool gripped in the left hand and resting on a piece of leather	sandstone
NMO-EXP81-S	flake	denticulate / side-scraper	21 x 20 x 5	direct free hand percusion	sandstone	left / right	direct percussion on anvil (limestone) for the left edge / direct free hand percussion for the right edge	sandstone

Supplementary table S2. Experimental stone tools used as a control reference for the recognition of production wear. All tools remained unused. L x W x T = Length x Width x Thickness; Ktech = knapping technique; REdge = retouched edge; Rtech = retouch technique; Hretouch = hammer used for retouching.

REFERENCE	Blank	Tool Type	L x W x T	UEdge	LUEdge	Eangle	HEpro	SEpro	Activity	Wmat	SWmat	WAngle	WTime	Strokes	Htype	Hmethod	Hmat	Tplacement	Tdir	OApart	Fixation
NMO-EXP06-C	flake	R. Flake	24 x 50 x 7	distal	35	12	sin	inc	cutting	meat, tendon, bone	fresh	70 - 80º	44'	2200	male split	direct	wood (*Quercus ilex*)	terminal	axial	Par	vegetal bindings
NMO-EXP08-P	flake	End-Scraper	41 x 36 x 9	right	40	37	dent	st	scraping	hide	fresh	70 - 80º	20'	1500	male split	direct	wood (*Quercus ilex*)	terminal	axial	Per	vegetal bindings
NMO-EXP20-C	blade	Notch	36 x 18 x 4	left	33	36	st	st	cutting	meat	fresh	60 - 80º	45'	1800	male notched	direct	dry wood (Salix s.p.)	lateral	axial	Par	vegetal bindings
**NMO-EXP21-P**	point	Non-Levallois Retouched point	85 x 21 x 5	left	45	37	sin	inc	scraping	hide	dry	60 - 80º	60'	4200	male notched	direct	dry wood (*Fraxinus s.p.)*	terminal	axial	Par	vegetal bindings
				right	45	25	inc	st	cutting	hide	dry	60 - 80º	30’	1470							
**NMO-EXP38-H**	blade	U. Blade	58 x 17 x 3	left	47	47	st	st	scraping	bone	fresh	50 - 70º	10'	428	male notched	direct	wood (*Fraxinus*)	terminal	axial	Par	vegetal bindings
				right	51	22	sin	st	scraping	bone	fresh	50 - 70 º	5’	313							

Suplementary table S3. Hafting experiments included in the reference collection. L x W x T = Length x Width x Thickness; Uedge = used edge; LUedge = Length Used edge (only active part in mm); Eangle = Edge angle; HEpro = Horizontal Edge profile (Sin = sinuous, St = straight, cc = concave, Dent = denticulate, inc = incurved; SEpro = Sagital Edge profile (st = straight, inc = incurved), Wmat = worked material; SWmat = State Worked material; Wangle = working angle; Wtime = working time; Htype = haft type, Hmethod = Hafting method; Hmat = haft material; Tplacement = tool placement on the haft; Tdir = tool direction (how the tool was inserted in relation to the axis of the haft, axial = stone tool oriented parallel in relation to the axis of the haft); OApart = orientation of the active part in relation to the axis of the haft (par = parallel, per = perpendicular). *stone tools with two used edges are marked in bold.

Tool ID	Blank	Tool Type	L x W x T	Uedge	LUedge	HEpro	SEpro	Eangle	Umot	Rs	Activity	Rs	Mhardness	Rs	Wmat	Rs	H	Rs	Mode Rs
NMO11 2922	Blade	Naturally backed Knife	71 x 26 x 8	Left	65	sin	inc	37	L	4	Cutting	4	Soft	4	Soft animal tissue (Butchery)	4	X	2	4
**NMO11 2929**	Blade	Retouched blade	94 x 30 x 9	Left	86	st	st	44	L	4	Cutting	4	Soft	4	Soft animal tissue (Butchery)	4	X	3	4
				Right	88	st (cx)	st (inc)	35	L	4	Cutting	4	Soft	4	Soft animal tissue (Butchery)	4			4
NMO11 2936	Blade	Naturally backed knife	88 x 24 x 14	Right	80	st	st	47	L	4	Cutting	4	Soft-medium	4	Soft animal tissue (Butchery)	4			4
									T	4	Scraping	4	Hard	4	Bone	2			4
NMO12 3535	Blade	Naturally backed knife	98 x 34 x 14	Left	85	sin	inc	31	L	3	Cutting	3	Soft	3	Indt	0			3
NMO14 4345	Blade	Naturally backed knife	86 x 33 x 11	Right	80	sin	st	38	L	4	Cutting	4	Soft	4	Soft animal tissue (Butchery)	4			4
NMO14 4415	Blade	Naturally backed knife	84 x 32 x 12	Right	80	st (sin)	inc	38	T	4	Whittling	4	Medium-Hard	4	Wood/woody plant	4	X	4	4
**NMO07 138**	Point	Levallois Point	72 x 44 x 12	Left	65	sin	inc	44	Indt	0	Indt	0	Indt	0	Indt	0	X	1	0
				Right	69	sin	inc	35	Indt	0	Indt	0	Indt	0	Indt	0			0
**NMO12 3522**	Flake	Retouched flake	82 x 46 x 12	Left	77	sin	st	36	R	2	Boring	2	Hard	2	Indt	0	X	2	2
				Right	69	sin	st	35											
NMO09 2200	Point	Levallois point	81 x 31 x 11	Left	70	st	inc	46	T	4	Scraping	4	Soft-medium	4	Dry hide	3	X	4	4
**NMO10 2661**	Point	Levallois point	48 x 42 x 13	Left	40	cx	st	55	L	4	Cutting	4	Soft	4	Soft animal tissue (Butchery)	4			4
				Right	35	sin	inc	43	L	4	Cutting	4	Soft	4	Soft animal tissue (Butchery)	4			4
**NMO12 3527**	Point	Retouched Levallois point	89 x 46 x 10	Left	50	cx	inc	32	L	3	Projectile	3	Hard	3	Animal carcass (Impact)	3	X	3	3
				Right	75	st	inc	50											

NMO10 2560	Point	Levallois Point	101 x 37 x 13	Left	93	st	Inc	50	L	3	Cutting	3	soft	3	Soft animal tissue (Butchery)	2	X	1	3
**NMO11 2886**	Point	Levallois point	72 x 34 x 7	Left	67	st	Inc	31	L	4	Cutting	4	Soft	4	Soft animal tissue (Butchery)	3	X	2	4
				Right	65	st	st (inc)	37	L	4	Cutting	4	soft	4	Soft animal tissue (Butchery)	3			4
**NMO13 3942**	Point	Retouched blade	94 x 30 x 9	Left	87	sin	inc	47	Indt	0	Indet	0	Indet	0	Indt	0	X	2	0
				Right	85	st (sin)	inc	58	Indt	0	Indet	0	Indet	0	Indt	0			0
**NMO13 3955**	Point	Notch	61 x 32 x 8	Left	53	st	st	40	L	4	Cutting	4	Soft	4	Soft animal tissue (Butchery)	4			4
				Right	54	cx (dent)	st	40	L	4	Cutting	4	Soft	4	Soft animal tissue (Butchery)	4			4
**NMO13 3962**	Point	Notch	68 x 29 x 8	Left	60	st (sin)	inc	35	L	4	Cutting	4	Soft	4	Soft animal tissue (Butchery)	4	X	3	4
				Right	59	sin	inc	47	L	4	Cutting	4	soft	4	Soft animal tissue (Butchery)	4			4
**NMO14 4303**	Point	Notch	70 x 43 x 12	Left	63	dent	st	52	L	2	Cutting	2	Soft-medium	2	Soft animal tissue (Butchery)	1	X	2	2
				Right	63	dent	inc	62	L	2	Cutting	2	Soft-medium	2	Soft animal tissue (Butchery)	1			2
NMO14 1953	Flake	Denticulate	43 x 43 x 9	Left	34	dent	st	35	L	4	Cutting	4	Soft	4	Hide	4			4
NMO12 3644	Flake	Flake	61 x 21 x 5	Right	44	st	st	34	T	4	Whittling	4	Medium-Hard	4	Wood	3			4
NMO13 3868	Flake	Flake	60 x 34 x 6	Left	51	sin	st	18	L	3	Cutting	4	Soft	3	Soft animal tissue (Butchery)	2			3
**NMO13 4047**	Flake	Flake	58 x 33 x 8	Left	31	st	st	41	L	4	Cutting	4	Soft	4	Soft animal tissue (Butchery)	4			4
				Right	50	sin	inc	40	L	4	Cutting	4	Soft	4	Soft animal tissue (Butchery)	4			4
NMO13 4123	Flake	Flake	25 x 22 x 5	Right	32	sin	sin	52	L	4	Cutting	4	Soft	4	Dry Hide	4			4
NMO14 4441	Flake	Flake	42 x 22 x 9	Left	40	st	st	45	L	4	Cutting	4	Soft	4	Hide	4			4
NMO14 2183	Flake	Notch	38 X 30 x 9	Right	22	st	st	47	T	4	Scraping	4	Hard	4	Bone	4			4
NMO12 3647	Flake	Naturally backed knife	38 x 22 x 8	Left	30	sin	inc	26	L	4	Cutting	4	Hard	4	Hard animal tissue (Butchery)	4			4

NMO09 2242	Flake	Flake	54 x 30 x 9	Right	47	st	st	37	L	4	Cutting	4	Soft	4	Soft animal tissue (Butchery)	3			4
**NMO10 2664**	CTE	Retouched flake	49 x 44 x 16	Left	10	st	st	73	T	4	Scraping	4	Hard	4	Bone	3			4
				Right	35	cx	inc	30	T	4	Scraping	4	Hard	4	Bone	4			4
NMO14 1929	Flake	Flake	46 x 35 x 8	Right	33	sin	st	39	T	4	Scraping	4	hard	4	Bone	4			4
NMO14 4416	Flake	Naturally backed knife	27 x 18 x 6	Left	20	sin	inc	43	L	4	Cutting	4	Soft	4	Hide	4			4
**NMO11 2892**	Flake	Flake	51 x 25 x 7	Left	46	sin	st	22	L	4	Cutting	4	Hard	4	Hard animal tissue (Butchery)	4			4
				Right	47	sin	st	38	T	4	Scraping	4	Hard	4	Bone	4			4
**NMO14 4307**	Point	Blade	81 x 38 x 12	Left	70	cx	inc	35	L	4	Cutting	4	Soft	4	Hide	4	X	3	4
				Right	73	sin	inc	41	L	4	Cutting	4	Soft	4	Hide	4			4
**NMO13 3885**	Flake	Retouched Flake	64 x 44 x 10	Left	48	cx	inc	52	L	4	Cutting	4	Soft	4	Soft animal tissue (Butchery)	4			4
				Right	21	st	st	25	L	4	Cutting	4	Soft	4	Soft animal tissue (Butchery)	4			4
NMO08 2017	Blade	Typical End scraper	87 x 40 x 18	Distal	20	cx	st	67	T	4	Scraping	4	Medium	4	Wood	3	X	3	4
**NMO10 2628**	Flake	Atypical Levallois flake	68 x 36 x 10	Left	48	cx	st	42	L	4	Cutting	4	Soft	4	Hide	4			4
				Right	56	cc	st	47	L	4	Cutting	4	Soft	4	Hide	4			4
**NMO14 4361**	Blade	Naturally backed knife	81 x 38 x 14	Left	66	cx	inc	41	L	4	Cutting	4	Soft	4	Soft animal tissue (Butchery)	4	X	4	4
				Right	41	sin	st	43	L	4	Cutting	4	Soft	4	Soft animal tissue (Butchery)	4			4

NMO14 4453	Flake	Retouched flake	41 x 33 x 11	Left	33	cc (st)	inc	41	T	4	Whittling	4	Medium-Hard	4	Wood	3			4
NMO11 3102	Flake	Denticulate	54 x 29 x 14	Left	42	dent	sin	48	L	4	Cutting	4	Soft	4	Hide	4			4
**NMO14 1950**	Point	Levallois point	48 x 45 x 10	Left	44	st	inc	53	L	4	Cutting	3	Hard	4	Hard animal tissue (Butchery)	4			4
				Right	37	st	st	58	T	4	Scraping	4	Hard	4	Bone	4			4
**NMO08 2021**	Point	Levallois point	47 x 26 x 8	Left	40	sin	st	40	L	4	Cutting	4	Soft-medium	4	Soft animal tissue (Butchery)	4	X	3	4
				Right	45	sin	inc	35	L	4	Cutting	4	Soft-medium	4	Soft animal tissue (Butchery)	4			4
NMO14 4507	Blade	Naturally backed knife	83 x 37 x 11	Right	70	st	inc	37	T	4	Scraping	4	Hard	4	Bone/Antler	4			4
NMO12 3612	Flake	Flake	40 x 53 x 15	Distal	45	st	st	65	L	4	Cutting	4	Soft-medium	4	Soft animal tissue (Butchery)	4			4
NMO07 1555	Blade	Naturally backed knife	55 x 26 x 12	Left	52	st	st	37	T	4	Scraping	3	Hard	3	Bone	3			3
NMO11 2912	Flake	Naturally backed knife	38 x 23 x 12	Right	34	st	st	45	L	4	Cutting	4	Soft	4	Dry hide	4			4
NMO08 2054	Flake	Typical Levallois Flake	44 x 30 x 10	Right	30	cx	st	33	T	3	Whittling	4	Medium-Hard	4	Wood/woody plant	4			4

NMO08 2032	Point	Retouched Levallois point	47 x 35 x 9	Left	37	st	st	47	L	4	Cutting	4	Soft	4	Soft animal tissue (Butchery)	4			4
NMO08 2027	Blade	Naturally backed knife	74 x 34 x 12	Right	49	st (cc)	st (inc)	57	L	4	Cutting	4	soft	4	Dry Hide	4			4
NMO14 4472	Blade	Naturally backed knife	70 x 28 x 19	Right	50	st	st	53	T	4	Scraping	4	Hard	4	Bone	4			4
**NMO13 4075**	Blade	Naturally backed knife	74 x 34 x 13	Left	48	cx	st	30	T	4	Scraping	4	Soft-medium	4	Dry Hide	4	X	2	4
				Right	54	sin	inc	50	L	4	Cutting	4	Soft	4	Hide	4			4
NMO14 4351	Flake	Notch	66 x 39 x 9	Right	51	st	st	45	L	4	Cutting	4	Soft	4	Soft animal tissue (Butchery)	4			4
NMO12 3793	Blade	Naturally backed knife	86 x 37 x 21	Right	76	sin	inc	58	T	3	Scraping	3	Hard	4	Bone	3			3
**NMO08 2122**	Blade	Blade	70 x 25 x 9	Left	62	st	inc	40	T	4	Whittling	4	Medium-Hard	4	Wood	4			4
				Right	60	st	inc	35	T	4	Whittling	4	Medium-Hard	4	Wood	4			4
**NMO11 2931**	Blade	Retouched Blade	62 x 28 x 12	Left	47	st	st	47	L	4	Cutting	4	Soft	4	Soft animal tissue (Butchery)	4			4
				Right	37	st	st	34	L	4	Cutting	4	Soft	4	Soft animal tissue (Butchery)	4			4
**NMO14 4321**	Point	Blade	59 x 22 x 7	Left	55	st	inc	34	L	4	Cutting	4	Soft	4	Soft animal tissue (Butchery)	4	X	3	4
				Right	26	st	inc	26	L	4	Cutting	4	Soft	4	Soft animal tissue (Butchery)	4			4
NMO09 2204	Point	Point	61 x 28 x 7	Right	57	sin	st	25	R	1	Boring	1	Soft	1	Indt	0			1
NMO14 4489	Flake	Retouched Flake	32 x 18 x 7	Left	28	st	st	40	L	2	Cutting	2	Soft	3	Soft animal tissue (Butchery)	2			2

NMO10 2657	Point	Mousterian point	45 x 25 x 5	Left	33	st	st	35	L	3	Cutting	3	Soft-medium	3	Soft animal tissue (Butchery)	2			3
NMO11 3149	Point	Non-Levallois Point	45 x 22 x 7	Left	40	sin	st	27	L	4	Cutting	4	Soft-medium	4	Plants	4	X	4	4
NMO14 4339	Flake	Flake	72 x 44 x 12	Left	60	st	st	40	L	4	Cutting	4	Soft	4	Soft animal tissue (Butchery)	2			4
**NMO14 4480**	Point	Levallois point	31 x 25 x 6	Left	23	st	st	51	L	3	Projectile	1	Hard	3	animal carcass (hunting)	1	X	1	1
				Right	30	st	st	35											
NMO 08 2069	Flake	Flake	52 x 30 x 7	Left	43	sin	inc	33	L	3	Cutting	3	Soft	3	Indt	0			3
NMO12 3559	Flake	Flake	61 x 34 x 8	Left	59	st (sin)	inc	40	L	4	Cutting	4	Soft	4	Hide	4			4
**NMO13 3873**	Flake	Flake	49 x 41 x 11	Left	32	st	inc	40	L	4	Cutting	4	Soft	4	Soft animal tissue (Butchery)	4			4
				Right	33	sin	inc	35	L	4	Cutting	4	Soft	4	Soft animal tissue (Butchery)	4			4
**NMO12 3819**	Blade	Retouched blade	77 x 27 x 10	Left	73	sin	inc	48	L	2	Cutting	2	Soft	2	Soft animal tissue (Butchery)	2	X	1	2
				Right	67	cc	st	37	L	2	Cutting	2	Soft	2	Soft animal tissue (Butchery)	2			2
**NMO14 4311**	Flake	Flake	38 x 29 x 9	Left	33	sin	inc	35	L	4	Cutting	4	Soft-medium	4	Soft animal tissue (Butchery)	4			4
				Right	34	sin	inc	40	L	4	Cutting	4	Soft-medium	4	Soft animal tissue (Butchery)	4			4
NMO13 3980	Flake	Naturally backed Knife	41 x 24 x 8	Right	29	st (cc)	inc	39	T	4	Scraping	4	Hard	4	Antler	4			4

NMO12 3531	Flake	Denticulate	75 x 54 x 9	Left	38	st	st	34	L	3	Cutting	3	Medium-hard	3	Plants	2			3
**NMO10 2538**	Flake	Flake	37 x 28 x 8	Left	25	st (sin)	st	42	L	4	Cutting	4	Soft	4	Soft animal tissue (Butchery)	4			4
				Right	31	st	inc	40	L	4	Cutting	4	Soft	4	Soft animal tissue (Butchery)	4			4
NMO08 2052	Flake	Flake	48 x 27 x 6	Left	36	st	st	47	L	4	Cutting	4	Soft	4	Soft animal tissue (Butchery)	4	X	3	4
NMO08 2093	Flake	Flake	40 x 34 x 6	Right	20	st	st	28	L	2	Cutting	2	Soft	2	Soft animal tissue (Butchery)	2			2
**NMO10 2627**	Flake	Denticulate	79 x 49 x 8	Left	62	st	st	35	L	4	Cutting	4	Soft-medium	4	Soft animal tissue (Butchery)	4			4
				Right	69	st	st	35	L	4	Cutting	4	Soft-medium	4	Soft animal tissue (Butchery)	4			4
NMO11 2908	Flake	Naturally backed knife	68 x 37 x 9	Left	64	sin	inc	46	L	4	Cutting	4	Soft	4	Soft animal tissue (Butchery)	3			4
NMO13 3860	Flake	Naturally backed knife	66 x 46 x 9	Right	53	sin	st	30	L	3	Cutting	3	Soft	2	Soft animal tissue (Butchery)	2			2

Supplementary table S4. Functional results in the NMO archaeological sample. *Only artifacts with traces of use are included (n = 72). L x W x T = length x Width x Thickness; Uedge = used edge; LUedge = Length Used edge (mm); HEpro = Horizontal Edge profile (sin = sinuous, st = straight, cx = convex, cc = concave, dent = denticulate, st (cc) = straight with a tendency to concave, st (cx) = straight with a tendency to convex, st (sin) = straight with a tendency to sinuous, cc (st) = concave with a tendency to straight, cx (dent) = convex with a tendency to denticulate); SEpro = Sagital Edge profile (st = straight, inc = incurved, st (inc) = straight with a tendency to incurved); Eangle = Edge angle; Umot = Use motion (L = longitudinal, T = transversal, R = rotational, Indt = indeterminate); Rs = Reliability scale for the interpretation of the use motion, activity, material hardness (Mhardness), Worked material (Wmat) and (H) hafting, (0 = uncertain, 1 = poor reliability, 2 = moderate reliability, 3 = high reliability, 4 = reliable interpretation); Mode Rs = Mode reliability scale. *Artifacts with two used edges are marked in bold.

**Activity**		Cutting			Scraping			Whittling			Cutting + Scraping		Projectile	Boring		Indet		
**Blank**	Blade	Flake	Point	Blade	Flake	Point	CTE	Blade	Flake	Blade	Flake	Point	Point	Point	Flake	Point	Totals (%)	Hafted
**W. Material**																		
Butchery	*7 (5)	17 (1)	9 (5)														**33 (46%)**	**11**
Fresh Hide		6	1 (1)														**7 (10%)**	**1**
Dry hide	1	2				1 (1)											**4 (6%)**	**1**
Bone				4	2		1										**7 (10%)**	
Antler					1												**1 (1%)**	
Butchery + Bone										1	1	1					**3 (4%)**	
Dry hide + Fresh Hide										1 (1)							**1 (1%)**	**1**
Projectile													2 (2)				**2 (3%)**	**2**
Wood				1 (1)				2 (1)	3								**6 (8%)**	**2**
Plants		1	1 (1)														**2 (3%)**	**1**
Indet	1	1												1	1 (1)	2 (2)	**6 (8%)**	**3**
**Totals (%)	**9** (47%)	**27** (77%)	**11** (64%)	**5** (26%)	**3** (8,5%)	**1** (6%)	**1** (100%)	**2** (11%)	**3** (8,5%)	**2** (11%)	**1** (3%)	**1** (6%)	**2** (12%)	1 (5%)	1 (3%)	**2** (12%)	**72 (100%)**	
Hafted	5	1	7	1		1		1		1			2		1	2		**22 (31%)**
Grand total (%)		**47** (65%)			**10** (14%)			**5** (7%)			**4** (6%)		**2** (3%)	**2** (3 %)		**2** (3%)	**72 (100%)**	

Supplementary table S5. Functional results in the NMO archaeological sample considering the type of blank, worked material, activity and hafted tools. W. Material; Worked Material. CTE; Core Trimming element. * Numbers in parentheses represent the number of hafted pieces within each worked material and type of blank. ** The percentage is calculated according to the total number of used tools per each type of blank.

When working at different scales because of the combination of microscopes, using the nominal magnifications for each of them for comparison can be misleading. For this reason, it is more relevant to pay attention to the Horizontal Field of View (HFOV) reached in each case. To simplify the figure captions, we provide the original magnifications used with the principal microscope's software (Aco-z), and only give necessary details referring to the HFOV when other software was used (e.g., Deltapix) or when a different microscope is added, which can cause confusion. General HFOV equivalences are shown in the following table:

Microscope model	Mag	*HFOV Deltapix/Aco-z	Mag SEM	HFOV SEM
Zeiss Axio Scope A.1	50 x	2265/2942 µ	135 x	2210 µ
	100 x	1130/1467 µ	260 x	1150 µ
	200 x	564.32/726 µ	510 x	585 µ
	500 x	225.93/295 µ	1250 x	239 µ
Hirox KH-8700	35 x	8665 µ	35 x	8514 µ
	50 x	6065 µ	50 x	5960 µ
	100 x	3032 µ	100 x	2980 µ
	140 x	2166 µ	140 x	2125 µ
	150 x	2021.8 µ	150 x	1986 µ
	200 x	1516.4 µ	200 x	1490 µ
	250 x	1213 µ	250 x	1192 µ
	400 x	758. 2 µ	400 x	745 µ
	600 x	505.5 µ	600 x	497 µ
	700 x	433.3 µ	700 x	425 µ
	800 x	379.1 µ	800 x	373 µ
	1000 x	303.3 µ	1000 x	298 µ
	2000 x	151.6 µ	2000 x	149 µ
	3000 x	101.1 µ	3000 x	99 µ
	4000 x	75.8 µ	4000 x	74.5 µ
	5000 x	60.7 µ	5000 x	59,7 µ

Supplementary table S6. Equivalence among the magnifications (Mag) and the horizontal field of view (HFOV) achieved in the three microscope models used in this study (OM, 3D DM, and SEM). *HFOV = value according to DeltaPix and Aco-z software in Zeiss model and monitor size in 3D DM and SEM models. Hirox KH-8700 (21.5''), SEM FEI Quanta 600 (19'').

Supplementary Figures

Archaeological tools

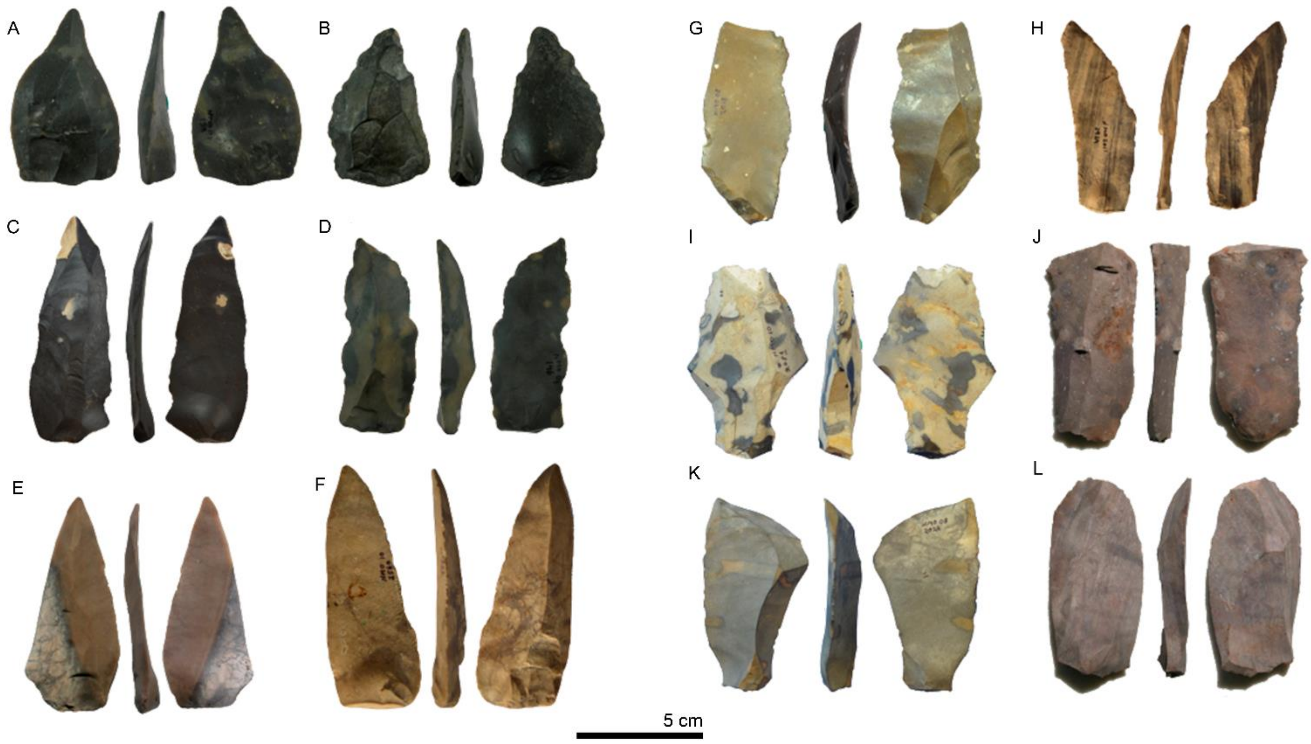

Supplementary figure S1. Pointed (A – F) and cutting elements (G – L) from NMO layer 4.

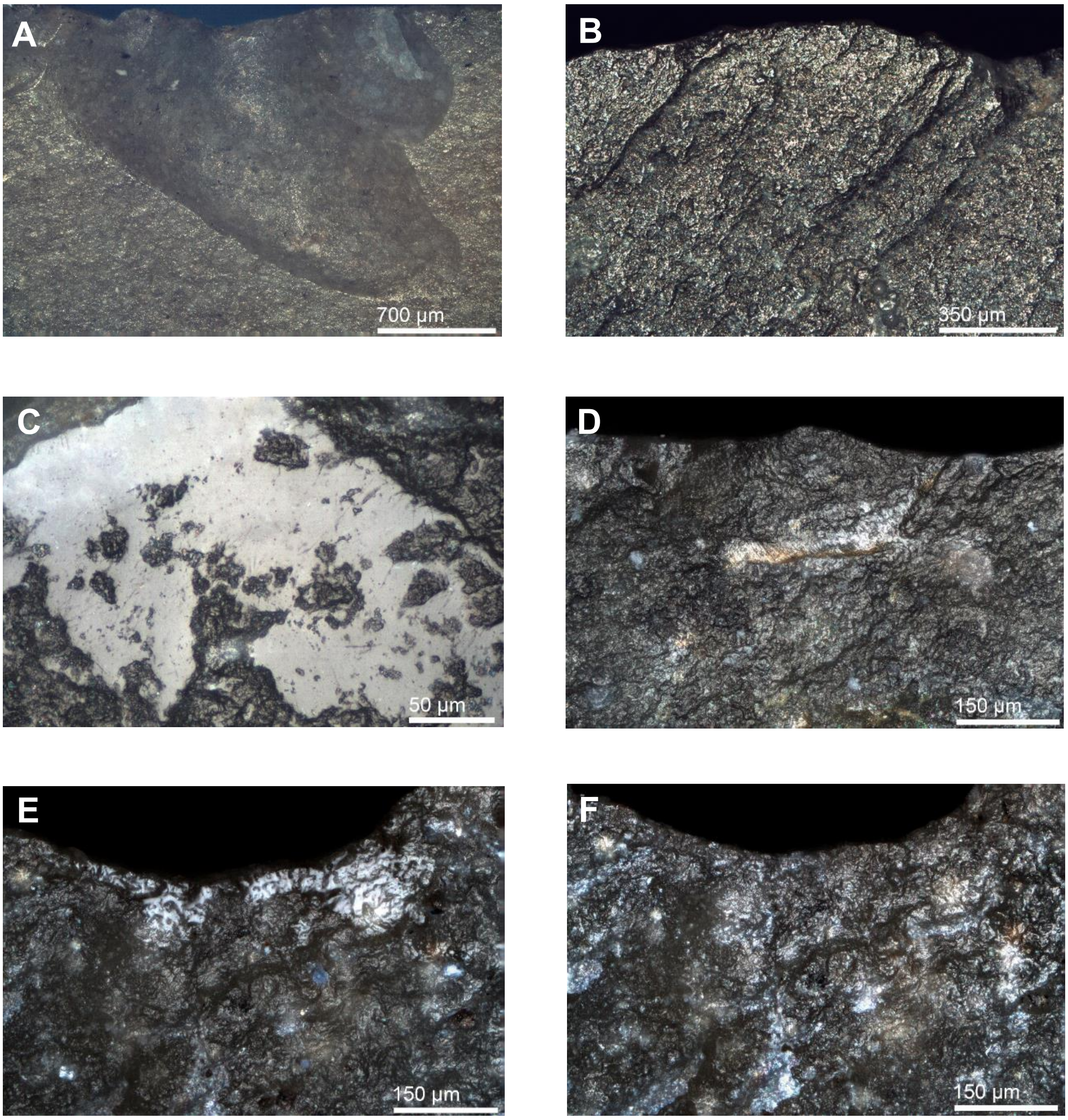

Supplementary figure S2. Different wear features caused by non-use related processes in the NMO lithic assemblage. A, intrusive and oblique step-hinge terminated scars on the left dorsal edge of tool NMO08 2400 recovered from surface, 50x; B, glossy and abraded surface on the right dorsal edge of tool NMO 3524, 100x; C, extensive friction bright spot on the right dorsal edge of tool NMO14 4557, 500x; D, possible storage friction wear on the right dorsal edge of tool NMO09 2201, 200x; E, liquid spot formed on the right dorsal edge of tool NMO08 2066 as a consequence of inadequate drying after 5 minutes of ultrasonic cleaning with pure acetone, 200x; F, disappearance of the same spot after re-cleaning with the same product, 200x.

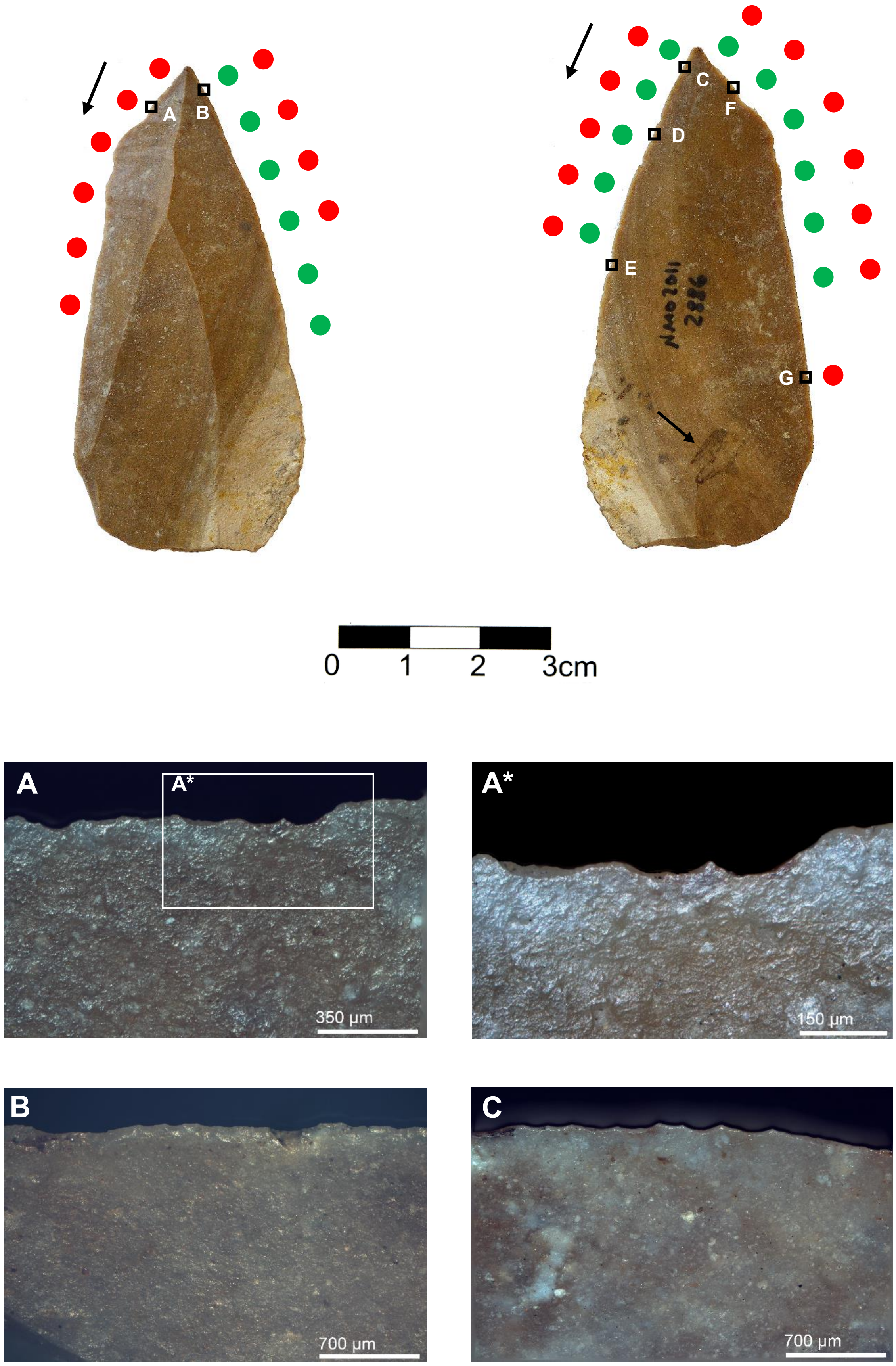
A
B
C
D
E
F
G
0
1
2
3cm
A
A*
350 µm
A*
150 µm
B
700 µm
C
700 µm

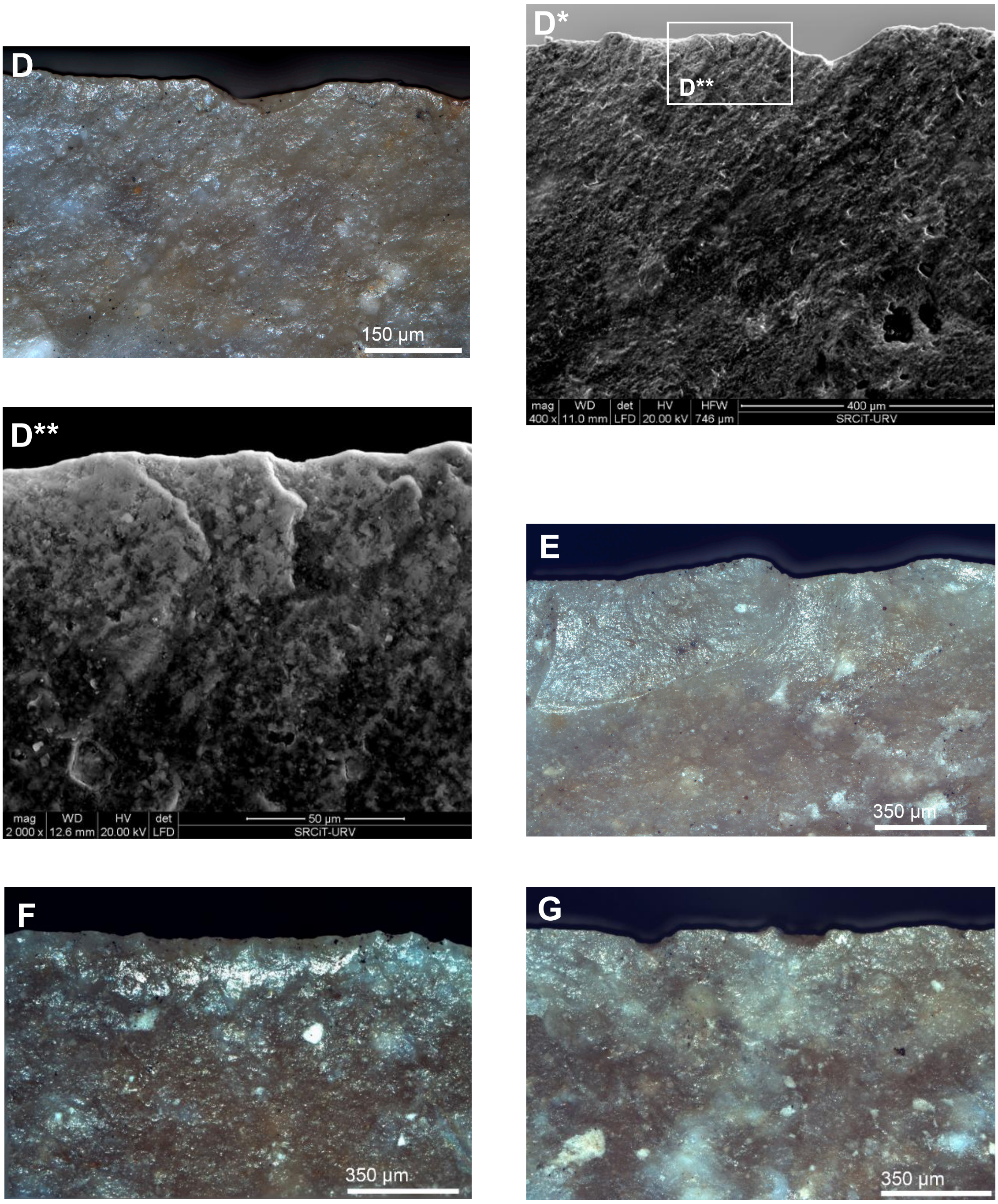

Supplementary figure S3. Use-wear on both lateral edges of Levallois point NMO11 2886. A, *sliced scars and weak polish on the distal dorsal left edge, 100x; A*, close-up of the previous image showing the weak polish and light edge rounding associated with sliced scars, 200x; B, feather terminated scalar scars on the distal dorsal right edge, 50x; C, sliced scars on the distal ventral right edge, 50x; D, polish band and micro-scarring on the ventral distal right edge, 200x; D*, previous area under SEM in which the polished area next to the edge can be identified, 400x; D**, close-up of the previous SEM image in which the polish texture and edge rounding are now visible, which contrast with the unpolished, black, areas further inside, 2000x; E, shallow intrusive scalar scars with feather/step termination on the ventral mesial right edge, 100x; F, feather scalar scars and polish on the ventral distal left edge, 100x; G, weak polish and micro scars on the ventral proximal left edge interpreted as possible hafting wear, 100x. *Red dots depict the areas of the working edges where polish is located; in contrast, green dots depict the location of edge scarring. Black arrow in ventral surface indicate a smeared black-brownish residue. The other black arrows indicate the direction of work. This is valid for all supplementary figures.

Interpretation

Levallois point with excellent surface preservation. Scalar and sliced scars and half-moon fractures are dominant on the distal and mesial parts on both lateral edges (A, B, C, D). Light edge rounding and weak polish are visible on elevated areas and scar ridges, but the polish is not invasive. The wear traces are more developed on the right lateral edge than on the left edge. Feather terminated scalar scars, and sliced scars are present on the ventral distal right edge associated with weak, rough polish (D, E). The texture of this polish is visible at SEM at high magnifications (D**). A snap fracture is located on the distal tip, and intense edge scarring is located near the tip on both lateral edges on the dorsal and ventral faces. A different scar pattern is present on the ventral mesial right edge, which consists of shallow scalar scars with step and feather terminations (F). Gloss and weak polish and rounding were located on the proximal ventral left edge (H). All the wear features and patterns indicate that the tool was used in a butchery activity with both edges, possibly while hafted. The presence of a brownish-black residue smear on the ventral proximal surface near the bulb (arrow) suggest that some glue may have been used to haft the tool. However, considering that the tool was recovered from an area with a high density of black mud, this residue smear cannot be used as inequivocal evidence of hafting until further analysis is performed (SEM-EDX, Raman, Micro-FTIR, etc.).

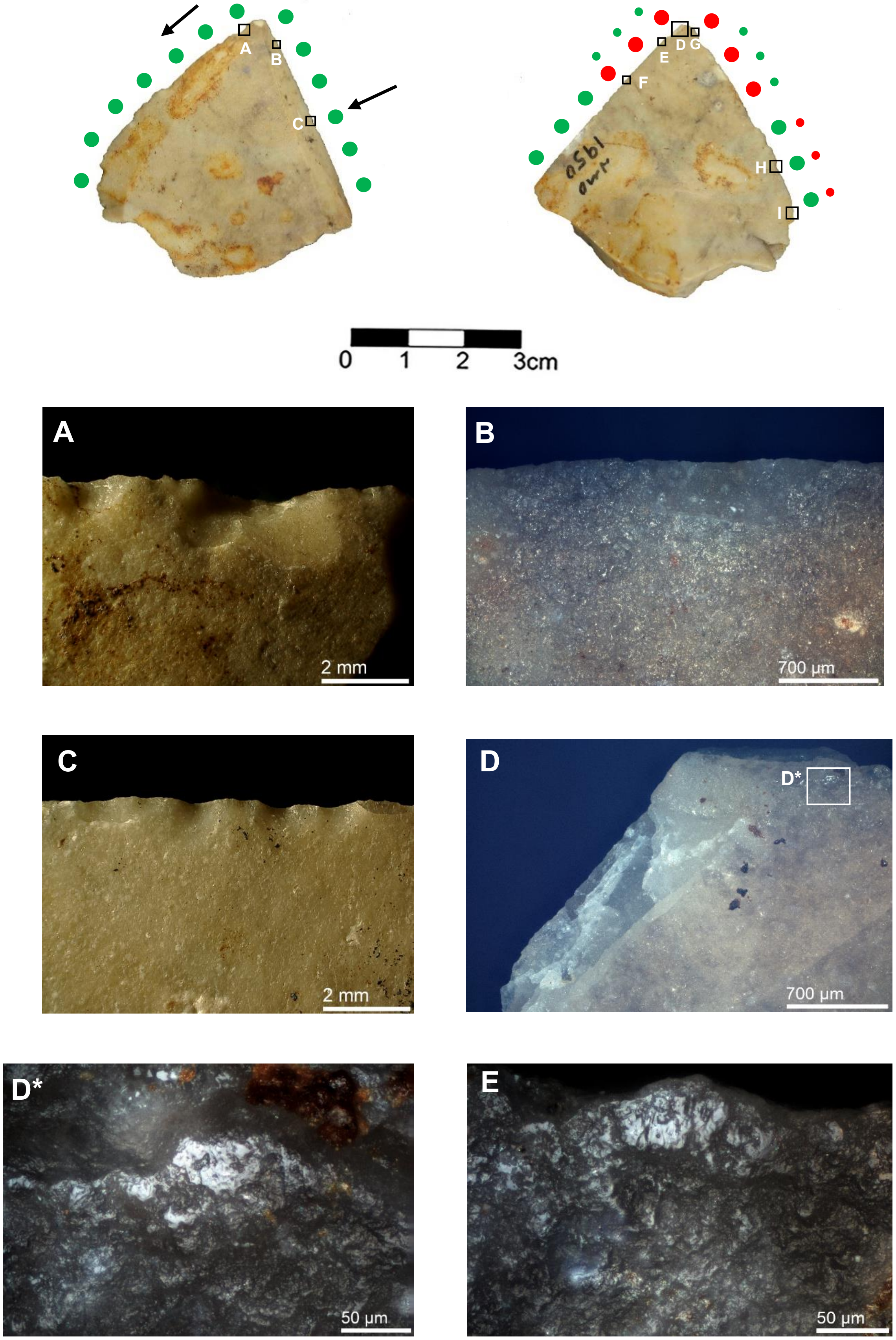
A
B
C
D
G
E
F
H
I
0
1
2
3cm
A
2 mm
B
700 µm
C
2 mm
D
D*
700 µm
D*
50 µm
E
50 µm

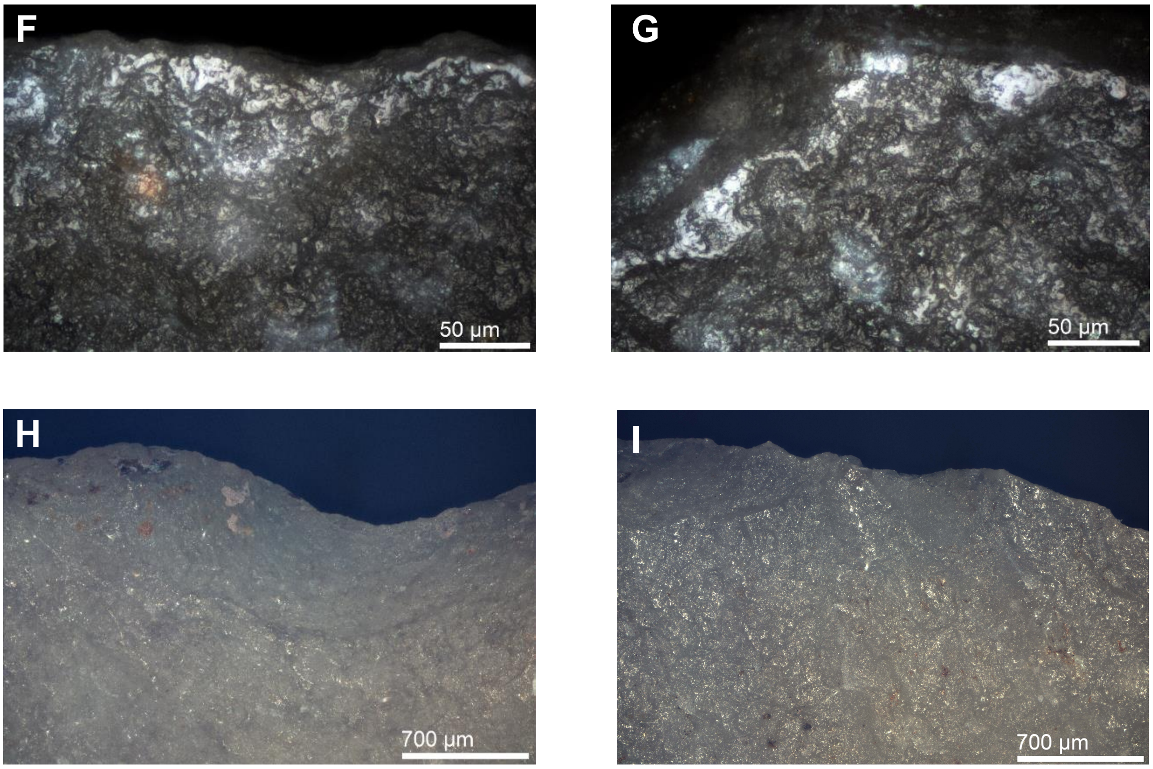

Supplementary figure S4. Use-wear on both lateral edges of Levallois point NMO14 1950. A, scalar scars with shallow step terminations and crushed initiations on the dorsal distal left edge, 35x, HFOV 8.6 mm; B, step trapezoidal scars on the dorsal distal right edge, 50x; C, feather terminated scalar scars showing cone initiations on the dorsal mesial right edge, 35x, HFOV 8.6 mm; D, trapezoidal scar with crushing and fissured termination on the ventral distal right edge associated with fractures and polish on the distal tip, 50x; D*, close-up of the polish identified on the previous image showing rough-bright texture, 500x; E, smooth-wavy polish with transversal striations and light edge rounding on the ventral distal right edge, 500x; F, smooth-wavy and rough polish associated with light edge rounding on the ventral mesial right edge, 500x; G, bright polish with flat-wavy topography on the ventral distal left edge close to the tip, 500x; H, intrusive large scalar scar with feather termination on the ventral proximal left edge, 50x; I, scalar scars with feather termination and weak invasive polish on the scar ridges on the ventral proximal left edge, 50x.

Interpretation

On the dorsal side of the right lateral edge, trapezoidal and scalar step/feather scars were identified along the whole edge. Step-terminated scars with wide initiations are dominant, with a considerable presence of trapezoidal (B) over cone-initiated and feather terminated scalar scars (C). Well-developed polish was not identified, but weak polish in the high parts of topography between scars. On the ventral face of the same edge well-developed polish with smooth-rough texture and wavy/flat topography is visible. On the distal part, bright smooth polish with wavy topography is associated with light edge rounding (E). The same type of polish is also visible towards the mesial part of the edge (F). From the mesial to the proximal portion of the edge, feather and step-terminating scars are visible. These characteristics suggest that this edge was used in a transversal negative motion for scraping hard animal material, likely bone. The presence of crushing in the initiations of step scars and near the tip suggest an intense and repetitive contact with bone. One example is a trapezoidal scar next to the tip showing crushing and a fissured termination, but its initiation does not start from the main tip but on the right edge (D). Moreover, the presence of rough-bright polish with flat topography on one of the fracture ridges on the tip (D*) suggest frequent contact with bone during scraping.

Slightly different wear patterns are visible on the left lateral edge. Step-terminated scalar scars (A) are the most frequent type of scar on the dorsal face of the edge. No polish was found on the dorsal face, though on the ventral side, smooth-rough polish with flat-wavy topography is visible on the distal and mesial extremities (G). Only few feather terminated scars were identified on the distal and mesial extremities, together with light edge-rounding. From mesial towards the proximal portion of the edge, large feather and step scalar scars are dominant together with an invasive weak polish located on the high topographic parts between these scars (H, I). All these wear traces indicate that the left lateral edge was likely used in a longitudinal use-motion in a butchery activity in which there was frequent contact with bone. The size of the scalar scars, the polish invasiveness on the scar ridges, and the lack of step-terminated scars were crucial for this interpretation of the left lateral edge.

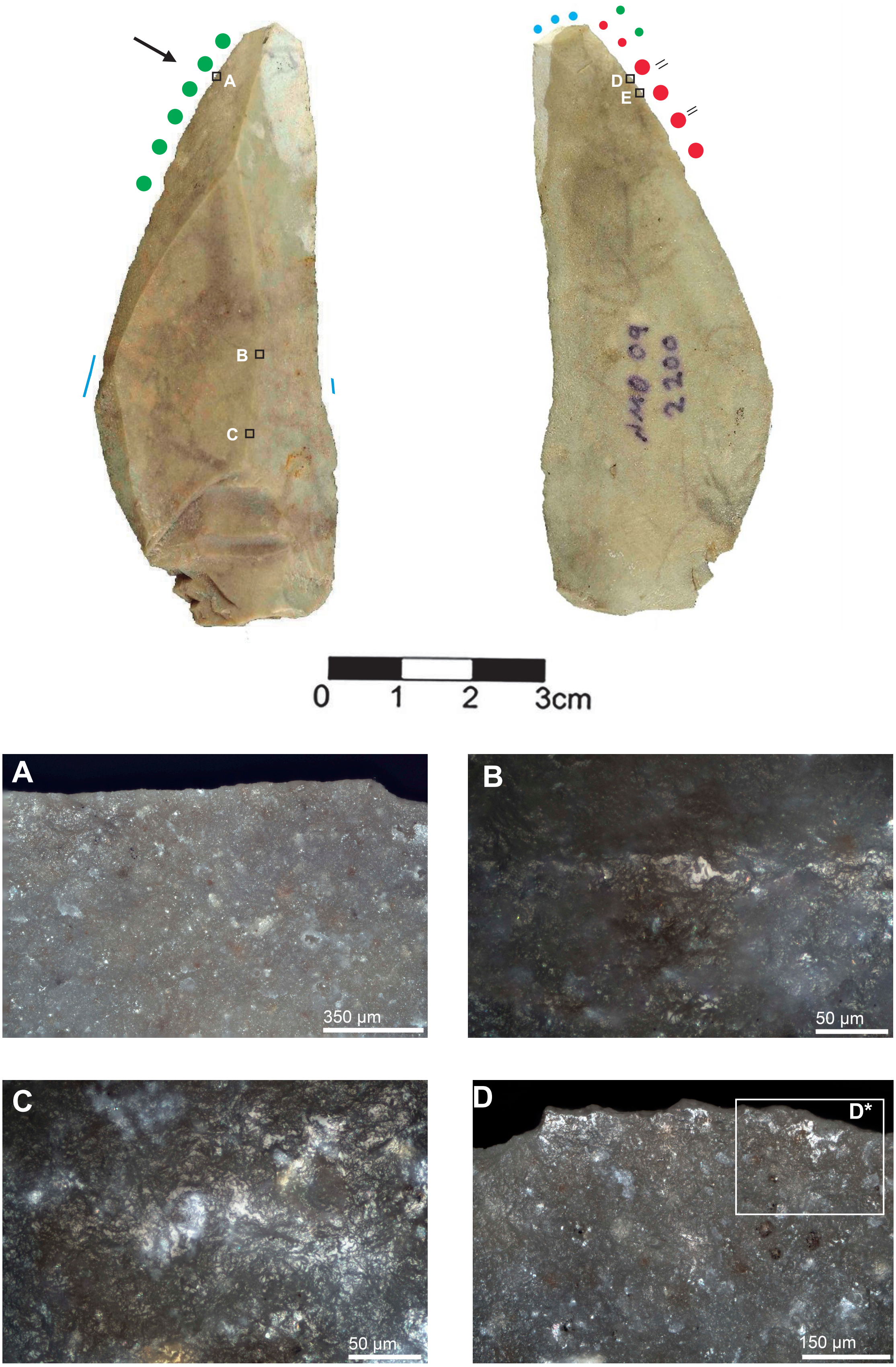
A
B
C
D
E
0 1 2 3cm
A
350 µm
B
50 µm
C
50 µm
D
D*
150 µm

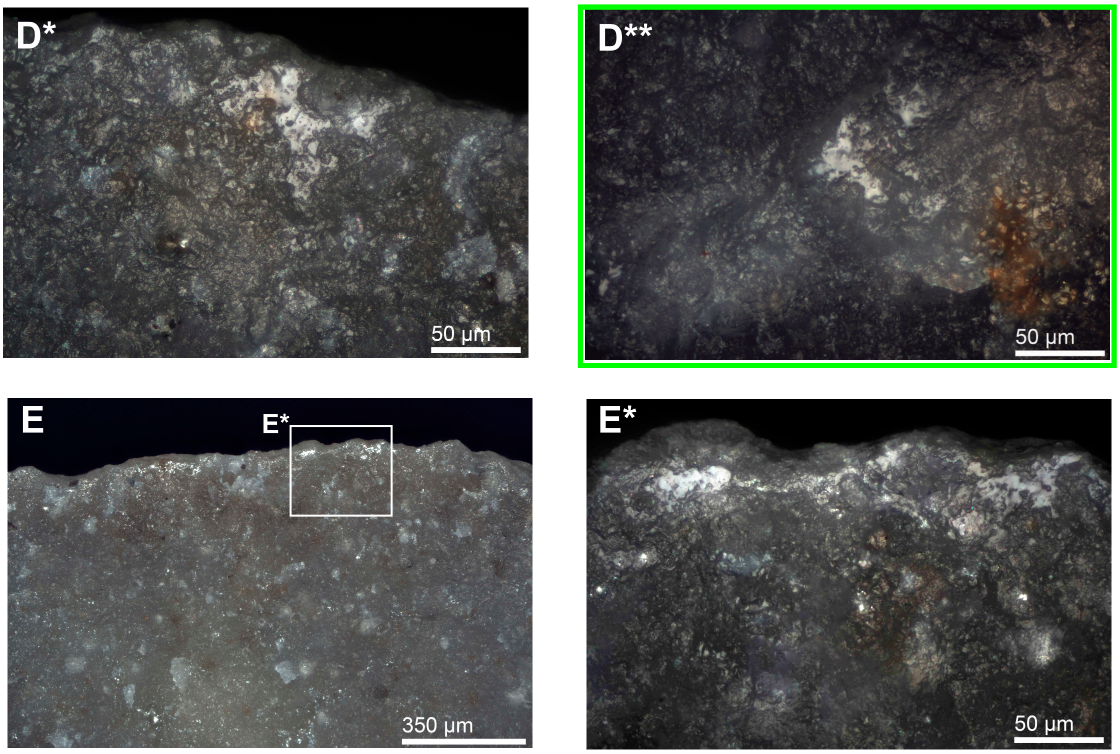

Supplementary figure S5. Levallois point NMO09 2200 showing use-wear on the left edge, as well as hafting wear on the proximal-mesial extremities. A, very shallow scalar scars on the distal dorsal left edge, 100x; B, Hafting polish on the dorsal mesial right ridge, 500x; C, hafting polish on the dorsal proximal right ridge, 500x; D, smooth polish, edge rounding, and micro-scars on the ventral distal left edge, 200x; D* close-up of a polished area showed in D, in which the smooth texture and wavy topography of polish is appreciated, 500x; D**, experimental dry hide polish observed on the tool NMO-EXP17-P, used for cutting dry hide, 2720 strokes, 40 min, 500x; E, well-developed edge rounding and spots of polish on the ventral distal left edge, 100x; E*, Close-up of the polish showed in E in which the well-developed edge rounding and smooth texture of polish are appreciated, 500x. *blue dots on the ventral tip indicate a step-terminating bending fracture. Blue solid line indicate area with hafting traces.

Interpretation

The dorsal left edge presents small, shallow scalar scars with feather termination on the distal extremity (A), and no evidence of well-developed polish was found on this face but only weak polish and smoothing. Hafting polish and bright spots have been recorded on the mesial and proximal dorsal ridges (B, C). On the contrary, well-developed polish was identified on the distal ventral left edge and restricted to the outer edge (E). The polish is smooth, a bit dull, and is showing wavy topography and a few pits (D*), with some spots showing a rather flat topography (E*). The same polish characteristics were observed on our experimental dry hide processing tools (D**). Some of the polish on the mesial part of the same ventral distal edge has a rough and smooth texture. On some points, the polish is associated with fine striations running perpendicular to the edge, which indicate a transversal use-direction. According to the wear characteristics, the left edge was likely used in a transversal negative motion with a high working angle (close to 90º). The well-developed polish found on the ventral distal edge, its characteristics, and its association with rough polish on some areas of the edge, together with its location restricted to the outer edge, enable us to affirm that it was likely produced during a scraping activity in soft-medium material, very likely dry hide.

The fact that hafting traces were identified on the artifact indicate that it was possibly used as a hide-stripping knife. Nonetheless, the presence of a step-terminating bending fracture on the ventral distal tip suggests that the artifact was subjected to other processes, for which projectile use, butchery or even trampling are possible. The right edge is slighly affected by patina on the distal extremity, what prevented correct identifications of use-wear traces. Small scars on the mesial dorsal right edge were recorded at the same level that the hafting polish identified on the dorsal ridges, supporting the hafting interpretation of the artifact.

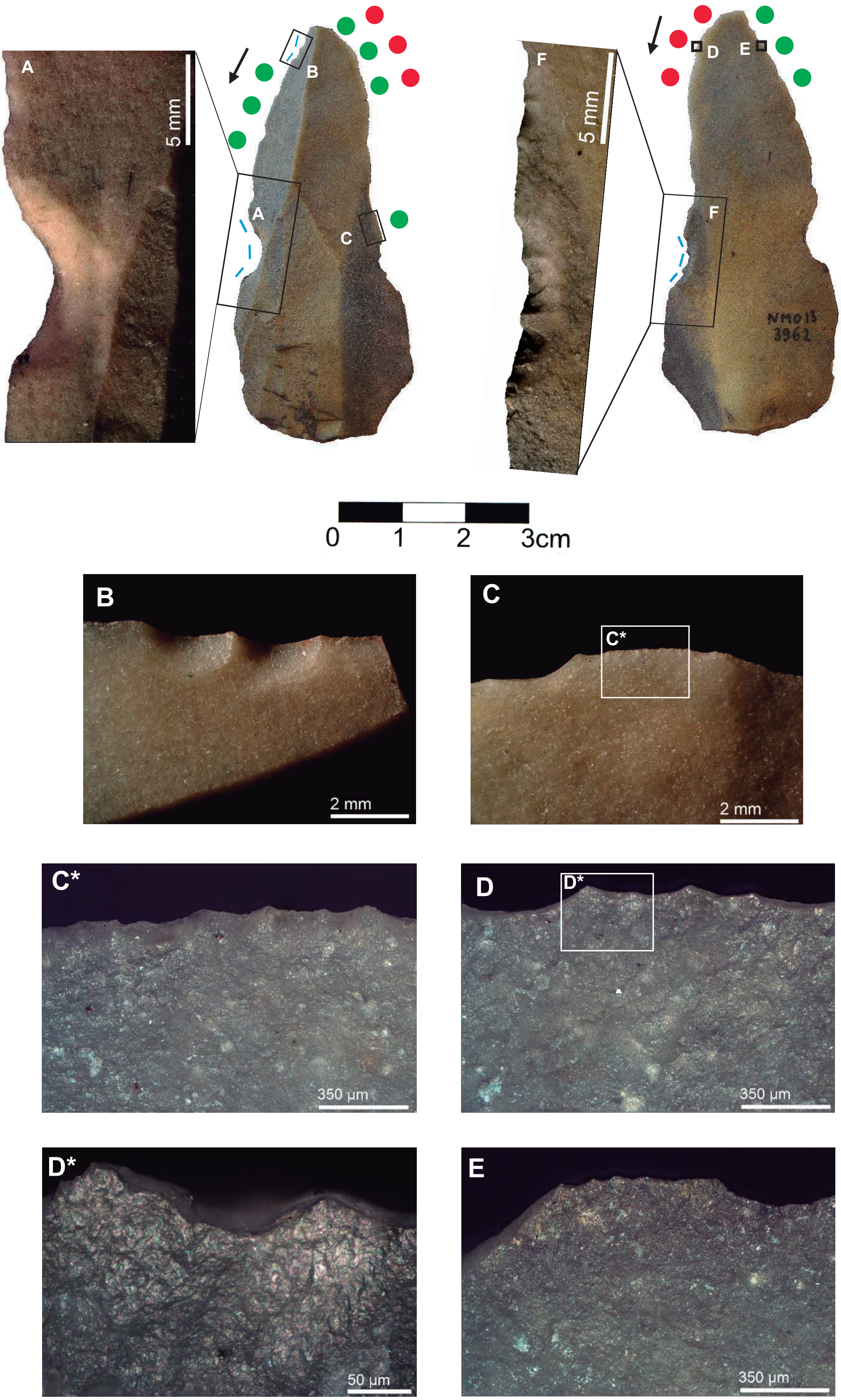
A
5 mm
B
C
F
D
E
NMO15
3962
0
1
2
3cm
B
2 mm
C
C*
2 mm
C*
350 µm
D
D*
350 µm
D*
50 µm
E
350 µm

Supplementary figure S6. Non-Levallois point NMO13 3962, typologically classified as notch, showing use-wear and indirect evidence of hafting on both lateral edges. A, notch-denticular retouch on the dorsal mesial left edge, 35x, HFOV (2cm); B, regular retouch on the distal dorsal left edge associated with a distal fracture, 35 x, HFOV 8.6 mm; C, shallow scalar scars on the mesial dorsal right edge, 100x, HFOV 8.6 mm; C*, close-up of the previous scars, 100x; D, sliced scars associated with weak polish on the ventral distal right edge, 100x; D* close-up of the previous image in which the polish is cut by a scar removal, 500x; E, weak polish and micro-scars on the ventral distal left edge, 100x; F, Notch retouch on the ventral mesial right edge, 35 x, HFOV 1cm. *Blue dashed lines indicate intentional notches (A, F) and regular retouch (B).

Interpretation

Weak polish has been identified on the ventral distal right and left edges associated with sliced scars (D, E), indicating a longitudinal motion in a soft animal material in both cases. Regular retouch was identified on the dorsal distal left edge near the distal fracture (B). Very shallow scalar scars with feather termination were identified on the dorsal mesial right edge, suggesting a possible contact with bindings around the haft limit (C). The presence of notch-denticular retouch on the dorsal mesial left (A) and right edges (F) suggests that the point was likely hafted. The notch retouch on both lateral edges was intentionally produced for the placing of bindings to secure the tool against the handle and to prevent these bindings from being cut by the sharp edges during use. The combination of wear patterns on the tool indicates that the tool was used as a hafted butchering knife. The retouch on the distal dorsal left edge could have been intentionally produced to reinforce the narrow distal extremity of the tool. The appreciable distal break was likely produced during butchery.

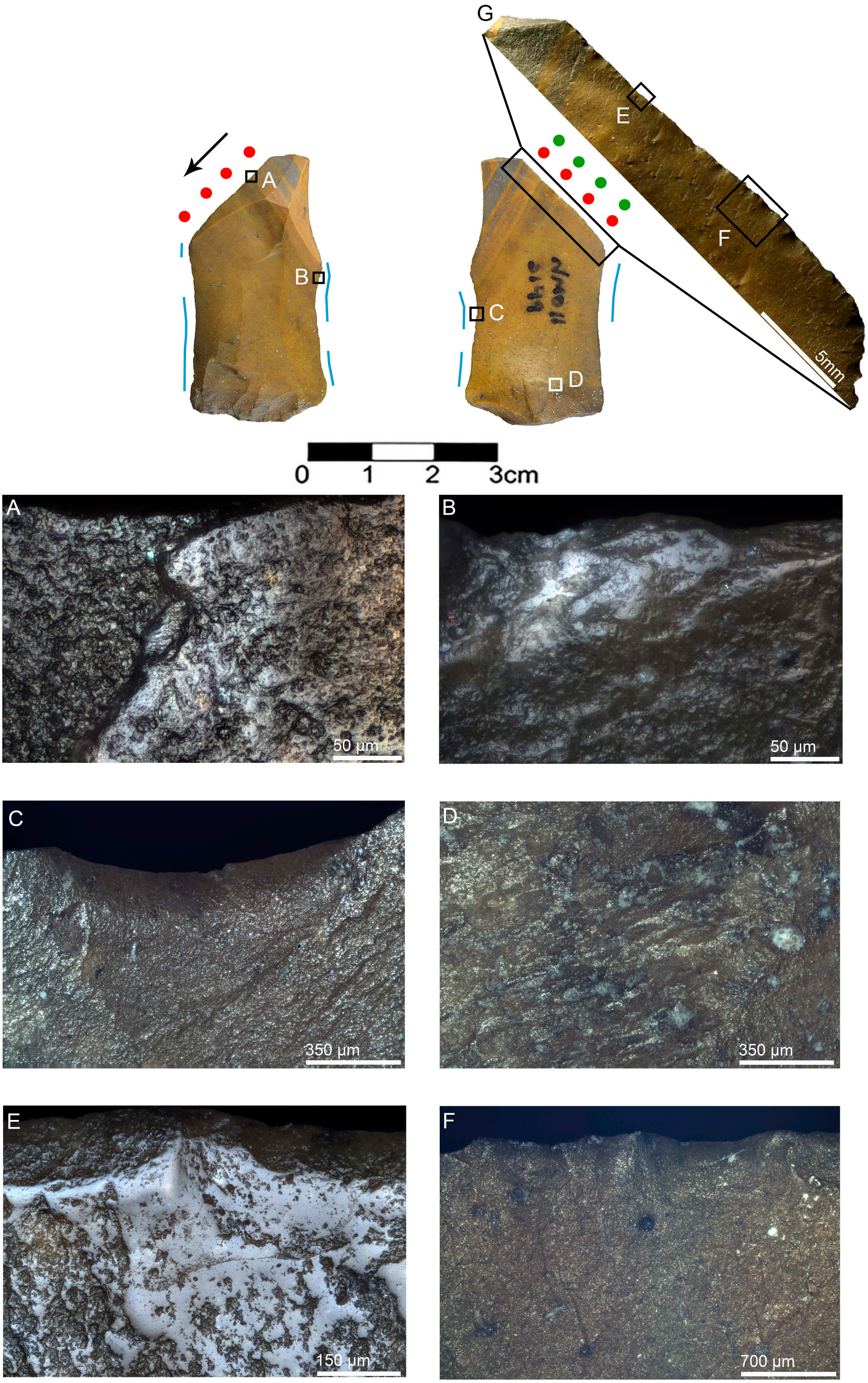

Supplementary figure S7. Non-Levallois point NMO 11 3149 showing use-wear on the distal edge and hafting wear on both lateral edges. A, rough-bright polish on the dorsal distal edge, 500x; B, hafting bright spot on the dorsal mesial right edge, 500x; C, hafting scar on the ventral mesial left edge, 100x; D, hafting polish on the ventral bulbar area, 100x; E, Plant polish and edge scarring on the ventral distal left edge, 200x; F, use-scars on the ventral distal left edge, 50x; G, map of the ventral distal left edge showing

the distribution of scars, 35x. *Blue solid lines indicate the areas where hafting traces are located.

Interpretation

Well-developed rough and bright polish was identified on the dorsal left lateral edge (A). On the ventral side of the same edge, well-developed smooth polish with domed and flat topography (E) was observed associated with feather and step-terminating scars (F). The polish characteristics indicate that soft plants were likely processed with this artifact. The presence of feather and step-terminating scalar scars on the ventral distal left edge, with some of them showing an oblique orientation (F), and associated with polish, seems to indicate that this point was used for cutting plants, likely reeds. Brigh spots were identified on the mesial dorsal right edge (B) together with hafting scars on the ventral side of the same edge (C).The hafting scar located on the ventral mesial right edge (sliced into scalar scar) suggests that the hafting arrangement of the artifact included the application of bindings. Possible hafting polish was observed on the high parts of the topography in the ventral bulbar area. Possible basal thinning scars are visible on the dorsal proximal extremity of the artifact. The combination of wear features indicates that this point was likely used as a hafted plant-cutting knife, likely for processing reed plants.

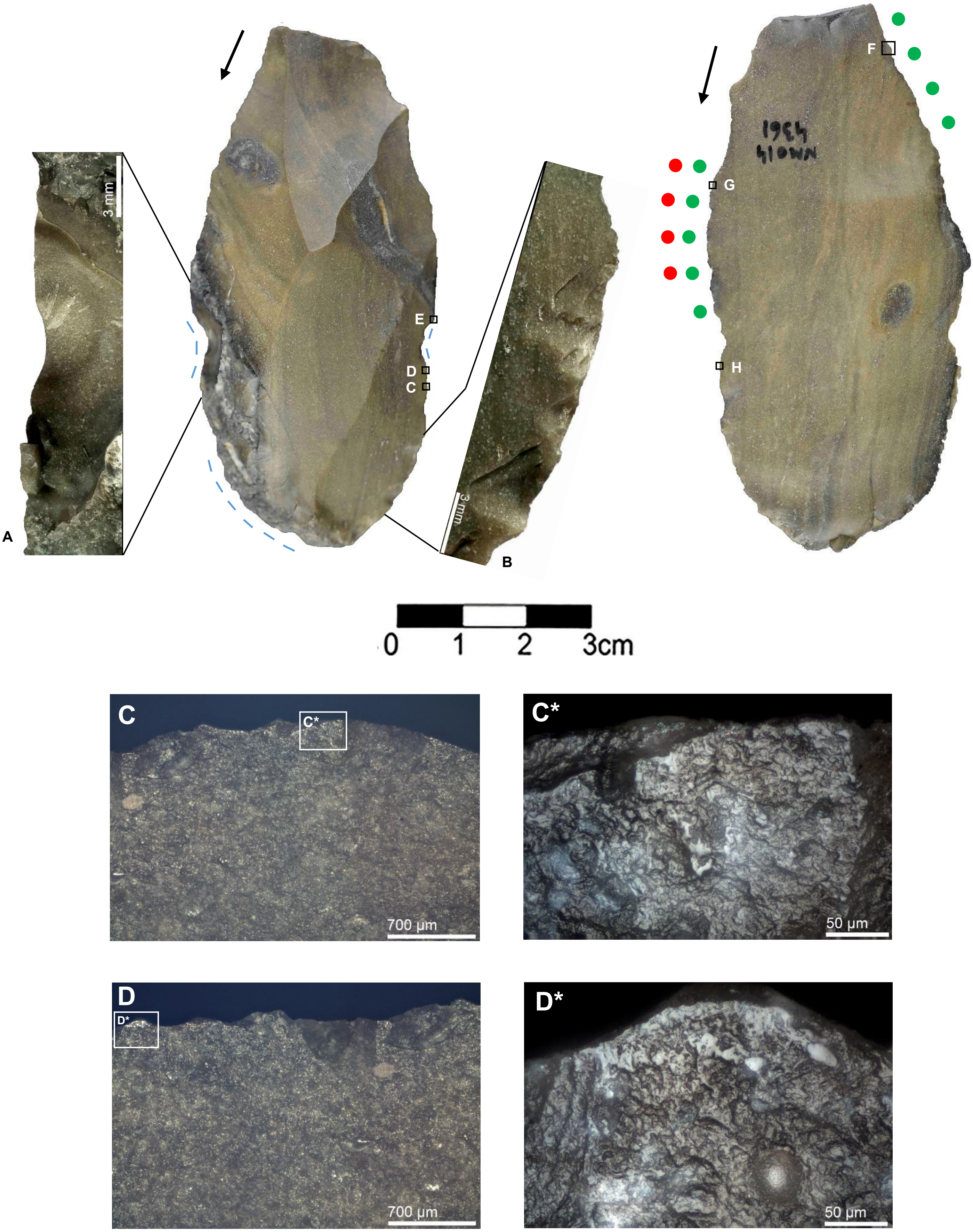
A
3 mm
E
D
C
B
3 mm
F
G
H
0 1 2 3cm
C
C*
700 µm
C*
50 µm
D
D*
700 µm
D*
50 µm

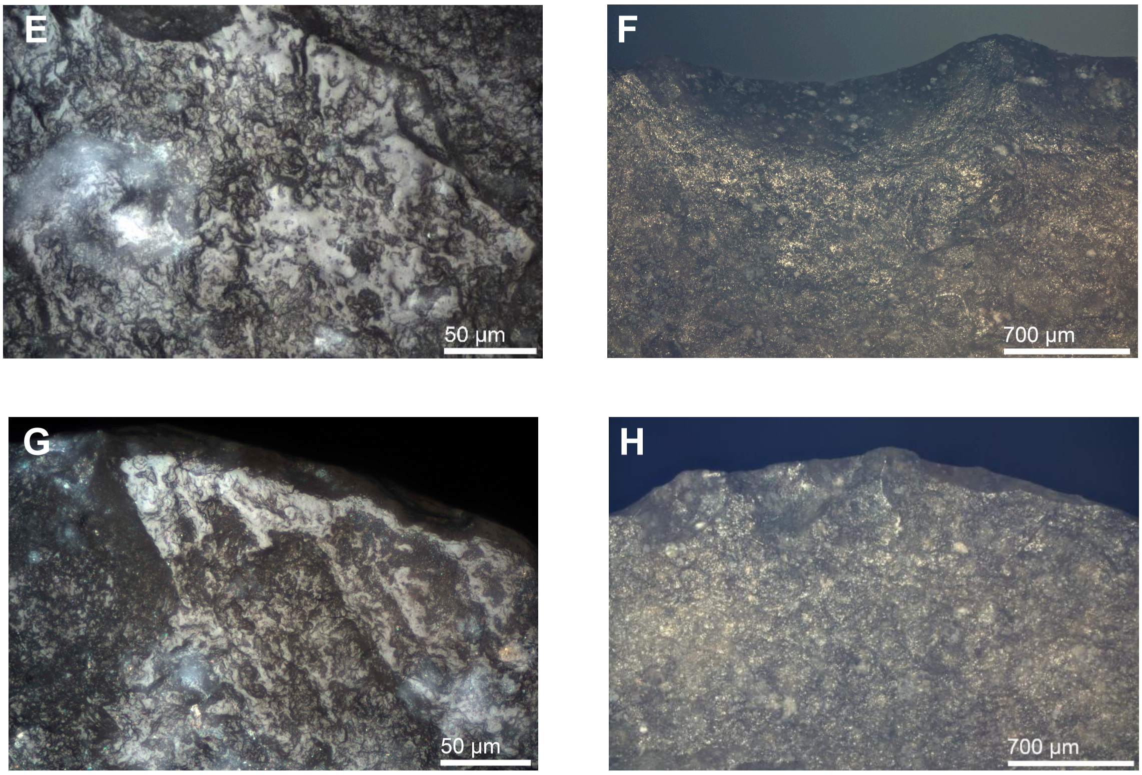

Supplementary figure S8. Naturally backed knife NMO14 4361 showing use-wear and hafting wear on both lateral edges. A, *notch-denticular retouch on the mesial dorsal right edge, 35x, HFOV 2cm; B, grouping of step-terminating trapezoidal scars on the dorsal distal left edge showing overlapping and crushed initiations, 35x, HFOV 2cm; C, step-terminating micro-scars and polish on the ridges and prominent parts of the dorsal distal left edge, 50x; C*, close-up of the previous image depicting the rough and smooth texture of the hafting polish and micro-scarring, 500x; D, step-terminating hafting scars associated with hafting polish, 50x; D*, Detail of the hafting polish showing smooth texture and wavy topography, 500x; E, well-developed hafting bright spots and polish on the dorsal mesial left edge outside the notch retouch in an area where the haft boundary probably was, 500x; F, bending scars on the dorsal proximal right edge, 50x; G, invasive rough polish on the dorsal proximal left edge, 500x; H, grouping of hafting scars on the ventral distal left edge located at the same height that the hafting wear depicted on the opposite dorsal face, 50x. *The tool is depicted upside-down to facilitate understanding of hafting. *Blue dashed lines indicate intentional notches in the mesial part of both lateral edges (e.g., A) and abrupt retouch in the distal right back of the tool.

Interpretation

The tool was likely hafted and used upside down (not following technical orientation). The distal dorsal extremity was retouched on the right side (bottom left on the image), as opposed to the dorsal distal left side of the tool, which shows step-terminating scars with crushing on the initiations (B). Notch-denticular retouch is visible on both lateral edges on the dorsal face at the same height (A), indicating the location of the haft boundary. Hafting polish, bright spots, and scarring are concentrated near these notches (C, D, E, H). An association of well-developed rough polish and bending initiated and feather terminated scars were found on the proximal part of both lateral edges on both faces of the tool (F, G). These wear features are very similar to the wear traces observed in some of our experimental tools used in butchery, which suggests that this tool was probably used for butchery.

The presence of the notches on the mesial part of the edges indicate the use of bindings to secure the tool into the haft. The presence of hafting wear on both faces of the tool and its

characteristics and distribution suggest that the tool may have been hafted in a male hafting arrangement [1-3], opening the possibility to the use of animal materials for hafting (e.g., bone or antler [4,5], and horn). The hafting scarring shown on the dorsal distal left edge indicate that the tool was used in a high-pressure activity. These scars together with the well-developed hafting micro-wear, contrast with the absence of well-developed use-wear traces on the active parts of the artifact, with the exception of the polish and edge rounding shown on G. The fact that hafting and use-wear traces do not match in intensity suggests that the most developed use-wear traces may have been removed by resharpening. This would explain the intensity of the hafting scarring and polish observed on the distal left lateral edge.

References

1. Stordeur, D. La Main et l'Outil. Manches et emmanchements préhistoriques. Lyon Maison l'Orient Méditerranéen (1987).

2. Rots, V. Hafting Traces on Flint Tools: Possibilities and Limitations of Macro- and Microscopic Approaches. Katholieke Universiteit Leuven (2002b).

3. Rots, V. Prehension and hafting traces on flint tools: a methodology. Leuven University Press (2010).

4. Rots, V. Are Tangs Morphological Adaptations in View of Hafting? Macro- and microscopic wear analysis on a selection of tanged burins from Maisières-Canal. Notae Praehistoricae **22,** 61–69. (2002a)

5. Rots, V. Wear traces and the interpretation of stone tools. J. F. Archaeol. **30,** 61–73 (2005).

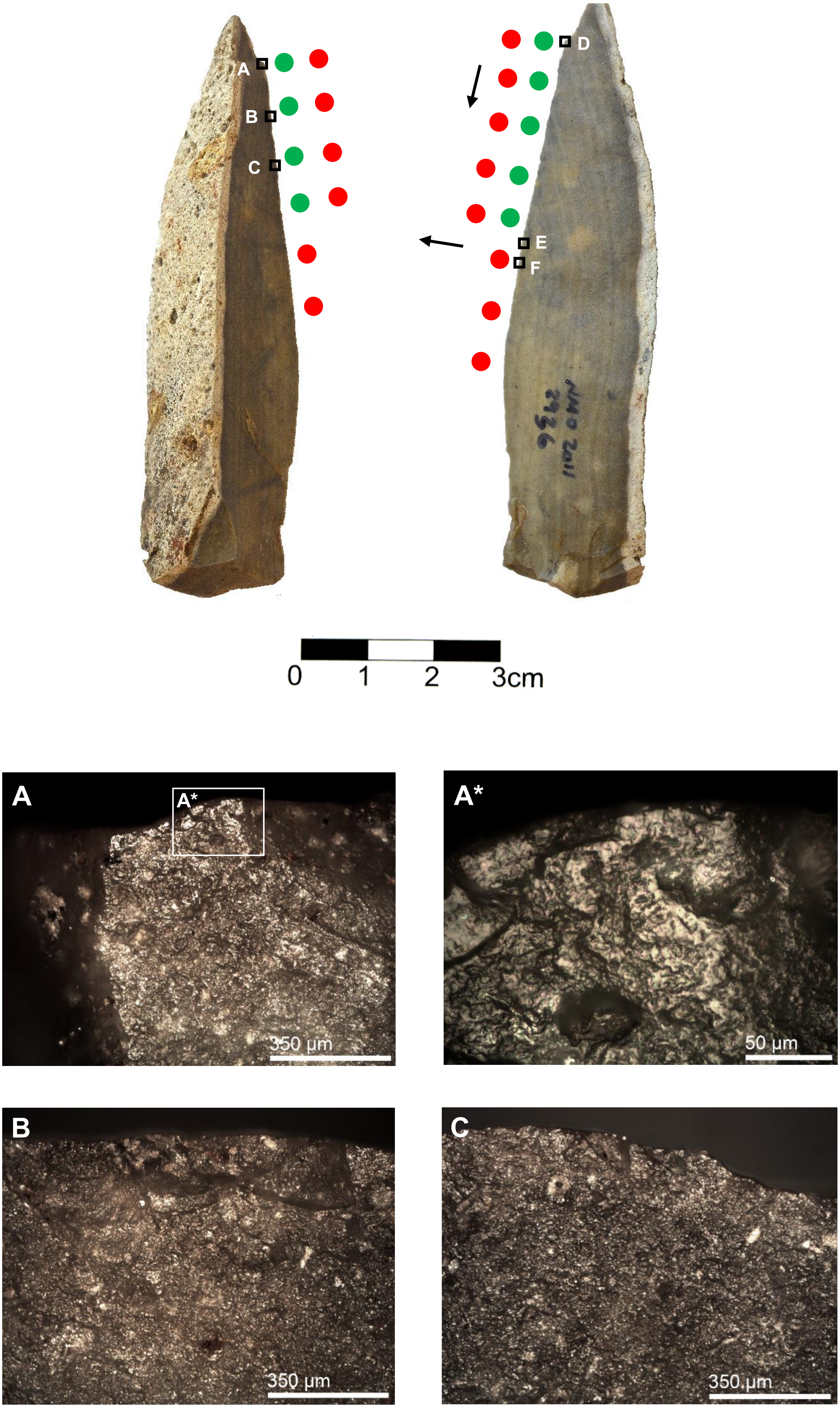
A
B
C
D
E
F
0
1
2
3cm
A
A*
350 µm
A*
50 µm
B
350 µm
C
350 µm

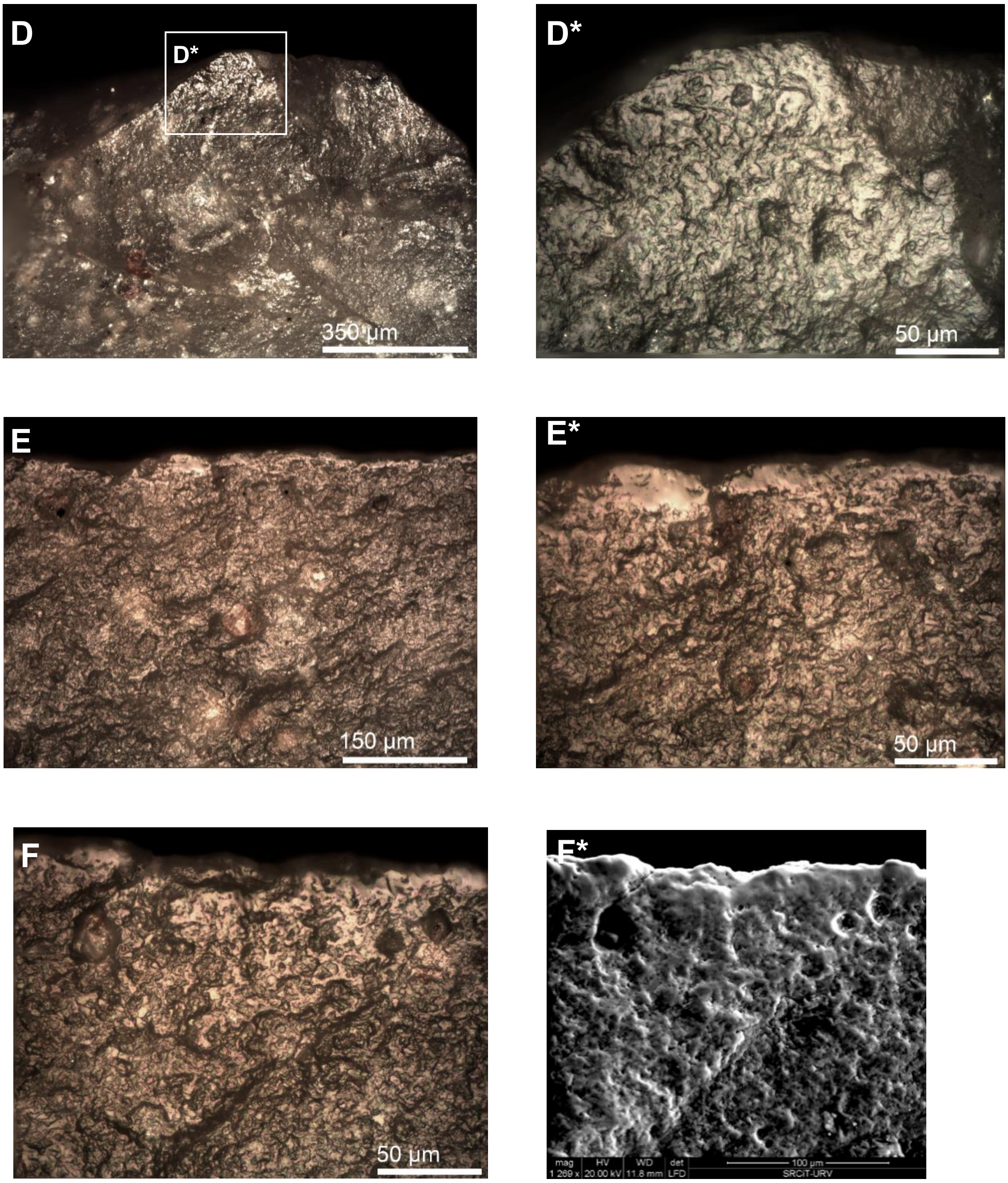

Supplementary figure S9. Naturally backed knife NMO11 2936 showing use-wear on the right edge. A, smooth polish on an elevated area between scars, 100x, HFOV 1130 µm; A*, close-up of the polish showed in the previous image, 500x, HFOV 226 µm; B, overlapping step-terminating scars on the distal dorsal edge, 100x, HFOV 1130 µm; C, step-terminating micro-scars with oblique orientation, 100x, HFOV 1130 µm; D, edge scarring and polish on a prominent area of the ventral distal edge, 100x, HFOV 1130 µm; D* Close-up of the previous image in which the rough, dull polish is visible, as well as edge rounding and parallel fine striations, 500x, HFOV 226 µm; E, E*, smooth flat polish and transversal ripples on the mesial ventral edge, 200x, 500x, HFOV 564 µm, 226 µm; F, smooth flat and wavy polish on the ventral mesial edge, 500x, HFOV 226 µm; F*, the previous image observed with SEM in which the polish texture is better appreciated, as well as the areas with poor polish development further inside.

Interpretation

A polish band restricted to the outer edge was observed on the mesial ventral right edge, showing a smooth texture and flat-wavy topography (E*, F, F*). This polish is very restricted to the outer edge, indicating that hard material was processed, likely bone. Perpendicular fine striations and ripples are visible on these polish bevels at high magnifications, supporting the interpretation for a transversal use-motion (E*). The location of this polish on the distal part of the mesial ventral edge contrast with the presence of butchery traces on the distal edge near the tip. Rough, dull polish and edge rounding and longitudinal striations are visible on the most prominent part of the distal ventral edge (D*). It can be appreciated that the polish is associated with overlapping step-terminating scars (D). These wear patterns indicate that the distal extremity was the first part of the edge to contact the worked material, particularly soft animal tissue during a butchery activity. The presence of two different micro-polishes and distribution indicate that its user took advantage of the abrupt edge angle and the large back of the tool to use it for different activities (involving different use-motions and worked materials) linked with butchery. The presence of rough and smooth polish on the dorsal distal edge (on the opposite side of picture D) in an elevated area between scars (A, A*) support the butchering knife interpretation of the tool, which also included bone-scraping.

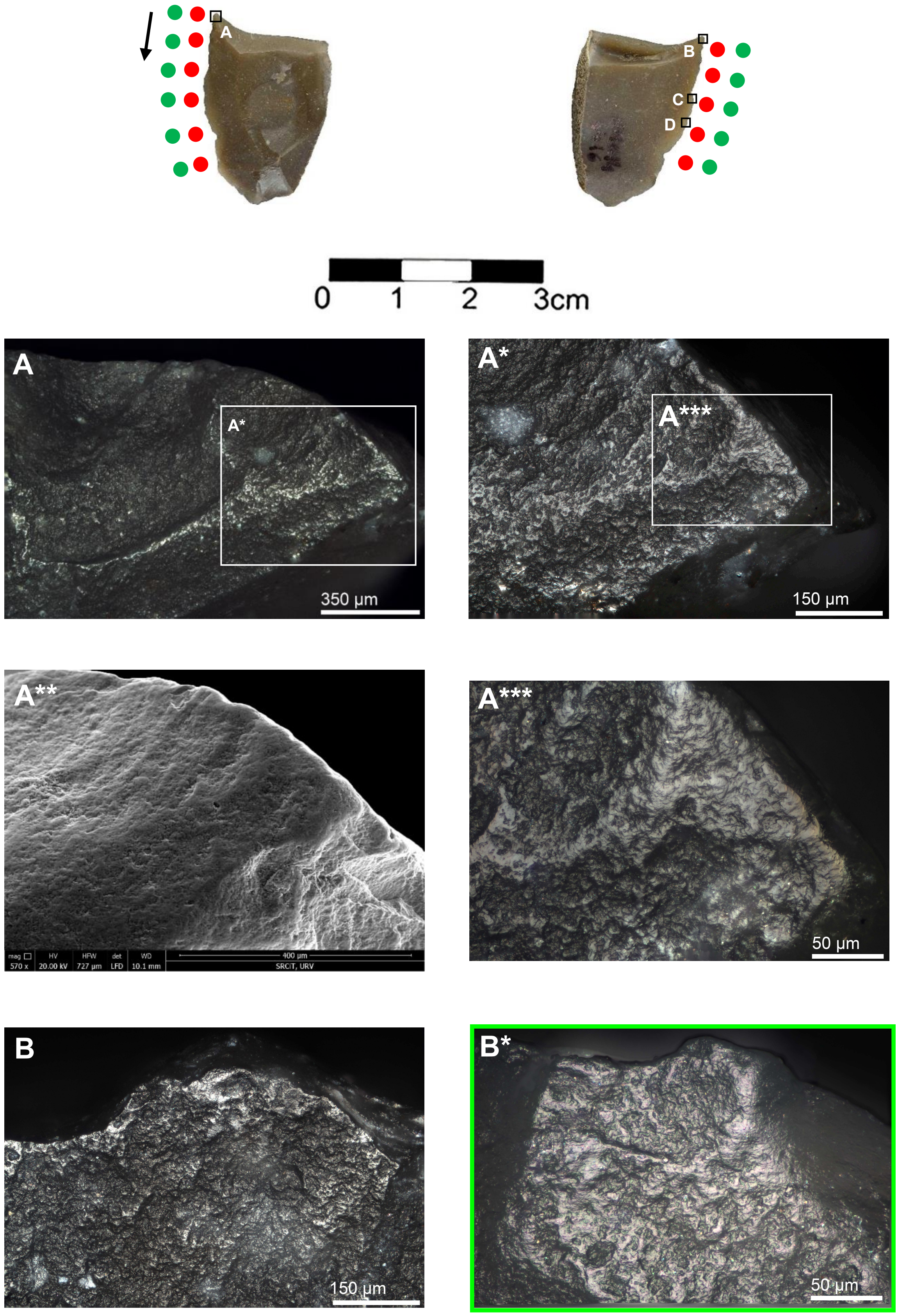
A
B
C
D
0 1 2 3cm
A
A*
350 µm
A*
A***
150 µm
A**
400 µm
A***
50 µm
B
150 µm
B*
50 µm

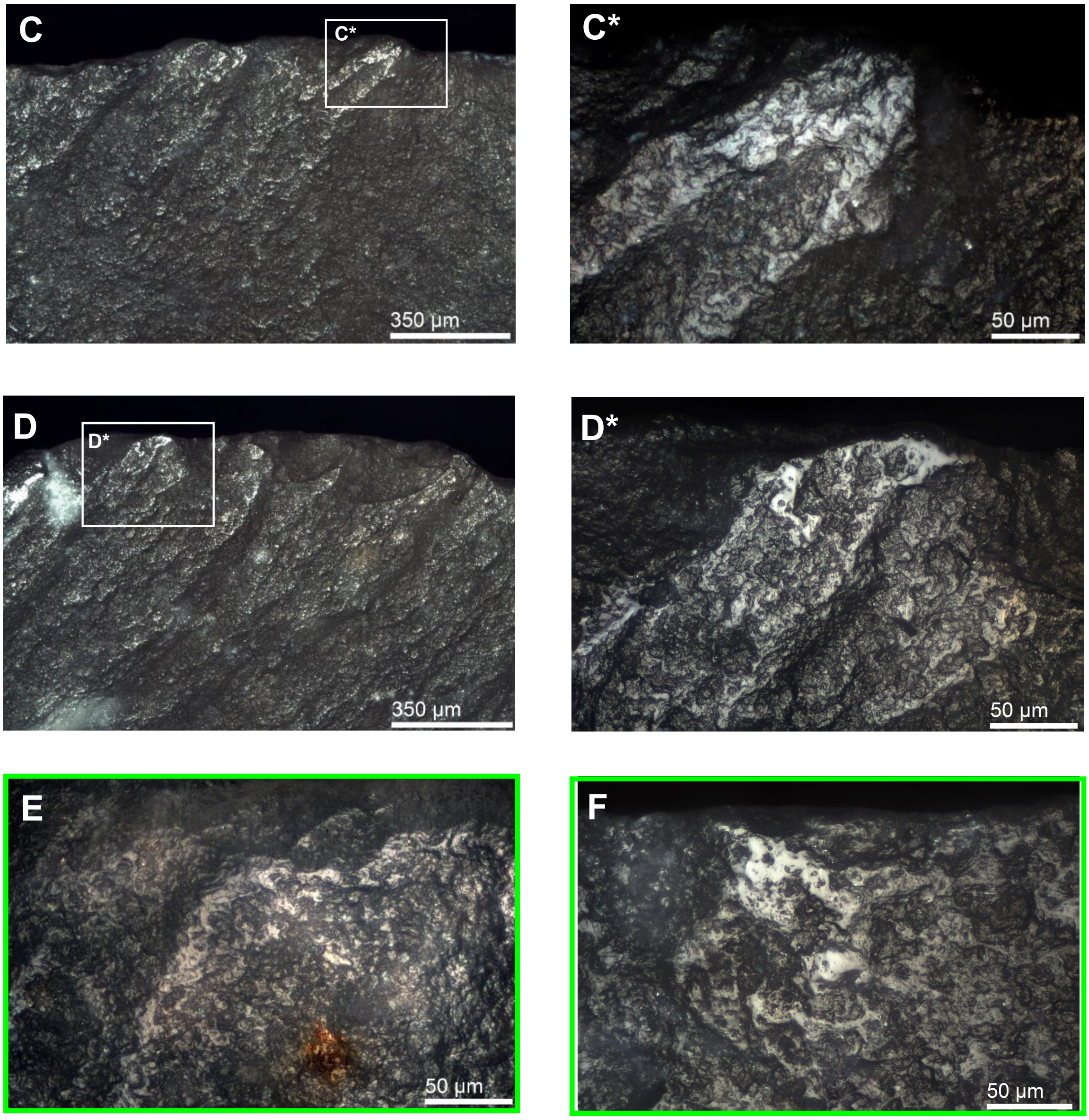

Supplementary figure S10. Naturally backed knife NMO16 4416 showing use-wear on the left lateral edge. A, A*, A***, hinge-terminated scar, rough-dull polish, and edge rounding on the dorsal distal tip of the left edge, 100x, 200x, 500x; A**, picture A* observed with SEM showing well developed polish, edge rounding, and few scars; B, smooth-rough polish and edge rounding on the distal ventral tip of the left edge, 200x; B*, experimental rough-dull polish and edge rounding from cutting fresh deer hide (*Cervus elaphus*), 500x, 2800 strokes, 45 min; C, C*, invasive smooth-rough polish, micro-scars and edge rounding on the ventral mesial left edge, 100x, 500x; D, D*, step and feather terminated scars associated with smooth polish and rough-dull polish on the ventral mesial left edge, 100x, 500x; E, experimental rough-dull polish from cutting fresh deer hide (*Cervus elaphus*), 500x, 1350 strokes, 20 min; F, experimental smooth and rough-dull polish from cutting dry hide, 500x, 3172 strokes, 45 min.

Interpretation

Both sides of the left lateral edge present rough-dull polish, edge rounding and feather and step/hinge-terminated scalar scars (A, A*, A***, D). The polish and edge rounding are appreciable with scanning electron microscopy (A**). This polish is more developed in the elevated areas and prominent parts of the edge (A, C, C*, D, D*). The polish is invasive on both faces of the edge (C, D), but in some areas, it is restricted to the very edge (B). In many edge areas, the polish presents different development stages. However, most of the time, the smooth polish is restricted to the edge and high parts of topography between scars (C*, D*), whereas the rough-dull polish is located on the edge and further inside (A*, C, D). These wear patterns were observed in our experimental hide-cutting tools (B*, E, F), what suggest that this NBK was used for cutting hide, possibly in a fresh state with somewhat dry parts.

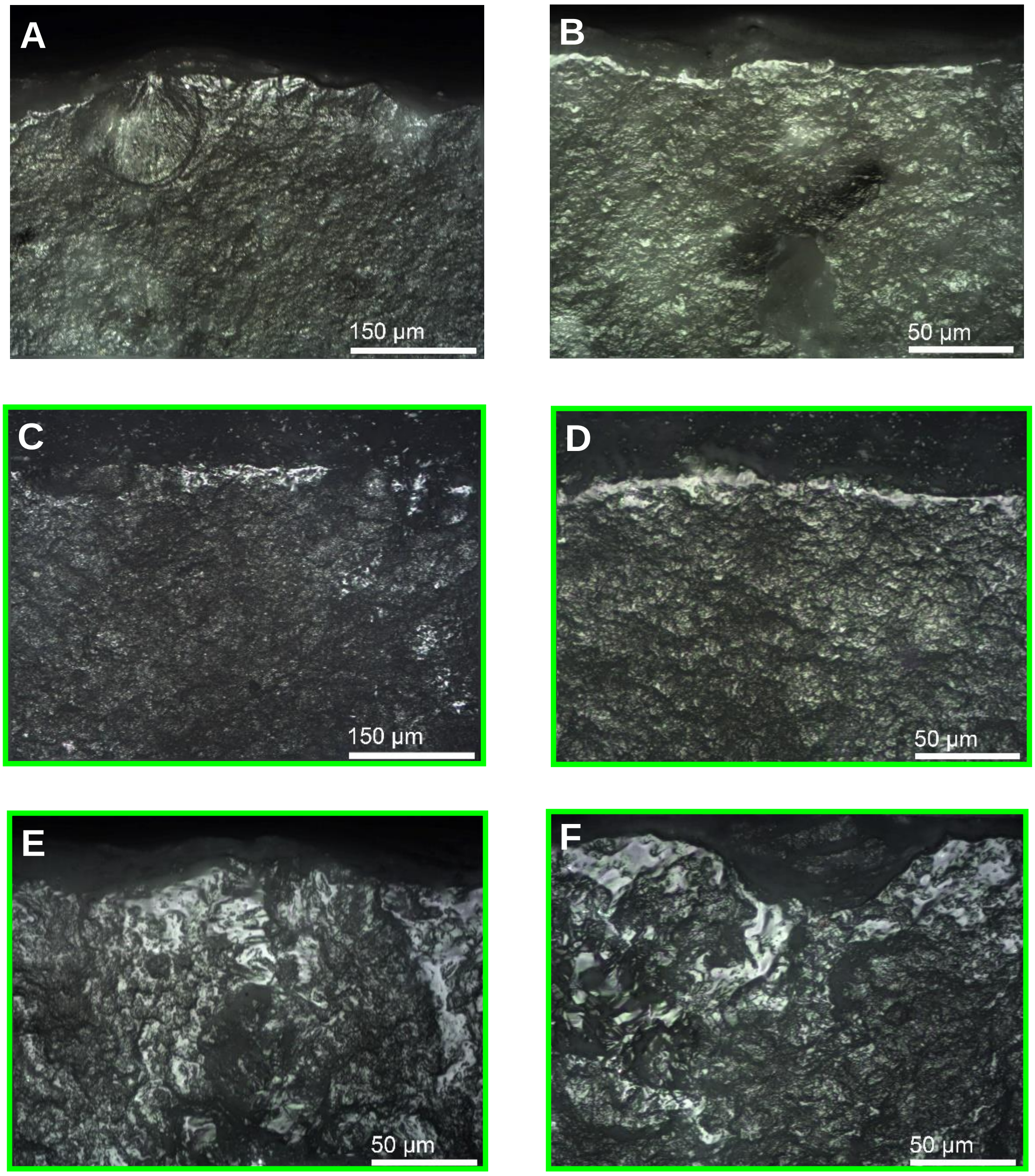

Supplementary figure S11. Comparison of different archaeological and experimental wood-processing wear. A, B,. Smooth-wavy polish and edge rounding on the dorsal distal right edge of artifact NMO14 4415, interpreted as wood-whittling tool, 200x; C, D, E, F, Different polishes documented in a experimental tool used for sawing dry wood, which includes smooth-wavy polish rectricted to the edge (D), and invasive smooth-wavy polish associated with edge rounding and longitudinal ripples (E, F); C, D, 200x; E, F, 500x; 3000 strokes, 30 min.

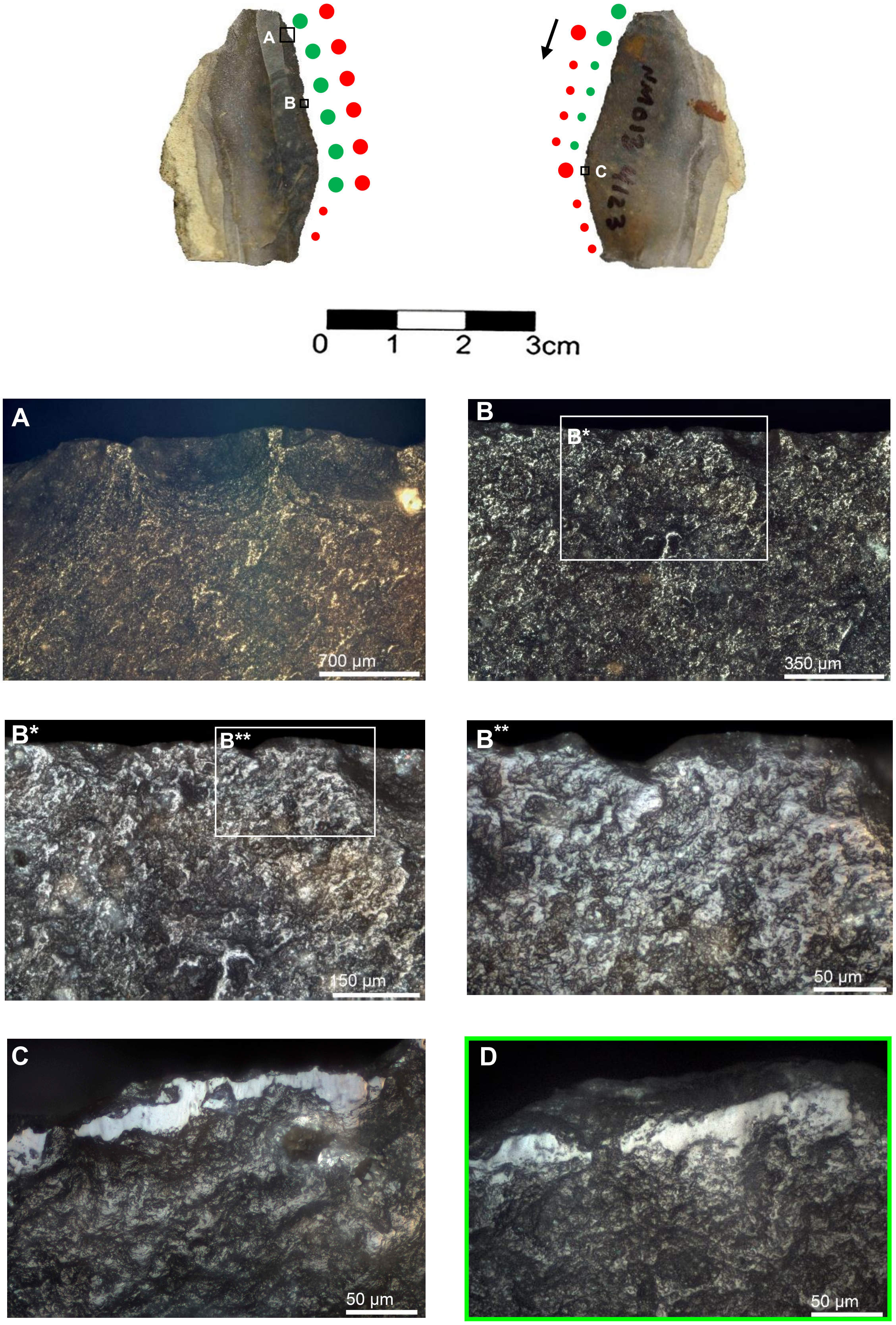
A
B
C
0 1 2 3cm
A
700 µm
B
B*
350 µm
B*
B**
150 µm
B**
50 µm
C
50 µm
D
50 µm

Supplementary figure S12. Use-wear on the right lateral edge of unretouched cortical flake NMO13 4123. A, scalar scars with feather/step termination and polish on the elevated areas of topography and scar ridges, 50x; B, feather and step-terminating micro-scars with oblique orientation and associated with invasive polish, 100x; B*, close-up of the previous image in which the rough texture and dull appearance of polish are appreciable together with a low degree of linkage, 200x; B**, detail of the polish showed in B* in which the linkage and rough texture of polish are better appreciated together with edge rounding, 500x; C, smooth polish, possibly cut by resharpening, showing flat and micro-wavy topography and weak rough-dull polish further inside, 500x; D, Smooth polish showing a mixture of flat and micro-wavy topography and associated with rough-dull polish and edge rounding on the right ventral edge of the experimental tool NMO-EXP25-P used for scraping dry deer hide, 500x, 2817 strokes, 45 minutes.

Interpretation

A well-developed pattern of scalar scars with feather termination is visible on the distal and mesial extremities of the right dorsal edge. A rough-dull polish with a low degree of linkage is visible on the scar ridges and high parts of topography. The polish presents rough-dull and gritty texture, and is associated with light edge rounding (B**). The polish is not invasive and developed on the ventral face, though well-developed smooth polish was identified on the mesial edge (C). This smooth flat polish with microwaves is very similar to the polish identified in our experimental dry-hide processing tools (cutting and scraping), although, contrary to our experimental pieces, the absence of edge rounding indicates that the polish showed in (C) may have been cut by resharpening. In our experimental tools, the smooth polish is surrounded by rough-gritty polish (D) most of the time. In tool NMO13 4123, weak, rough polish is also present further inside next to the smooth polish (C), suggesting that dry hide was likely processed. Some fractures and scarring are present on the distal dorsal and ventral side of the edge, maybe due to contact with the most dehydrated/dried parts of the hide, such as the dermis in a dry state. These scars consist of feather terminated scalar scars grouped in ordered patterns (A). All the characteristics indicate that this tool was used for cutting dry hide.

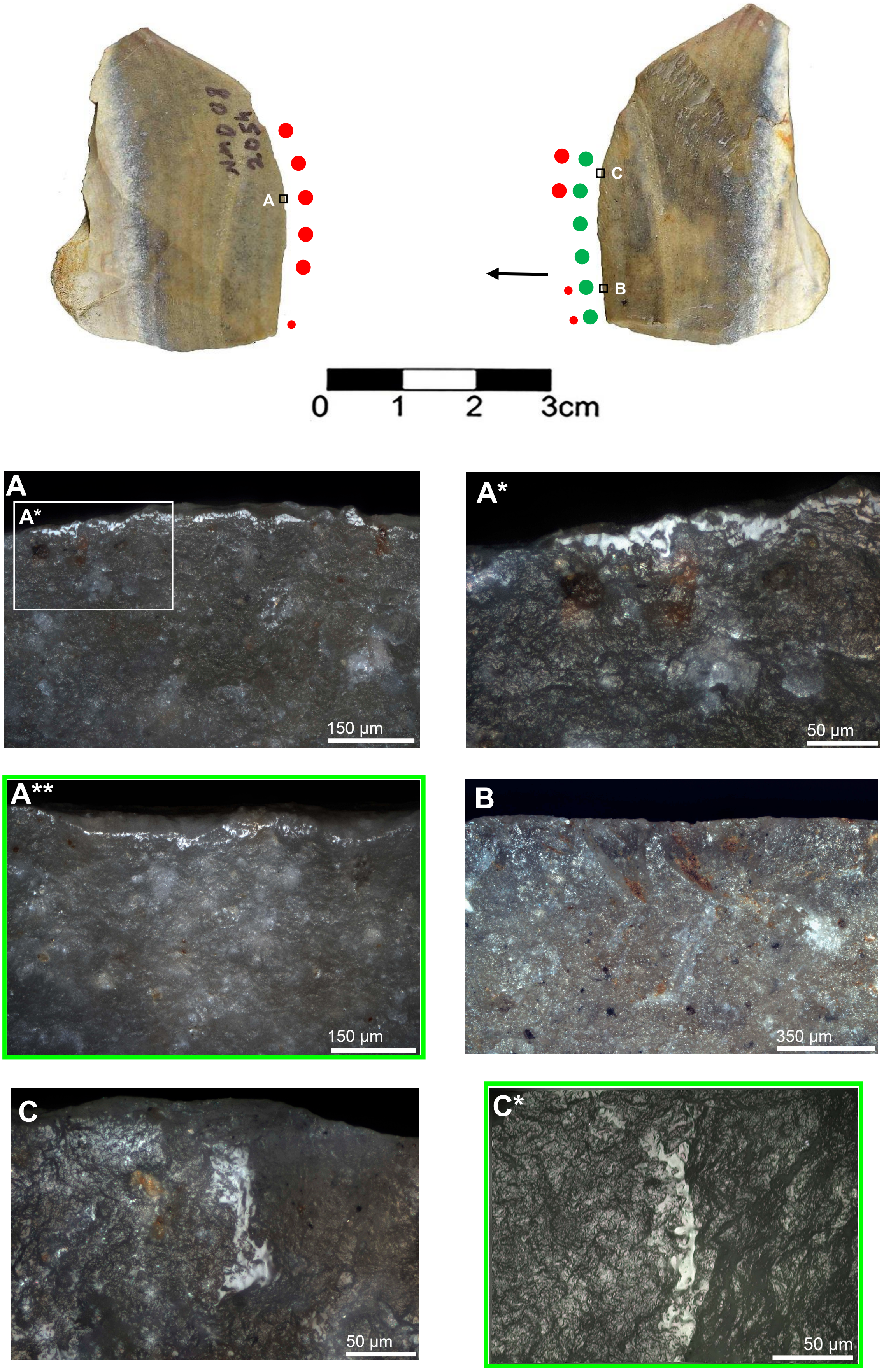

Supplementary figure S13. Use-wear on the right edge of unretouched flake NMO08 2054. A, A*. smooth-wavy polish and transversal ripples on the mesial dorsal right edge, 200x, 500x; A**, experimental smooth-wavy polish and transversal ripples from scraping dry soft wood (*Salix, s.p.*), 2552 strokes, 40 min. B, step scars showing a lightly oblique orientation and weak polish on the elevated areas on the ventral proximal right edge, 100x; C, smooth-wavy polish between scars on the ventral mesial part of the edge, 500x; C*, experimental smooth-wavy polish from sawing dry wood (*Pinus sylvestris*), 3000 Strokes, 30 min. Black arrow indicate direction of work.

Interpretation

The dorsal edge presents a well-developed pattern of smooth-wavy polish spots and bands. Edge rounding is also present on the mesial part of the edge associated with micro scars and smooth polish band (A). Transversal ripples are visible on the polish, indicating tool-use direction. The texture of the polish is smooth with flat-wavy topography, suggesting that soft-medium material was processed, likely wood in a transversal positive motion. These wear features were also observed on our experimental tools used for scraping dry wood (A**). There is no evidence of wear traces on the distal part of the edge. The ventral edge presents a well-developed pattern of feather/step scalar and trapezoidal scars associated with less developed polish in the high parts of topography (B) on the proximal portion of the edge. On the mesial part of the edge, there is well-developed smooth polish associated with micro-chipping and light edge rounding (C). The polish texture is the same as the polish observed on the dorsal face, but is more invasive in this area of the edge. These features suggest that this tool was used in a transversal positive use-motion, likely for whittling wood. Interestingly, the same polish texture and distribution was observed on our experimental tools used for sawing dry wood (C*).

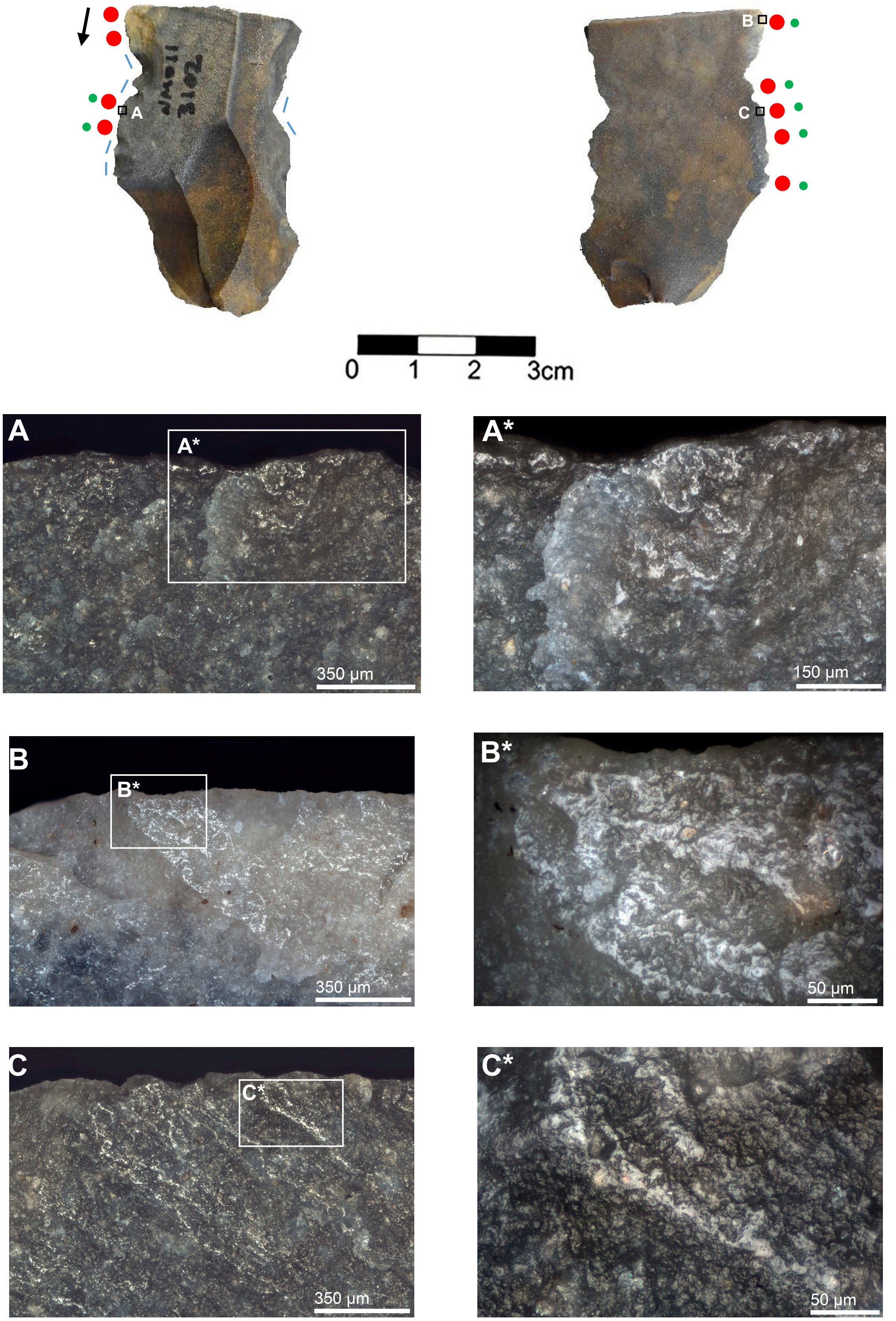

Supplementary figure S14. Denticulate NMO11 3102 showing use-wear on the left lateral edge. A, A*, rough-dull polish and light edge rounding on the dorsal mesial left edge, 100x, 200x; B, feather scalar scars and rough-dull polish on the ventral distal left edge, 100x; B*, close-up of the rough-dull polish showed in the previous image in which some linkage is visible, 500x; C, micro-scars and invasive polish on the ventral mesial left edge. Note that the polish follows the ridges and high parts

of topography, 100x; C*, close-up of the previous image, showing well-developed rough-dull polish on a small ridge, 500x. *Blue dashed lines indicate retouch.

Interpretation

A rough, dull polish is present on the distal and mesial left dorsal and ventral edge. The polish is located on the prominent parts of the edge, away from the concavities left by the retouch. This polish is associated with feather terminated scalar scars on both faces of the tool (B). The polish is more invasive on the ventral face (C) than on the dorsal side (A). It follows the lancets and high parts of topography. We suggest that this tool was used for cutting soft animal material, likely fresh hide. Furthermore, there are no visual traces on the proximal part of the edge, indicating that the tool was probably used with distal-proximal grip, taking advantage of the abrupt distal fracture to place the fingers.

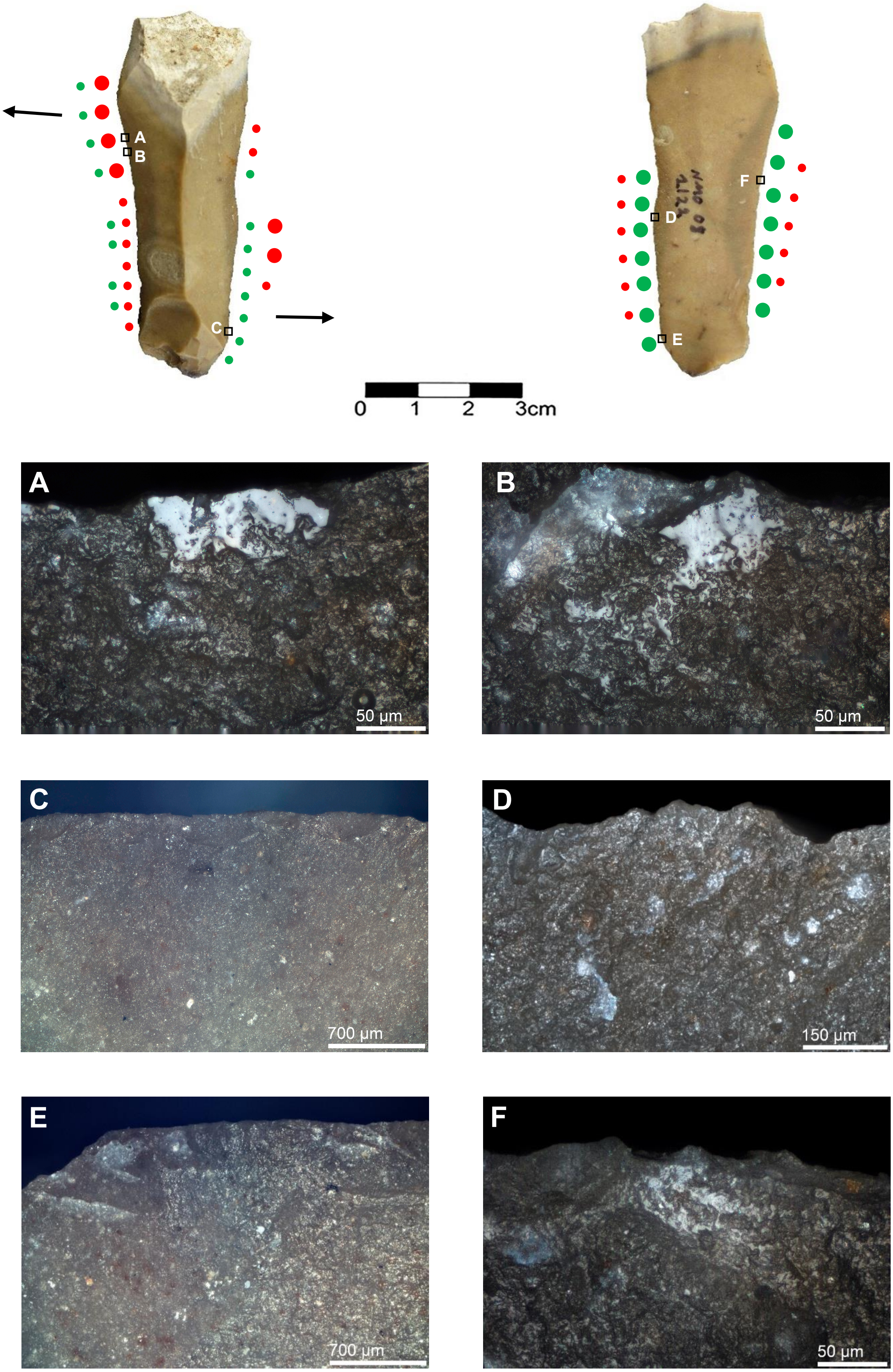
A
B
C
D
E
F
0 1 2 3cm
A
50 µm
B
50 µm
C
700 µm
D
150 µm
E
700 µm
F
50 µm

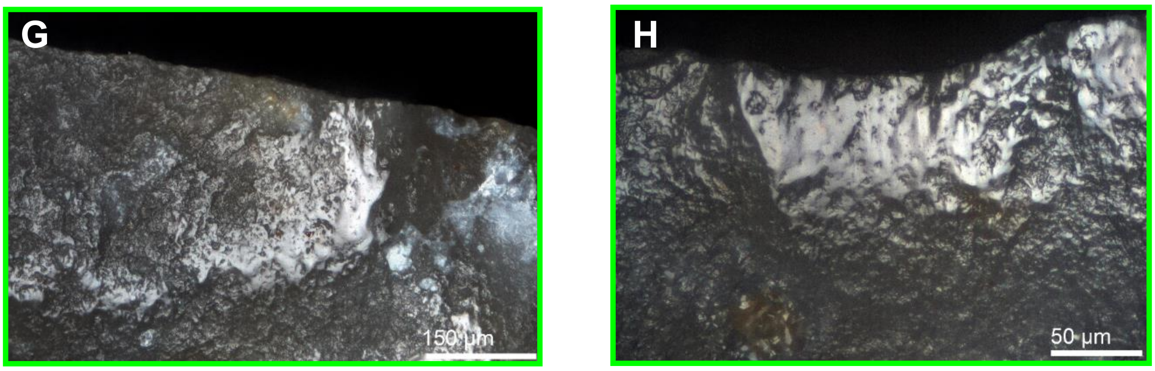

Supplementary figure S15. Unretouched cortical blade NMO08 2122 showing use-wear on both lateral edges. A, B, smooth-pitted polish showing flat-wavy topography; C, step-terminating trapezoidal scars on the dorsal right proximal edge, 50x; D, weak polish on the ventral left mesial edge, 200x; E, step/feather terminating scars on the ventral proximal right edge, 50x; F, rough polish showing transversal striations on the ventral left mesial edge, 500 x; G, experimental plant polish from whittling fresh erect bamboo (sp.) with smooth-pitted texture and wavy topography on tool NMO-EXP12-V, 200 x, 2000 strokes, 35 min; H, experimental plant polish observed on the same tool in a different location, 500x.

Interpretation

The Dorsal left edge presents a well-developed pattern of smooth-pitted polish with a mixture of flat and wavy topography on the distal extremity (A, B). These features were also observed in our experimental plant processing tools (G). The polish is not developed in mesial and proximal extremities. On the contrary, polish is barely present on the ventral side of the edge, where only rough, weak polish is present on scar ridges and prominent parts, sometimes showing transversal striations (F). This polish is associated with feather scalar and step trapezoidal scars. These wear features indicate that the left edge was used for whittling medium-hard wood or plants. Groupings of scars are larger and more frequent on the left ventral edge.

Similar use-wear patterns are visible on the right edge, but the use-wear traces are not as developed as they are on the left edge. In this case, the dorsal face is less affected by edge scarring than the ventral face. The scars are feather scalar and step trapezoidal (E), the polish is rough (D), but smooth polish was found on the mesial dorsal edge. This suggests that this edge was also used for whittling medium-hard material, likely wood or plants, but probably in a shorter duration than the left edge.

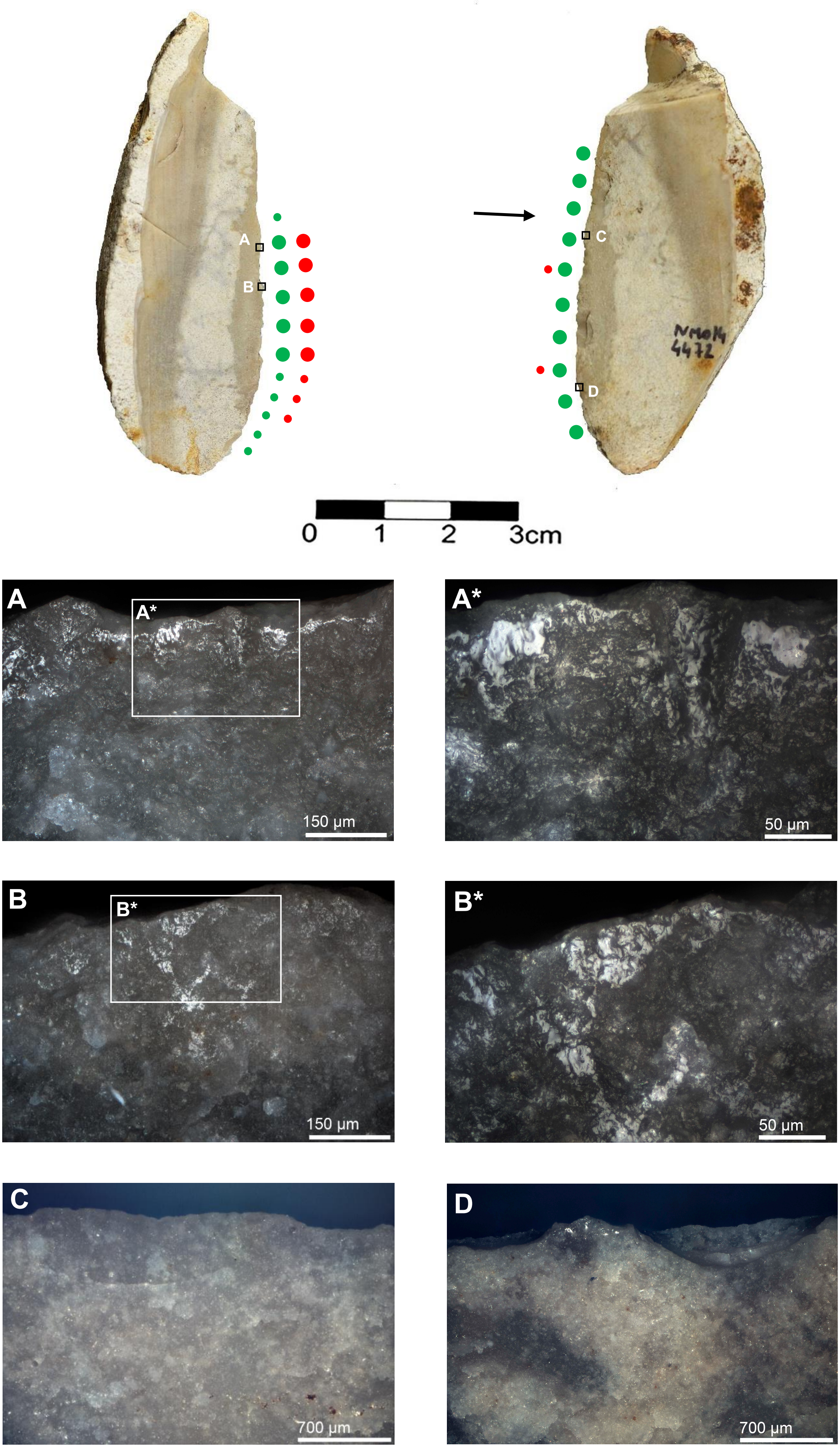
A
B
C
NMOM
4472
D
0
1
2
3cm
A
A*
150 µm
A*
50 µm
B
B*
150 µm
B*
50 µm
C
700 µm
D
700 µm

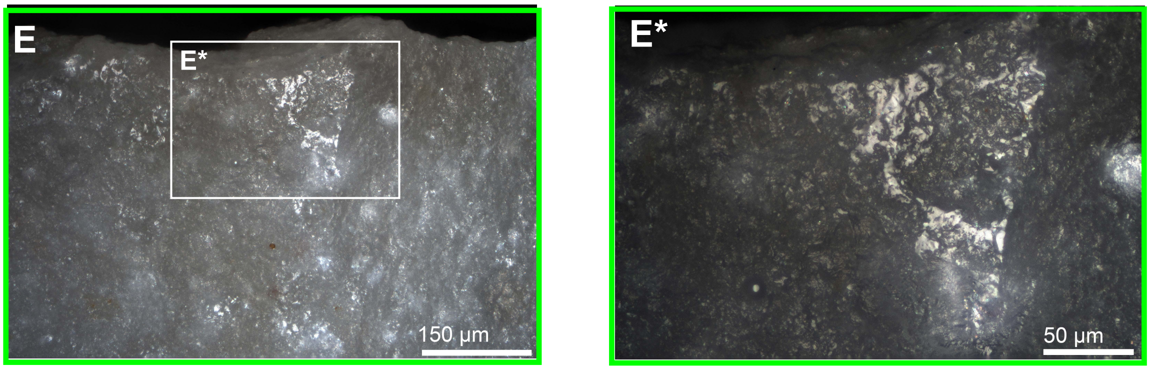

Supplementary figure S16. Naturally backed knife NMO14 4472 showing use-wear on the right edge. A, A*, smooth flat-wavy polish and light edge-rounding on the dorsal mesial right edge, 200x, 500x; B, B*, smooth-rough polish on the dorsal mesial right edge, 200x, 500x; C, step-terminating scar on the ventral mesial right edge, 50x; D, steep semi-circular scars with step terminations and polish spots on the elevated area, 50x; E, E*, experimental bone polish showing smooth-rough texture and wavy topography on the right dorsal edge of tool NMO-EXP43-H, used for scraping fresh cow bone (*Bos Taurus*), 200x, 500x, 2200 strokes, 30 min.

Interpretation

A well-developed pattern of step and feather-terminated scalar scars is visible on the mesial and proximal extremities on the right dorsal edge. These groupings of scars are intense on the mesial part where they are associated with well-developed smooth polish with flat-wavy topography (A, A*) and smooth-rough polish with the same topography (B, B*). The same wear features were observed in our experimental bone-scraping tools (E, E*). In the areas of most significant development, transversal waves or ripples are associated with polish, clearly indicating the use-motion (A*). The proximal part of the dorsal edge offers minor scarring and polish development. The ventral edge shows a more intense edge scarring in comparison with the dorsal face, indicating that the tool was used with an oblique working angle.

The ventral face does not show evidence of polish, apart from two specific areas on the mesial and proximal extremities (D). The scar pattern is similar to the dorsal face but more intense: step and feather terminated scalar scars are dominant, but they are much larger (C) and they are disposed in groupings and associated with more overlapping scars with fissured and step terminations (D). The different wear attributes visible on the edge and their distribution on both faces of the right edge indicate a transversal - negative – motion in hard animal material, likely bone.

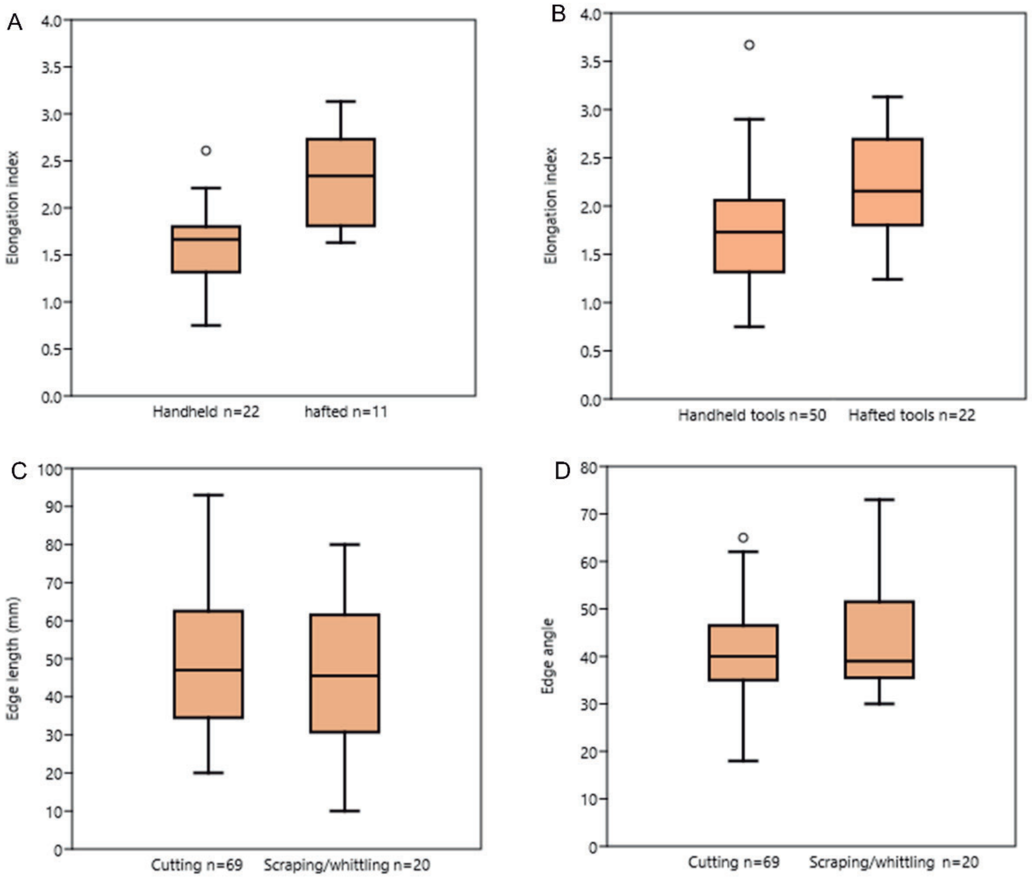

Supplementary figure S17. Box plots comparing: *A, the elongation index between hafted and handheld artifacts used in butchery; *B, the elongation index between hafted and handheld artifacts used in all activities; C, the length of the used edge according to the use-motion, longitudinal vs transversal activities; D, used edge angle according to use-motion. *The elongation index was calculated by dividing the artifact's length by its medial width.

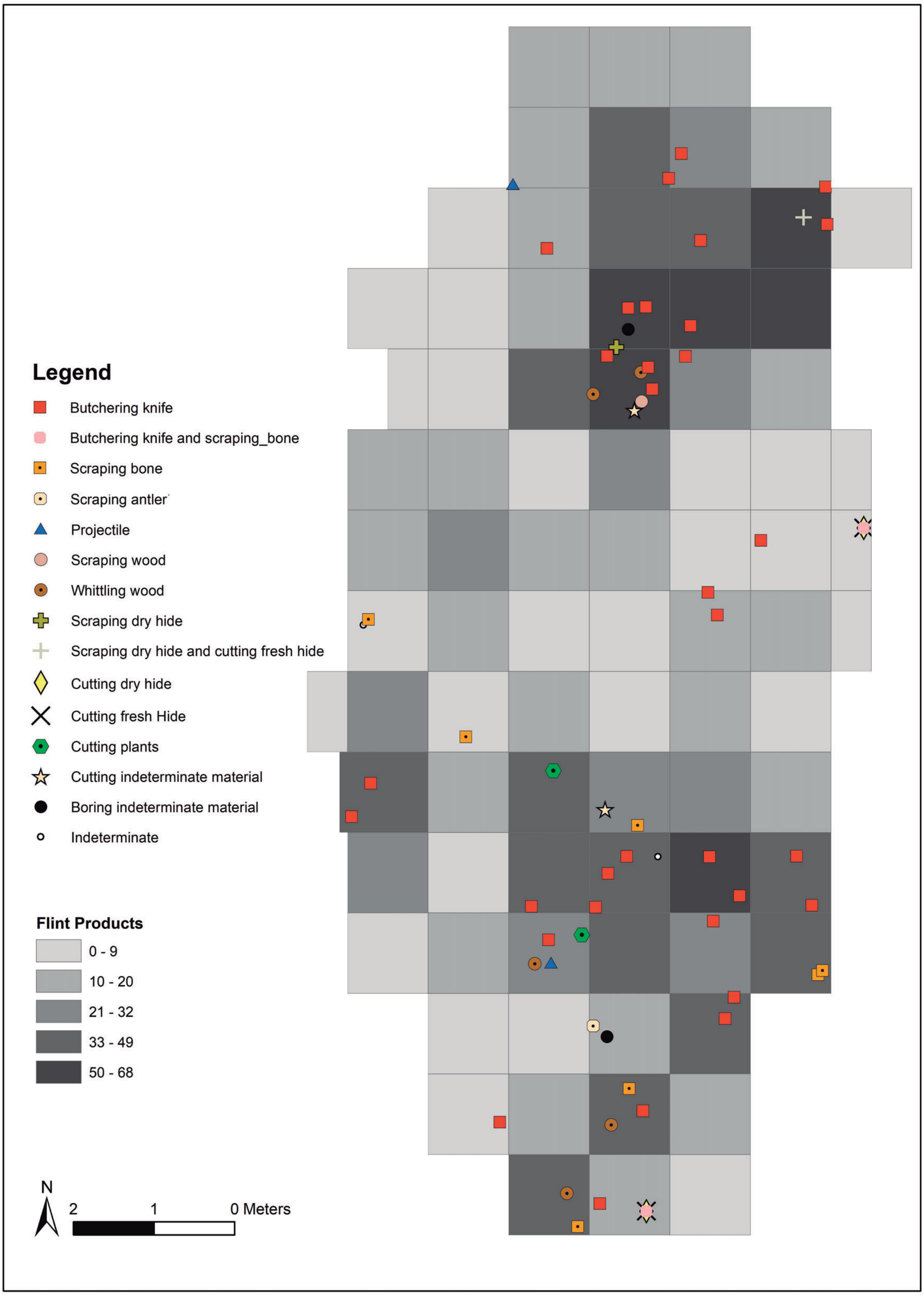

Supplementary figure S18.- Spatial plotting of the used artifacts of the sample on the excavated surface of area D considering the density of flint products.

Experimental wear

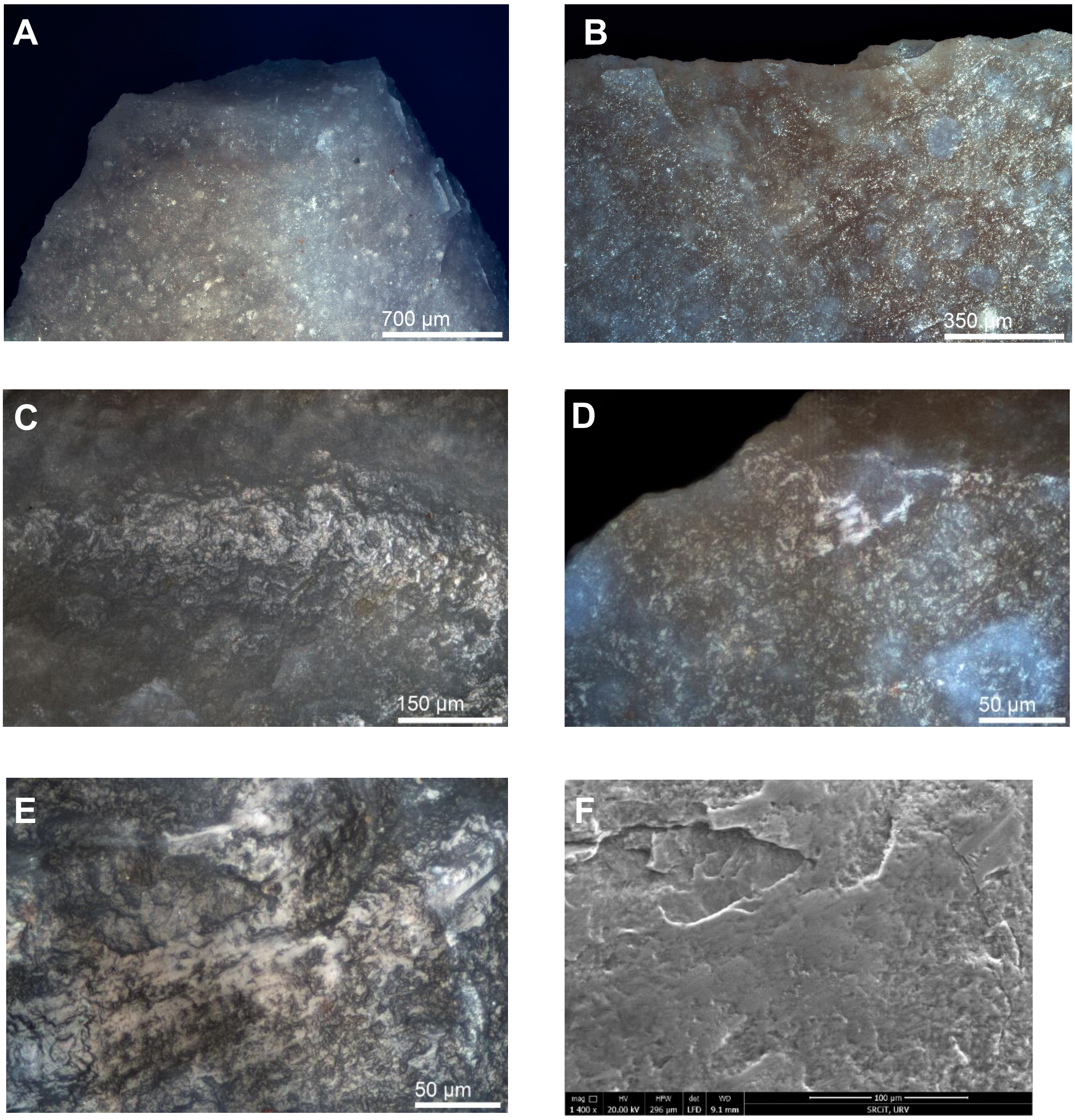

Supplementary figure S19. Use-wear on experimental tools used in butchery. A, Dorsal distal fracture on the tool NMO-EXP60-C used for defleshing two fresh cow bone epiphysis, 50x, 3465 strokes, 55 min; B, Feather scalar scars on the ventral distal right edge of tool NMO-EXP02-C used to eviscerate and disarticulate an adult red deer, 100x, 1800 strokes, 27 min; C, Rough-bright polish on the opposite face of the same tool from contact with soft animal tissue, 200x; D, Smooth polish spot and longitudinal striations from contact with bone on the dorsal distal edge of tool NMO-EXP06-C used to eviscerate and disarticulate an adult red deer, 500x, 2200 strokes, 44 min; E, Smooth dull polish and longitudinal striations from contact with medium-hard animal tissues (viscera, cartilages, etc.) on the dorsal distal right edge of tool NMO-EXP02-C, 500x; F, Previous polish observed with scanning electron microscopy (SEM) in which the fine striations are more visible, 1400x.

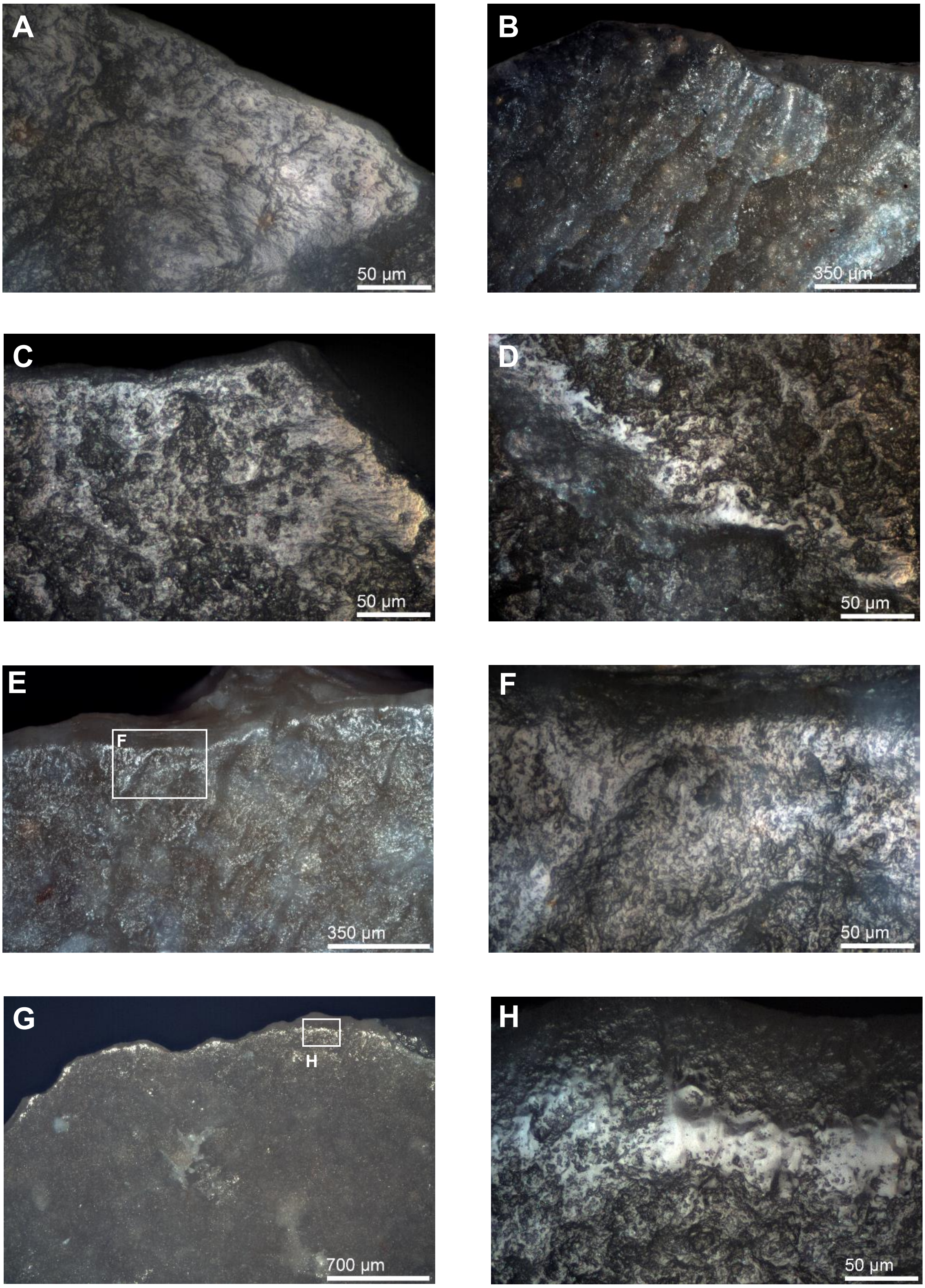

Supplementary figure S20. Use-wear on experimental tools used for processing fresh and dry hide. A, well-developed rough polish with dull appearance and edge rounding on the ventral distal right edge of tool NMO-EXP01-C used for skinning two adult red deer (*Cervus elaphus*), 500x, 5250 strokes, 2 h; B, invasive polish on the lancets on the ventral distal right edge of tool NMO-EXP62-P used for cutting fresh deer hide, 100x, 1350 strokes, 20 min; C, Rough-dull pitted polish showing gritty appearance, and associated with edge rounding and longitudinal striations on the ventral proximal left edge of tool NMO-EXP17-P used for cutting dry deer hide, 500x, 2720 strokes, 40 min; D, smooth polish surrounded by rough-dull polish on the ventral mesial left edge of the same tool, 500x; E, low invasive polish and edge rounding on the ventral mesial right edge of tool NMO-EXP08-P used for scraping fresh deer hide, 100x, 1500 strokes, 20 min; F, close-up of the previous image showing the fresh

hide polish, edge rounding and transversal striations, 500x; G, smooth polish band on the ventral distal right edge of tool NMO-EXP25-P used for scraping dry deer hide, 50x, 3172 strokes, 45 min; H, close-up of the previous image showing the smooth texture, wavy topography and transversal ripples of the polish associated with well-developed edge rounding, 500x.

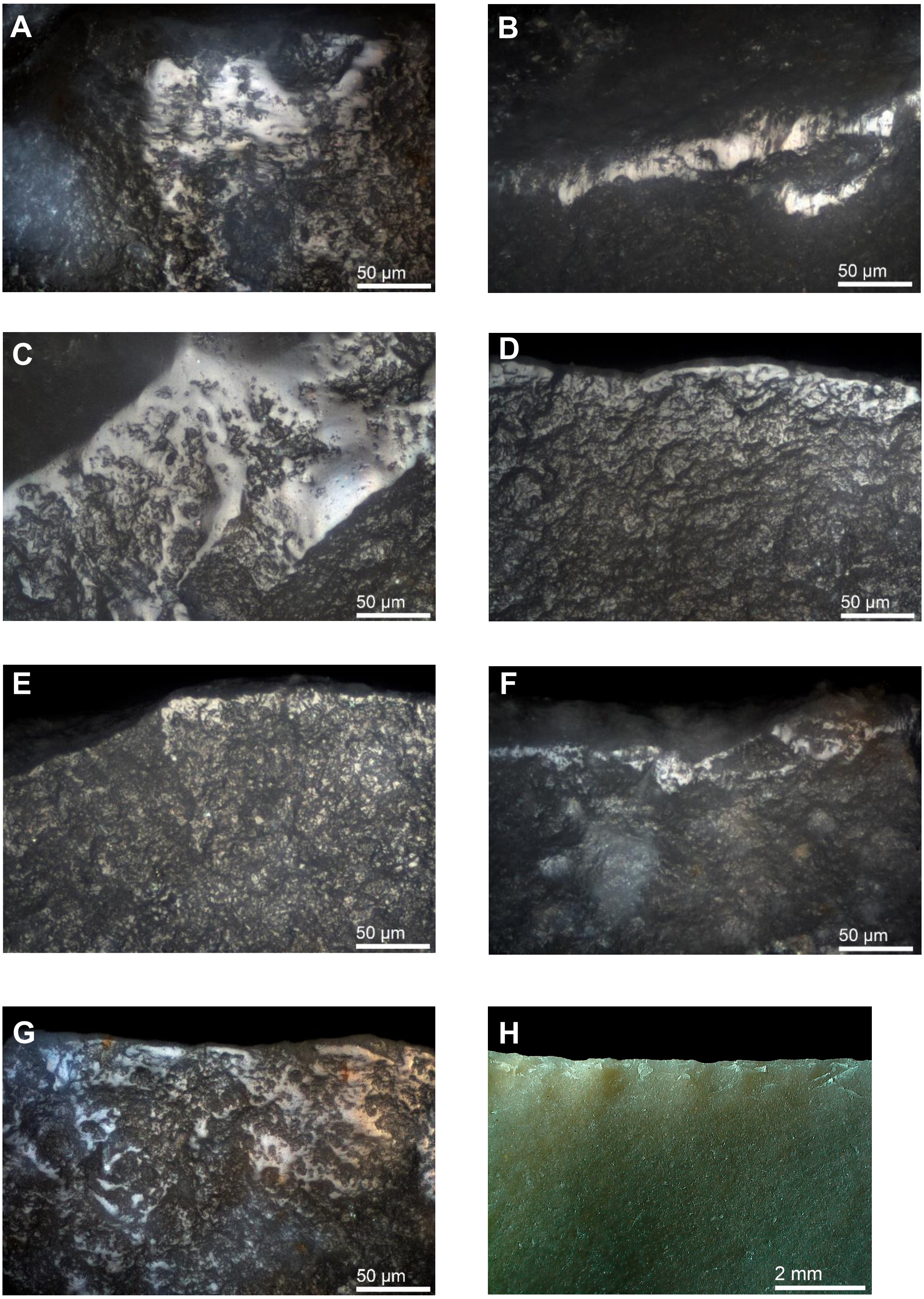

Supplementary figure S21. Use-wear on experimental tools used in a variety of activities. A, smooth-pitted polish with wavy-doomed topography and longitudinal ripples on the dorsal mesial left edge of tool NMO-EXP04-H used for sawing fresh cow bone, 500x, 5700 strokes, 35 min; B, polish bevel with transversal ripples on the ventral mesial right edge of tool NMO-EXP50-A used for scraping dry roe deer antler, 500x, 860 strokes, 10 min; C, smooth-pitted polish with doomed topography on the dorsal distal right edge of tool NMO-EXP12-V used for scraping fresh erect bamboo (sp.), 500x, 2000 strokes, 35 min; D, smooth polish bevel on the ventral mesial right edge of tool NMO-EXP13-V used for whittling fresh reed plants (*Cyperus papyrus),* 500x, 1624 strokes, 30 min; E, smooth polish with wavy topography and transversal striations on the ventral distal right edge of tool NMO-EXP23-M used for scra-

ping fresh hard wood (*Quercus ilex*), 500x, 1100 strokes, 20 min; F, smooth polish with doomed topography on the dorsal proximal left edge of tool NMO-EXP24-M used for scraping dry soft wood (*Salix sp*.), 500x, 2552 strokes, 40 min; G, smooth polish with doomed topography in its initial stages of linkage on the dorsal mesial right edge of tool NMO-EXP09-V used for cutting high-silica plants (*Arundo donax*), 200x, 1500 strokes, 20 min; H, overlapped scars showing crushing and fissured terminations on the ventral distal left edge of tool NMO-EXP43-H used for scraping fresh cow bone, 35 x, 2200 strokes, 30 min, HFOV 8.6 mm.

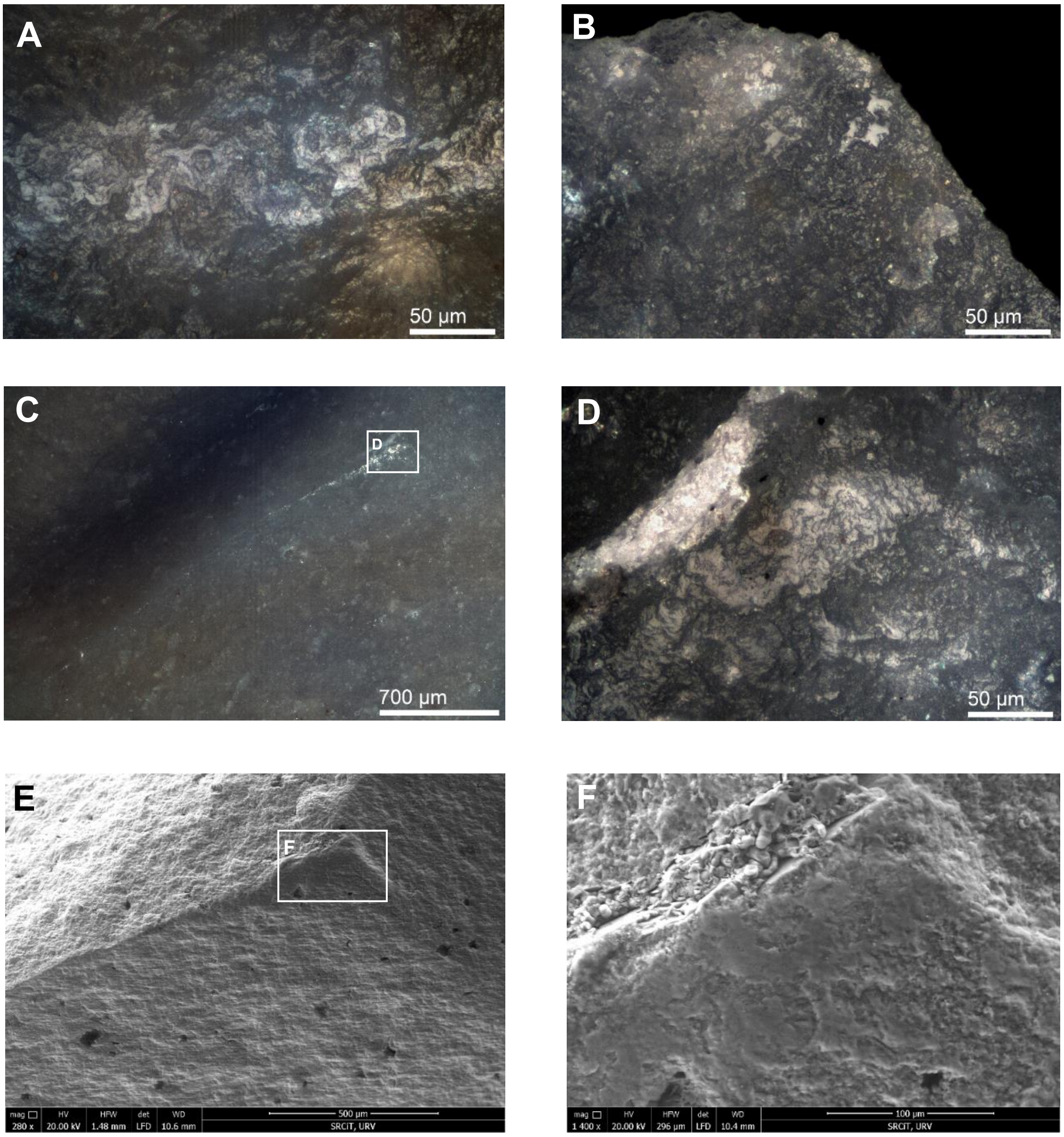

Supplementary figure S22. Hafting wear on experimental tools. A, hafting polish formed upon friction between hide particles and the tool surface within the haft on the dorsal ridge of tool NMO-EXP08-P used for scraping fresh deer hide while hafted in a male split wooden haft, 500x, 1500 strokes, 20 min; B, *hafting bright spot formed upon friction between the haft and the tool surface on the ventral distal right edge of tool NMO-EXP20-C used for cutting meat while hafted in a male split lateral wooden haft, 500x, 1800 strokes, 40 min. C, hafting polish on the dorsal proximal right ridge of tool NMO-EXP21-P used for scraping and cutting dry deer hide while hafted in a male split wooden haft and wrapped with vegetal bindings, 50x, 5700 strokes, 90 min; D, close-up of the hafting polish showed in C, likely formed by friction between hide particles and the tool surface within the haft, 500x; E, F, the same hafting polish observed with SEM. *Possibly formed by friction during the hafting process itself due to the low-pressure nature of the activity.

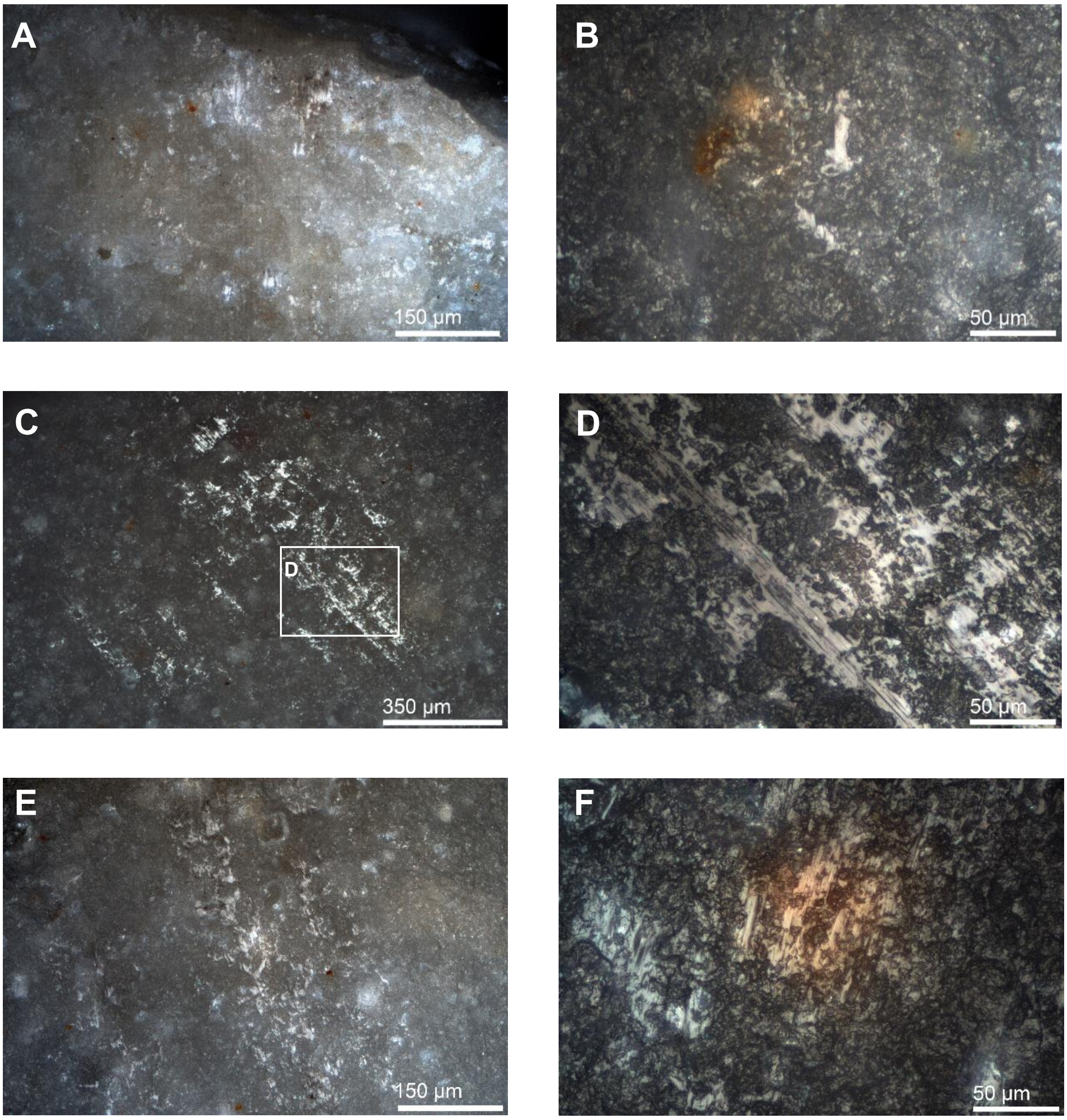

Supplementary figure S23. Production wear documented on experimental tools before use. A, knapping striation on the butt of tool NMO-EXP70-L, knapped with a limestone pebble, 200x; B, retouch polish striation on the dorsal distal edge of tool NMO-EXP55-A, retouched by pressure flaking with dry roe deer antler compressor, 500x; C, retouch polish striation on the left dorsal edge of tool NMO-EXP54-H, retouched on an anvil with a sandstone pebble, 100x; D, close-up of the previous retouch striation showing the longitudinal grooved polish which forms the striation, 500x; E, retouch polish striation on the right ventral edge of tool NMO-EXP59-A, retouched with a deer antler hammer, 200x; F, retouch polish striation on the ventral mesial right edge of tool NMO-EXP25-P, retouched with a sandstone pebble, 500x.
